# C−H Cyanation of 6‐Ring N‐Containing Heteroaromatics

**DOI:** 10.1002/chem.201703931

**Published:** 2017-09-22

**Authors:** Bryony L. Elbert, Alistair J. M. Farley, Timothy W. Gorman, Tarn C. Johnson, Christophe Genicot, Bénédicte Lallemand, Patrick Pasau, Jakub Flasz, José L. Castro, Malcolm MacCoss, Robert S. Paton, Christopher J. Schofield, Martin D. Smith, Michael C. Willis, Darren J. Dixon

**Affiliations:** ^1^ Department of Chemistry, Chemistry Research Laboratory University of Oxford 12 Mansfield Road Oxford OX1 3TA UK; ^2^ Global Chemistry UCB New Medicines, UCB Biopharma sprl 1420 Braine-L'Alleud Belgium; ^3^ Global Chemistry UCB 216 Bath Road Slough SL1 3WE UK; ^4^ Bohicket Pharma Consulting LLC 2556 Seabrook Island Road Seabrook Island South Carolina 29455 USA

**Keywords:** cyanation, heterocycles, late-stage functionalization, nitriles

## Abstract

Heteroaromatic nitriles are important compounds in drug discovery, both for their prevalence in the clinic and due to the diverse range of transformations they can undergo. As such, efficient and reliable methods to access them have the potential for far‐reaching impact across synthetic chemistry and the biomedical sciences. Herein, we report an approach to heteroaromatic C−H cyanation through triflic anhydride activation, nucleophilic addition of cyanide, followed by elimination of trifluoromethanesulfinate to regenerate the cyanated heteroaromatic ring. This one‐pot protocol is simple to perform, is applicable to a broad range of decorated 6‐ring N‐containing heterocycles, and has been shown to be suitable for late‐stage functionalization of complex drug‐like architectures.

The nitrile group is an important chemical motif commonly found in molecules throughout chemistry, biology, and medicine. In chemistry, it is a versatile intermediate that can act as precursor to a number of functional groups, such as amides, amines, and carboxylic acids, and to five‐ and six‐membered heterocycles through cycloaddition or condensation reactions.^1^ In medicine, the polarity, directionality, and low molecular weight of the nitrile moiety make it a substituent of choice in SAR studies, both as a diverse isostere capable of precise hydrogen bonding, and as a beneficial modifier of physicochemical properties.^2^ As a result of these properties, it is found attached to N‐containing heterocycles in a host of important pharmaceuticals including the tyrosine kinase inhibitor bosutinib (**1**), xanthine oxidase inhibitor topiroxostat (**2**), and MIV‐150 (**3**), a non‐nucleoside reverse transcriptase inhibitor (Scheme 1 a). Owing to their synthetic relevance and prevalence in biologically active heterocyclic compounds, there is demand for new and effective methods to install this important functional group. To this end, recent efforts have largely focused on the discovery of metal‐catalyzed coupling procedures,^3^ as well as the invention of direct C−H activation protocols.^4^ Despite the many advances in the field, we recognized that the development of a complementary and direct C−H cyanation protocol that was general to many classes of N‐containing heterocycles would likely find application in library generation and target synthesis, and herein, we report our findings.

**Scheme 1:**
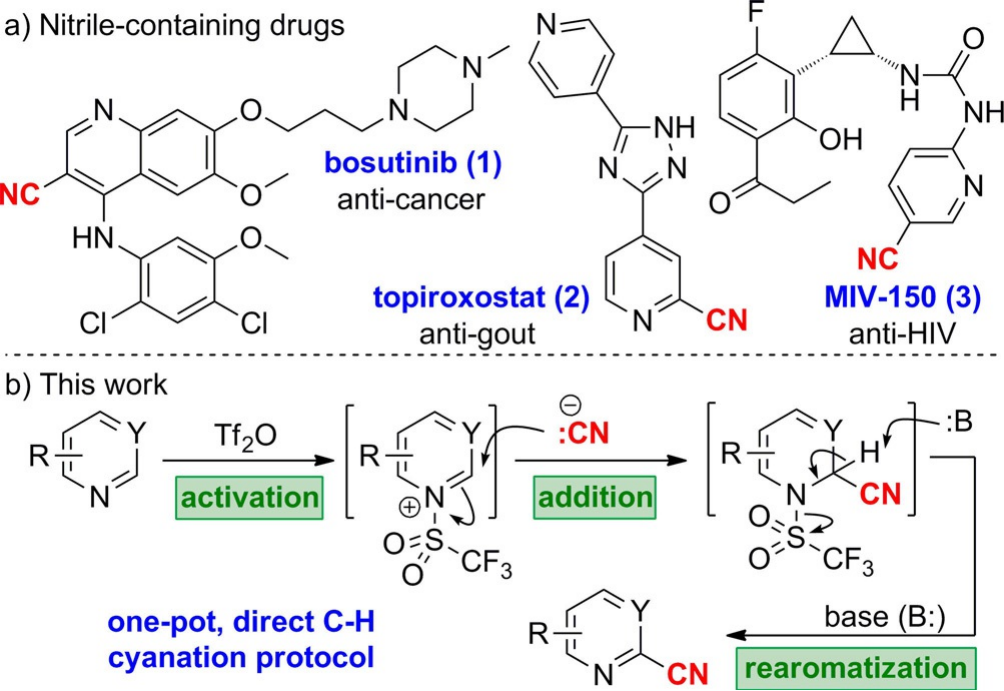
(a) Nitrile‐containing drugs: bosutinib (**1**), tyrosine kinase inhibitor; topiroxostat (**2**), xanthine oxidase inhibitor; MIV‐150 (**3**) non‐nucleoside reverse transcriptase inhibitor. (b) Proposed three‐stage protocol for one‐pot C−H cyanation.

Our plan was to exploit the nucleophilicity of the N‐atom contained within various 6‐ring heterocycles as a general activation mode to introduce the nitrile moiety. Following initial nucleophilic addition to a suitable electrophilic species (such as triflic anhydride), the heteroaromatic ring would be activated towards subsequent nucleophilic attack of cyanide and concomitant base mediated elimination of trifluoromethanesulfinate anion could generate the cyanated heteroaromatic ring (Scheme 1 b).^5^ The activation of N‐containing heteroaromatic species by reaction of various electrophiles at nitrogen has previously been deployed to effect a range of transformations, and examples of carbon‐, phosphorus‐, and sulfur‐based activating groups have been reported.^6^ We hoped to build on this approach to develop a mild and convenient one‐pot cyanation protocol that would be both general to many 6‐ring N‐containing heterocycles and useful for late‐stage functionalization of drug‐like molecules.

2‐Phenylpyridine (**4**) was chosen as a model substrate to enable us to study reactivity and product isomeric ratio distribution in the planned three‐stage, one‐pot cyanation protocol. An initial survey of variables, such as electrophilic activator, cyanide source, and base was performed. Inspired by the work of Corey, triflic anhydride (Tf_2_O) was found to be uniquely effective as an activator,^7^ whereas trimethylsilyl cyanide was identified as the most convenient and effective cyanide source for productive C−C bond formation. Following full consumption of the activated pyridinium species, addition of *N*‐methylmorpholine (NMM) ensured conversion to the final, heteroaromatic product. By using this combination of reagents, nitrile **5** was produced as a mixture of isomers in moderate yield, but importantly in the absence of any non‐aromatized intermediates (Table 1, entry 1). The timing and temperature of the different steps in the procedure were found to be important for reaction yield: delaying addition of base (entry 2) significantly improved production of **5**, with further improvement afforded by warming the first step of the reaction from −78 °C to room temperature (entry 3). Switching to higher boiling solvents allowed us to assess the effect of temperature in the rearomatization step (entries 4, 5 and 6), and CHCl_3_ at 60 °C was identified as optimal, with high yield retained upon scale up (entry 7). It is noteworthy that the ratio of **5**/**5′** remained essentially constant throughout our studies despite temperature and solvent variation, with a slight preference for cyanation in the 4‐position over the 6‐position (**5**/**5′**; ≈60:40). Products of cyanation at the 2‐, 3‐, or 5‐position of **4** were not observed.

**Table 1:** Selected reaction optimization data.^[a]^

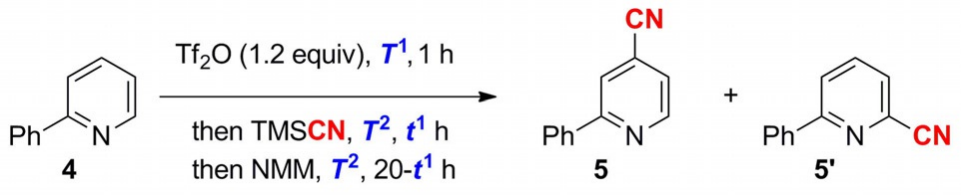						
Entry	*T* ^1^ [°C]	*T* ^2^ [°C]	*t* ^1^ [h]	Solvent	Yield [%]^[b]^	Ratio **5**:**5′** ^[c]^
1	−78	40	0	CH_2_Cl_2_	30	53:47
2	−78	40	3	CH_2_Cl_2_	61	59:41
3	21	40	3	CH_2_Cl_2_	75	61:39
4	21	110	3	PhMe	59	62:38
5	21	80	3	DCE	69	61:39
6	21	60	3	CHCl_3_	80	61:39
7^[d]^	21	60	3	CHCl_3_	74	59:41

[a] Conditions: Tf_2_O (1.2 equiv) added to substrate (100 mg) in solvent (0.1 m) at *T* ^1^, stirred 1 h, then TMSCN (5.0 equiv) was added, warmed to *T* ^2^ for *t* ^1^. NMM (1.3 equiv) was added, stirred for 20−*t* ^1^ h before quenching with NaHCO_3_. Fully rearomatized in each case. [b] Isolated yield. [c] Determined following isolation by flash column chromatography. [d] Performed on 1 g scale.

With an optimized general procedure in hand, the substrate scope of the cyanation was investigated (Scheme 2). Pyridines are extremely widespread motifs in bioactive compounds, both in nature and in the clinic, and as such methods for their functionalization are highly sought after. We were delighted to see that this practical, one‐pot protocol was effective across a diverse range of heteroaromatic ring systems, giving aromatic cyanated products in up to 96 % yield (Scheme 2 a). Substrates possessing aromatic (**5**, **6**) and electron‐withdrawing (**8**–**13**) substituents gave excellent yields, with no competitive addition to the ester group in **11** observed. Halide (**14**, **15**) and alkyl (**7**, **16**–**19**) substituents were also well‐tolerated in various positions. The alkyl substituents demonstrate compatibility with electron‐donating groups, whereas the halopyridines are particularly noteworthy for their potential for further downstream orthogonal transformations. Of the pyridines surveyed, substitution was generally favored at the 4‐position, with minor, separable quantities of other isomers produced in some cases. The isolation of these additional compounds could be valuable as further analogues for inclusion in SAR studies. Quinolines and isoquinolines represent an important class of ring system in medicinal chemistry and accordingly were attractive substrates to investigate.^8^ Pleasingly, cyanated quinolines **20**–**22** were produced in excellent yields (Scheme 2 b), with substitution at the 2‐position predominating (a reversal of the selectivity observed for pyridines). Isoquinolines were also highly effective substrates yielding fully rearomatized 1‐substituted nitriles **23** and **24** as single isomers. Diazines (pyridazine, pyrimidine, and pyrazine) represent a diverse class of heterocycle where the presence of the second nitrogen atom in the six‐membered ring provides electronically distinct examples; it is thus not unsurprising that no single reported cyanation procedure is applicable to members of each class. However, our protocol proved to be successful across a diverse range, with cyanated pyridazine (**25**–**27**, **34**), pyrimidine (**28**–**30**, **35**), and pyrazine (**31**–**33**) examples produced in moderate to excellent yields (Scheme 2 c). Bidentate ligands are ubiquitous in organometallic chemistry, and new methods to modify and tune them are in constant demand. We observed straightforward cyanation of **36**–**38**, with only traces of di‐cyanated products observed for **36**, allowing facile synthesis of useful unsymmetrical cyanated ligands.

**Scheme 2:**
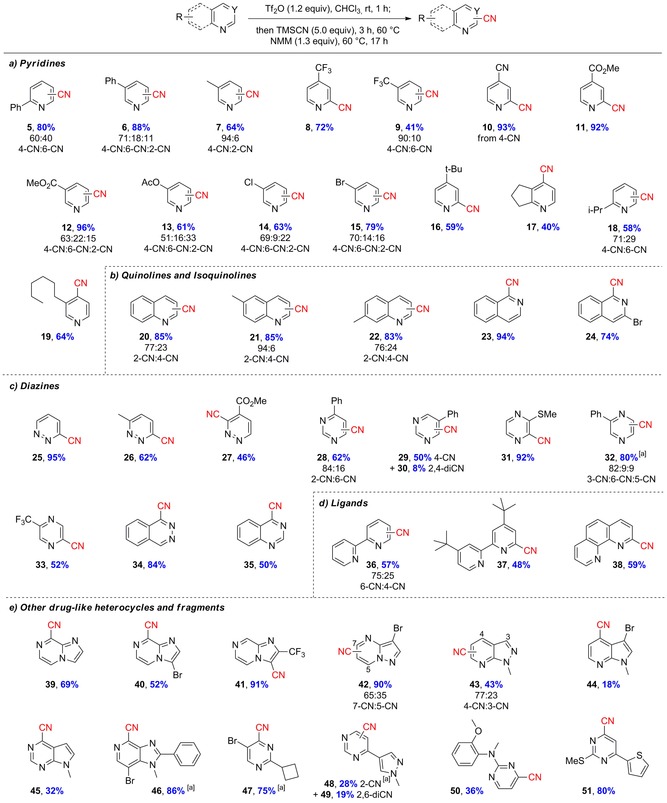
Substrate scope. Conditions: 0.7 mmol scale, Tf_2_O (1.2 equiv), CHCl_3_ (0.1 m wrt substrate), 1 h, RT; then TMSCN (5.0 equiv), 3 h at 60 °C; then NMM (1.3 equiv), 17 h at 60 °C. Yields are of isolated materials. Isomeric ratio was determined from isolated amounts of purified material. For consistency, numbering of functionalized carbon refers to the starting material. [a]<0.7 mmol scale, see the Supporting Information for details.

To further demonstrate the general applicability of our methodology, we examined more complex heterocyclic structures, which are commonly found in commercial pharmaceuticals and agrochemicals (Scheme 2 e).^8^ A series of cyanated heterocycles were produced (**39**–**51**), with excellent yields obtained in the presence of halides (**40**, **42**, **44**, **46**, **47**), and non‐reactive heterocycles (**48**, **49**, **51**). The production of cyanated analogues of such diverse heterocyclic structures demonstrates the power of this methodology as a tool for medicinal chemistry, and it is worth noting that all of the examples described in Scheme 2 were produced by using a single experimental protocol.

Finally, we sought to demonstrate the potential of the methodology for the late‐stage functionalization of complex substrates, a concept that is becoming increasingly popular (Scheme 3).^9^, ^10^ By using the standard protocol, we were able to cyanate abiraterone acetate (**52**, a treatment for prostate cancer) in excellent yield with three different analogues produced. Protected erlotinib (**53**, a treatment for lung and pancreatic cancer) gave a good yield of a single isomer, whereas mirtazapine (**54**, an antidepressant) gave a moderate yield of the 4‐CN derivative in the presence of electron‐donating groups on the pyridine moiety. Quinine was included on the World Health Organization's List of Essential Medicines for its important antimalarial properties; following *O*‐methylation, it was cyanated in good yield to provide **55** as a single regioisomer. As well as further extending the scope, the latter two examples demonstrate the tolerance of the method to basic amine functionality, although we hypothesize that competitive and reversible coordination of these groups to the activator gave low conversion in the case of **54**.

**Scheme 3:**
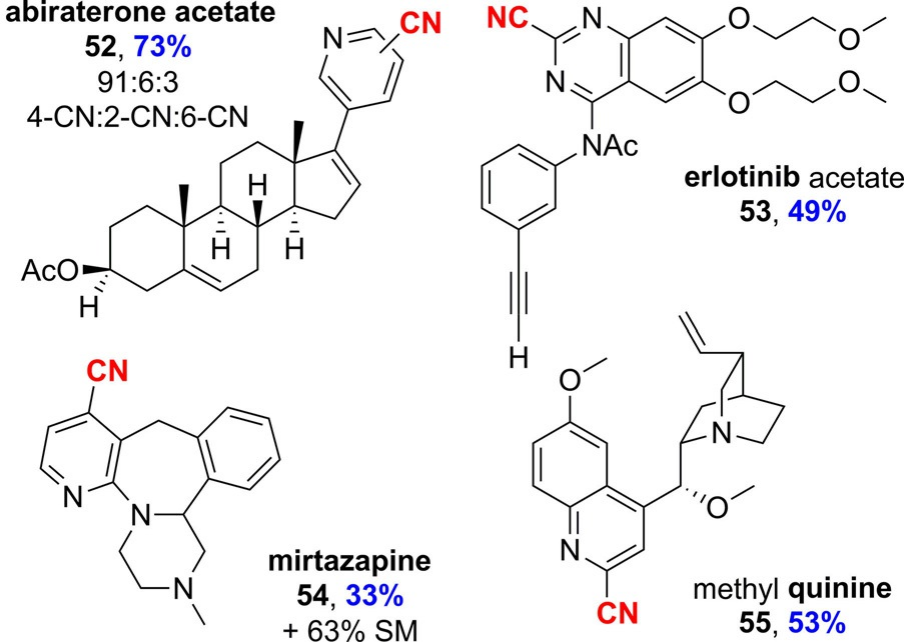
Late‐stage cyanation of drug and natural‐product derivatives. Conditions: Tf_2_O (1.2 equiv), CHCl_3_ (0.1 m wrt substrate), 1 h, RT; then TMSCN (5.0 equiv), 3 h at 60 °C; then NMM (1.3 equiv), 17 h at 60 °C. Isomeric ratio was determined from amounts of purified material.

We performed DFT (M06‐2X/def2‐SVP) calculations to understand the site‐selectivity of cyanation in terms of each heterocycle's electronic structure.^11^ We found that the condensed Fukui index, *f* ^*+*^(*r*), is a good quantitative predictor for the position(s) of attack and the level of selectivity observed.^12^ A large value of *f* ^*+*^(*r*) for a given carbon atom is associated with a large change in chemical potential when electron density is added—it indicates greater susceptibility towards nucleophilic attack. Functionalization at the most electrophilic position(s) of the *N*‐triflyl cation is consistent with irreversible, selectivity‐determining nucleophilic attack by cyanide. For 29 of the substrates studied experimentally, the largest Fukui index predicts the major site of cyanation 23 times; in the other six cases, cyanation occurs at the second most electrophilic position. This is attributable to the absence of steric effects in this electronic model (see Figures S2 and S3 in the Supporting Information). Differences in atomic Fukui indices match the experimental regioselectivity values well (particularly for pyridines and quinolines/isoquinolines; Figure 1).

**Figure 1:**
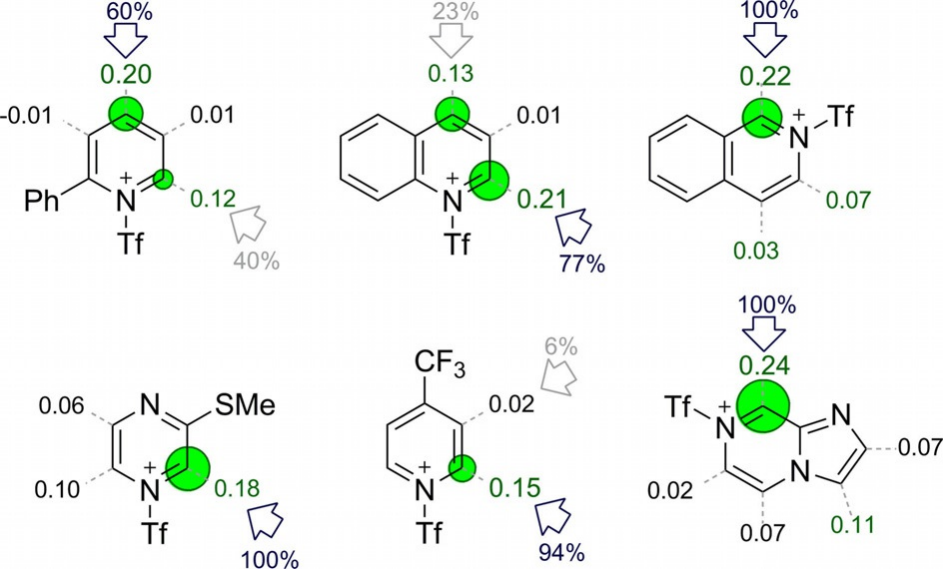
Magnitude of computed atomic Fukui indices for *N*‐triflyl heterocycles, *f* ^*+*^(*r*) (green circles), correlate with the site of nucleophilic attack (blue/grey arrows). Results for several other substrates are shown in the Supporting Information.

We also computed ^13^C NMR chemical shifts of the activated pyridines. The most deshielded nucleus matches the site of nucleophilic addition observed in the major (or single) regioisomer formed (Figure S4 in the Supporting Information). This is consistent with the results obtained by using condensed Fukui indices and supports the mechanistic interpretation above. Chemical shifts of other heterocycles do not match well with the observed sites of addition (unlike the Fukui index), presumably due to magnetic effects unrelated to chemical reactivity. Therefore, we focus on the use of Fukui indices, which can be employed by end‐users of this methodology to predict site‐selectivity in late‐stage heterocycle cyanation. These calculations consider the substrate only and do not require more laborious transition state modelling.

In conclusion, we have developed a direct and general one‐pot protocol that enables C−H cyanation of a wide range of 6‐ring N‐containing heterocycles. The procedure is easy to perform and is suitable for late stage functionalization of drug derivatives and advanced intermediates. Work to further expand the generality of this C−H functionalization approach is ongoing, and the results will be reported in due course.

## Experimental Section

Triflic anhydride (1.2 equiv) was added dropwise to a 0.1 m solution of the substrate (1.0 equiv) in anhydrous CHCl_3_ in a vial capped with a septum at room temperature under an atmosphere of argon or nitrogen. The resulting solution was stirred for 1 h, then trimethylsilyl cyanide (5.0 equiv) was added. The septum was exchanged for a screw cap, and the mixture was stirred at 60 °C for 3 h. The reaction vessel was removed from the heating source, and *N*‐methylmorpholine (1.3 equiv) was added quickly before the vial was resealed. The mixture was then stirred at 60 °C for a further 17 h before cooling to room temperature and quenching with saturated NaHCO_3_ solution. The phases were separated, and the organic phase was extracted twice with CH_2_Cl_2_. The combined organic phase was dried (MgSO_4_) and concentrated in vacuo. The crude residue was purified by silica flash column chromatography.

## Conflict of interest

The authors declare no conflict of interest.

## Supporting information

As a service to our authors and readers, this journal provides supporting information supplied by the authors. Such materials are peer reviewed and may be re‐organized for online delivery, but are not copy‐edited or typeset. Technical support issues arising from supporting information (other than missing files) should be addressed to the authors.

Supplementary
