# Supplementary material for: C−H Cyanation of 6‐Ring N‐Containing Heteroaromatics
Source: Chemistry. 2017 Sep 22;23(59):14733–7. doi: 10.1002/chem.201703931 (PMC5813275; doi:10.1002/chem.201703931)
Supplement: Supplementary file 1 — Supplementary [file CHEM-23-14733-s001.pdf]

CHEMISTRY
A European Journal

Supporting Information

C–H Cyanation of 6-Ring N-Containing Heteroaromatics

Bryony L. Elbert,[a] Alistair J. M. Farley+,[a] Timothy W. Gorman+,[a] Tarn C. Johnson+,[a] Christophe Genicot,[b] Bénédicte Lallemand,[b] Patrick Pasau,[b] Jakub Flasz,[c] José L. Castro,[c] Malcolm MacCoss,[d] Robert S. Paton,*[a] Christopher J. Schofield,*[a] Martin D. Smith,*[a] Michael C. Willis,*[a] and Darren J. Dixon*[a]

chem_201703931_sm_miscellaneous_information.pdf

Supporting Information

General Experimental S3

Additional Details of Optimization Studies S4

Synthesis and Characterisation of Non-Commercial Starting Materials S7

Synthesis and Characterisation of Heterocyclic Nitriles S10

NMR Spectra of Novel Compounds S41

Computational Results S76

References S91

General Experimental

Solvents and Reagents. Chloroform, dichloromethane, tetrahydrofuran, and toluene were obtained anhydrous from MBRAUN SPS5 solvent dispenser units having been passed through an activated alumina column under argon. 1,2-Dichloroethane and dioxane were purchased as anhydrous solvents in Sure/Seal™ bottles from Sigma Aldrich and used as received. Petroleum ether refers to the fraction which boils in the range 40-60 ºC. TMSCN refers to trimethylsilyl cyanide. Tf_2O refers to trifluoromethanesulfonic (triflic) anhydride. DCE refers to 1,2-dichloroethane. $NaHCO_3$ was used as a saturated aqueous solution. Tf_2O was obtained from Fluorochem and used as received. All cyanation substrates were obtained commercially and used as received unless otherwise indicated.

Reactions. All reactions were carried out under argon or nitrogen atmosphere unless otherwise stated. Oven-dried glassware was used for all reactions.

Chromatography. Thin-layer chromatography was performed on Merck aluminium-backed DC 60 F254 0.2 mm precoated plates, which were visualised with UV fluorescence and staining with potassium(VII) manganate or vanillin. Flash column chromatography was performed on a Biotage Isolera with Biotage ZIP or ZIP Sphere® Silica cartridges.

Melting points. Melting points were determined using a Griffin melting point apparatus and are uncorrected.

Infrared Spectroscopy. Infrared spectra were recorder on a Bruker Tensor 27 Fourier transform spectrometer, as a thin film on a diamond ATR module.

NMR Spectroscopy. ^{1}H NMR spectra were recorded at 400 or 500 MHz on a Bruker AVIIIHD 400, Bruker AVII 500, or Bruker AVIIIHD 500 respectively. ^{13}C NMR spectra were recorded at 100 MHz or 125 MHz on a Bruker AVIIIHD 400, Bruker AVIIIHD 500 or a Bruker AVII 500 with ^{13}C cryoprobe, respectively. Chemical shifts (δ_H and δ_C) are expressed in parts per million (ppm), referenced to the residual solvent peak of $CDCl_3$. Coupling constants (*J*) are reported to the nearest 0.1 Hz. Spectra are assigned based on chemical shift, coupling constants, COSY, HSQC and HMBC data and comparison with similar compounds. Splitting patterns are described using the following abbreviations: s (singlet), d (doublet), t (triplet), q (quartet), quin. (quintet), sept. (septet). (Q) indicates a quaternary carbon. Atoms are generally numbered according to IUPAC conventions, unless otherwise indicated. Deviation from IUPAC numbering is employed to facilitate consistent assignment of compounds.

Mass spectroscopy. Low-resolution mass spectra (*m/z*) were performed on a Micromass LCT Premier Open Access. High-resolution mass spectra were recorded under ESI or EI conditions on a Bruker MicroTOF.

Compound names. Compounds were generated by ChemBioDraw 2015, using IUPAC recommended conventions.

Additional Details of Optimization Studies

We began our optimization studies with 2-phenylpyridine, using Tf_2O as activator (Table S1). We were encouraged to observe successful cyanation with TMSCN (Table S1, entry 1), albeit in low yield and accompanied by significant *N*-ethylation of the substrate. Other cyanide sources ($Zn(CN)_2$, NaCN, and $K_4[Fe(CN)_6]$) gave no traces of product. Removal of Et_3N base from the reaction mixture prevented deleterious side product formation, although traces of unaromatised material were observed in crude reaction mixtures (Table S1, entry 2), while increasing TMSCN equivalents improved yield (Table S1, entries 3 and 4). In order to achieve full rearomatization, DTBMP was employed, with a good yield of cyanation observed (Table S1, entry 6). Use of a slight excess of Tf_2O and base further improved matters (Table S1, entry 7). A survey of other activators found Tf_2O to uniquely effective (Table S2).

Table S1. Initial optimization.[a]

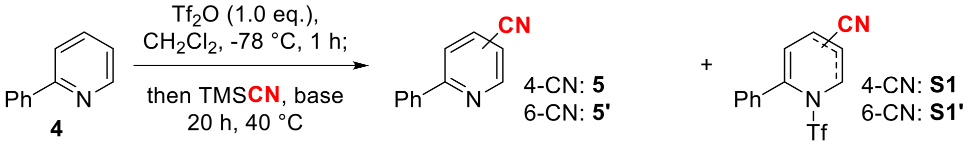

Entry	Eq. [CN]	Base (eq.)	Yield[b]	Ratio **5**:**5'**[c]	Side products[d]
1	1.1	Et_3N (1.0)	12%	50:50	*N*-Ethyl-2-phenylpyridinium, 65%
2	1.0	-	34%	59:41	Trace **S1** + **S1'**
3	2.0	-	45%	62:38	Trace **S1** + **S1'**
4	5.0	-	56%	61:39	12% **S1** + 4% **S1'**
5	5.0	Cs_2CO_3 (1.0)	48%	60:40	Not observed
6	5.0	DTBMP (1.0)	51%	66:34	Trace **S1** + **S1'**
**7**[e]	**5.0**	**DTBMP (1.3)**	**66%**	**56:44**	**Trace S1 + S1'**

[a] Conditions: Tf_2O added to substrate (100 mg) in CH_2Cl_2 (0.1 M) at -78 °C, stirred 1 h, then TMSCN and base added, warmed to 40 °C for 20 h before quench with $NaHCO_3$. [b] Isolated yield. [c] Determined by ^1H NMR analysis of product isolated following flash column chromatography. [d] Determined by ^1H NMR analysis of crude material. [e] 1.2 eq. Tf_2O. DTBMP = 2,6-di-*tert*-butyl-4-methylpyridine.

Table S2. Survey of Activators.[a]

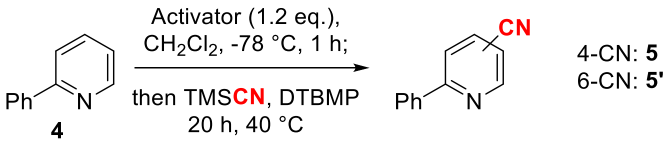

Entry	Activator	Yield[b]	Ratio **5**:**5'**[c]
1	Tf_2O	66%	56:44
2	TsCl	0%	-
3	NsCl	0%	-
4	MsCl	0%	-
5	Ts_2O	0%	-
6	TfOH	0%	-
7	TfOTMS	0%	-
8	HBF_4•OEt_2	0%	-
9	BF_3•OEt_2	0%	-
10	Ac_2O	0%	-
11	TFAA	0%	-
12	AcCl	0%	-
13	MeO_2CCl	0%	-

[a] Conditions: Activator (1.2 eq.) added to substrate (100 mg) in CH_2Cl_2 (0.1 M) at -78 °C, stirred 1 h, then TMSCN (5.0 eq.) and DTBMP (1.3 eq.) added, warmed to rt for 20 h before quench with $NaHCO_3$. [b] Isolated yield. [c] Determined by ^{1}H NMR analysis of product isolated. [d] Determined by ^{1}H NMR analysis of crude material. DTBMP = 2,6-di-*tert*-butyl-4-methylpyridine. N.R. = no reaction.

With conditions in hand (Table S1, entry 7), a brief screen of varied heterocyclic substrates was conducted (Scheme S1). However, our conditions proved unsuccessful, with incomplete rearomatization following cyanide addition observed in several cases. Hypothesizing that DTBMP was insufficiently basic to rearomatise a broad range of substrates, a series of bases were screened against several diverse substrates (Table S4). *N*-Methylmorpholine (NMM) was identified as the best compromise, albeit with cyanation of 2-phenylpyridine the least efficient. This lower yield was, however, an ideal starting point for further optimization, as shown in Table 1 in the Main Text.

Scheme S1. Unsuccessful early scope with initial conditions.

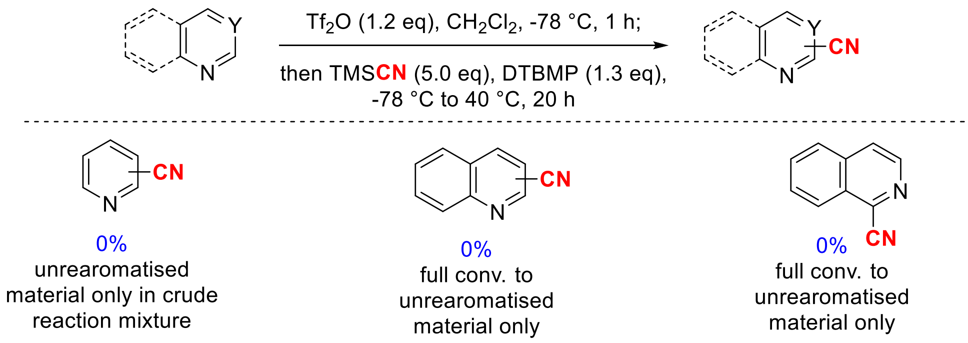

Table S4. Base vs substrate screen.[a]

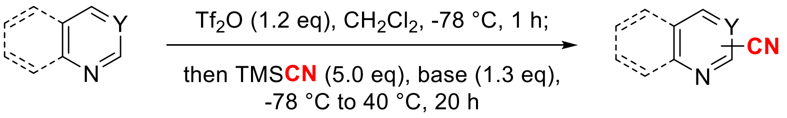

Base→<br>Substrate↓	DTBMP	3-quinuclidinol	3-quinuclidinyl acetate	**NMM**	1,2,2,6,6-pentamethylpipe ridine
2-Phenyl pyridine	66%<br>56:44<br>4-CN:6-CN	0%<br>SM only	0%<br>SM only	**30%**<br>**57:43**<br>**4-CN:6-CN**[b]	31%<br>58:42<br>4-CN:6-CN
Quinoline	0%<br>(full conversion to unrearom. product)	89%<br>82:18<br>2-CN:4-CN	73%<br>74:26<br>2-CN:4-CN	**99%**<br>**85:15**<br>**2-CN:4-CN**	64%<br>84:16<br>2-CN:4-CN
Isoquinoline	0%<br>(full conversion to unrearom. product)	0%<br>(full conversion to unrearom. product)	39%<br>Single isomer<br>+ 32% unrearom. product	**84%**<br>**Single isomer**	0%<br>(part. conversion to unrearom. product)

[a] Conditions: Tf_2O added to substrate in CH_2Cl_2 (0.1 M) at -78 °C, stirred 1 h, then [CN] and base added, warmed to 40 °C for 20 h before quench with $NaHCO_3$. [b] This result also seen in Main Text Table 1, entry 1. % shows isolated yield, ratios determined by ^{1}H NMR analysis of crude reaction mixtures and products isolated.

Synthesis and Characterisation of Non-Commercial Starting Materials

N-(2-Methoxyphenyl)pyrimidin-2-amine (S2)

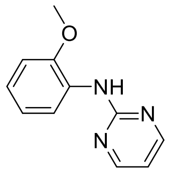

Prepared according to a literature procedure.[1] To a flask charged with 2-aminopyrimidine (418 mg, 4.40 mmol, 1.1 eq.), copper iodide (381 mg, 2.0 mmol, 0.5 eq.) and K_2CO_3 (1.11 g, 8.0 mmol, 2 eq.) was added a solution of 2-bromoanisole (748 mg, 4.0 mmol) and DMEDA (0.22 mL, 2.0 mmol, 0.5 eq.) in anhydrous dioxane (20 mL). After stirring at 100 °C for 24 h the mixture was allowed to cool to room temperature and brine (20 mL) was added. The solution was extracted with ethyl acetate (3 x 20 mL) and the combined organic extracts were washed with ammonium hydroxide (60 mL), dried ($MgSO_4$), filtered and the volatiles were removed under reduced pressure. Column chromatography on silica (0→100% ethyl acetate in petroleum ether) yielded the titled compound as a colorless oil (394 mg, 1.96 mmol, 49%).

***R*$_f$** 0.26 (20% ethyl acetate in petroleum ether); **^{1}H NMR (400 MHz, $CDCl_3$)** δ 8.52-8.47 (1H, m, H3'), 8.42 (2H, d, *J* 4.8 Hz, H4 and H6), 7.77 (1H, broad s, N*H*), 7.03-6.94 (2H, m, H4' and H5'), 6.91-6.87 (1H, m, H6'), 6.69 (1H, t, *J* 4.8 Hz, H5), 3.89 (3H, s, C*H*$_3$); **^{13}C NMR (100 MHz, $CDCl_3$)** δ 160.0 (Q), 157.9, 147.8 (Q), 129 (Q). 121.7, 120.8, 118.4, 112.3, 109.9, 55.6; **IR ν_{max}/cm^{-1} (neat)** 3417, 1577, 1522, 1443, 1401, 1242; **HRMS (ESI)** found *m/z* 202.09754 $[M+H]^+$, $C_{11}H_{12}N_3O$ requires *m/z* 202.09749. The spectroscopic data were found to be in agreement with that reported by Liu and co-workers.[1]

2-((2-Methoxyphenyl)(methyl)amino)pyrimidine (S3)

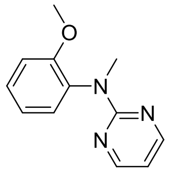

N-(2-Methoxyphenyl)pyrimidin-2-amine (507 mg, 2.52 mmol) in anhydrous THF (3 mL) was added to a suspension of sodium hydride (302 mg, 60 wt% in mineral oil, 7.56 mmol, 3 eq.) in anhydrous THF (2 mL) at 0 °C. After stirring at this temperature for 30 minutes iodomethane (0.31 mL, 5.0 mmol, 2 eq.) was added dropwise and the solution was allowed to warm to room temperature and stir overnight. The reaction was quenched by the addition of a saturated $NaHCO_3$ solution (5 mL) and the solution was extracted with ethyl acetate (3 x 5 mL). The combined organic extracts were dried

($MgSO_4$), filtered and the volatiles were removed under reduced pressure. Column chromatography on silica (0→100% ethyl acetate in petroleum ether) yielded the titled compound as a colorless, amorphous solid (450 mg, 2.09 mmol, 83%).

***R*f** 0.13 (20% ethyl acetate in petroleum ether); **^{1}H NMR (400 MHz, $CDCl_3$)** δ 8.32 (2H, d, *J* 4.7 Hz, H4 and H6), 7.32-7.22 (2H, m, H4' and H5'), 7.04-6.97 (2H, m, H3' and H6'), 6.52 (1H, t, *J* 4.7 Hz, H5), 3.77 (3H, s, OC*H*3), 3.41 (3H, s, NC*H*3); **^{13}C NMR (100 MHz, $CDCl_3$)** δ 162.3 (Q), 157.6, 155.3 (Q), 134.0 (Q), 129.2, 128.1, 121.1, 112.3, 110.2, 55.5, 37.8; **IR ν_{max}/cm^{-1} (neat)** 2939, 1579, 1549, 1484, 1395; **HRMS (ESI)** found *m/z* 216.11312 $[M+H]^+$, $C_{12}H_{14}N_3O$ requires *m/z* 216.11314.

4-(1-Methyl-1*H*-pyrazol-4-yl)pyrimidine (S4)

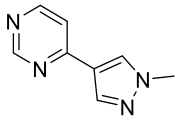

Sodium hydride (96 mg, 60 wt% in mineral oil, 2.40 mmol, 1.2 eq.) was added portionwise to a solution of 4-(1*H*-pyrazol-4-yl)pyrimidine (292 mg, 2.0 mmol, 1.0 eq.) in anhydrous dimethylformamide (10 mL) at 0 °C. The mixture was warmed to room temperature and stirred for 1.5 hours, then methyl iodide (0.15 mL, 2.4 mmol, 1.2 eq.) was added dropwise. The reaction was stirred for a further 24 hours, then quenched with water (10 mL), extracted with ethyl acetate (3 x 10 mL), and the combined organic phases washed with water (2 x 10 mL) and brine (10 mL). The organic phase was dried ($MgSO_4$) and concentrated *in vacuo* to give **S4** as a pale yellow solid (89 mg, 0.556 mmol, 28%).

***R*f** 0.12 (100% ethyl acetate); **mp** 83-85 °C (CH_2Cl_2); **^{1}H NMR (400 MHz, $CDCl_3$)** δ 9.02 (1H, s, H1), 8.54 (1H, d, *J* 5.2 Hz, H4), 7.98 (1H, s, H5'), 7.96 (1H, s, H3'), 7.31 (1H, d, *J* 5.2 Hz, H5), 3.89 (3H, s, NC*H*3); **^{13}C NMR (100 MHz, $CDCl_3$)** δ 159.0, 158.6 (Q), 156.9, 138.3, 130.2, 121.3 (Q), 116.1, 39.3; **IR ν_{max}/cm^{-1} (neat)** 3110, 3037, 1587, 1532, 1400, 1228; **HRMS (ESI)** found *m/z* 161.08211 $[M+H]^+$, $C_8H_9N_4$ requires *m/z* 161.08217.

***N*-(6,7-Bis(2-methoxyethoxy)quinazolin-4-yl)-*N*-(3-ethynylphenyl)acetamide (S5)**

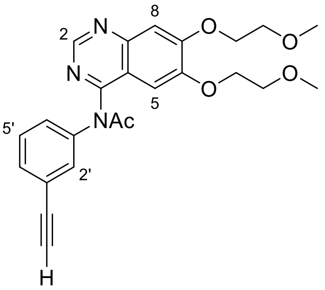

Acetic anhydride (0.30 mL, 3.05 mmol, 6.0 eq.) was added to a solution of erlotinib (200 mg, 0.508 mmol, 1.0 eq.), triethylamine (0.64 mL, 4.58 mmol, 9.0 eq.) and 4-dimethylaminopyridine (9 mg, 0.075 mmol, 0.15 eq.) in chloroform (1.7 mL) and heated to 60 °C for 5 days. The mixture was cooled, quenched with $NaHCO_3$, extracted with ethyl acetate (3 x 5 mL), dried ($MgSO_4$) and concentrated *in vacuo*. The crude residue was purified *via* flash column chromatography (100% ethyl acetate) to give **S5** as a yellow oil (151 mg, 0.347 mmol, 68%).

***R*$_f$** 0.10 (100% ethyl acetate); **^{1}H NMR (400 MHz, $CDCl_3$)** δ 9.07 (1H, s, H2), 7.46-7.44 (1H, m, H2'), 7.40-7.37 (2H, m, H4' and H6'), 7.35 (1H, s, H8), 7.34-7.31 (1H, m, H5'), 7.15 (1H, s, H5), 4.33-4.31 (2H, m, $ArOCH_2CH_2OCH_3$), 4.18-4.15 (2H, m, $ArOCH_2CH_2OCH_3$), 3.88-3.85 (2H, m, $ArOCH_2CH_2OCH_3$), 3.82-3.78 (2H, m, $ArOCH_2CH_2OCH_3$), 3.47 (3H, s, $ArOCH_2CH_2OCH_3$), 3.46 (3H, s, $ArOCH_2CH_2OCH_3$), 3.07 (1H, s, CC*H*), 2.15 (3H, s, $NCOCH_3$); **^{13}C NMR (100 MHz, $CDCl_3$)** δ 170.6 (Q), 158.9 (Q), 156.2 (Q), 153.6, 151.2 (Q), 150.9 (Q), 141.0 (Q), 131.1, 130.4, 129.4, 127.6, 123.5 (Q), 117.3 (Q), 107.9, 103.1, 82.4 (Q), 78.4, 70.6, 70.3, 68.9, 68.8, 59.4, 59.4, 23.8; **IR ν_{max}/ cm^{-1} (neat)** 3254, 2930, 2888, 1686, 1561, 1498, 1435, 1363, 1233, 1125; **HRMS (ESI)** found *m/z* 436.18689 $[M+H]^+$, $C_{24}H_{26}O_5N_3$ requires *m/z* 436.18670.

Cyanation of Heterocyclic Compounds

General Procedure A. Triflic anhydride (1.2 eq.) was added dropwise to a solution of starting material (1.0 eq.) in anhydrous $CHCl_3$ (0.1 M wrt substrate) in a vial with a subaseal at room temperature under argon. The resulting solution was stirred for one hour, then trimethylsilyl cyanide (5.0 eq.) was added. The subaseal was quickly swapped for a screw cap and the mixture stirred at 60 °C for 3 h. The reaction was removed from the heating source, and *N*-methylmorpholine (1.3 eq.) added quickly before the vial was recapped. The mixture was then stirred at 60 °C for a further 17 h, before cooling to room temperature, and quenching with sat. $NaHCO_3$ solution. The phases were separated, and the organic phase was extracted twice with CH_2Cl_2. The combined organic phase was dried ($MgSO_4$ or Na_2SO_4 as appropriate) and concentrated *in vacuo*. The crude mixture was purified *via* flash column chromatography.

2-Phenylisonicotinonitrile (5) and 6-phenylpicolinonitrile (5')

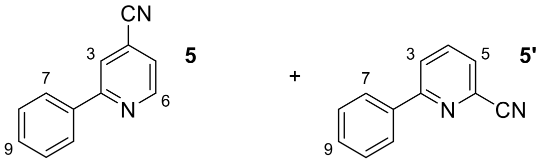

According to General Procedure A, triflic anhydride (0.13 mL, 0.768 mmol, 1.2 eq.), 2-phenylpyridine (100 mg, 0.64 mmol, 1.0 eq.), trimethylsilyl cyanide (0.40 mL, 3.20 mmol, 5.0 eq.) and *N*-methylmorpholine (91 μL, 0.83 mmol, 1.3 eq.) gave, following flash column chromatography (0→10% ethyl acetate in petroleum ether), separable isomers **5** as a colorless oil (57 mg, 0.316 mmol, 49%) and **5'** as a yellow oil (36 mg, 0.199 mmol, 31%).

5: $\boldsymbol{R_f}$ 0.31 (20% ethyl acetate in petroleum ether); **^{1}H NMR (400 MHz, $CDCl_3$)** δ 8.85 (1H, dd, *J* 5.0 and 1.0 Hz, H6), 8.01-7.96 (2H, m, H7), 7.94 (1H, dd, *J* 1.5 and 1.0 Hz, H3), 7.54-7.47 (3H, m, H8 and H9), 7.44 (1H, dd, *J* 5.0 and 1.5 Hz, H3); **^{13}C NMR (100 MHz, $CDCl_3$)** δ 158.8 (Q), 150.6, 137.4 (Q), 130.3, 129.2, 127.1, 123.2, 122.1, 121.2 (Q), 116.8 (Q); **IR ν_{max}/cm^{-1} (neat)** 3063, 2237, 1594, 1545, 1471, 1445, 1391; **LRMS (ESI)** found *m/z* 181.1 $[M+H]^+$, $C_{12}H_9N_2$ requires *m/z* 181.1. The spectroscopic data were found to be in agreement with that reported by Shen and co-workers.[2]

5': $\boldsymbol{R_f}$ 0.20 (20% ethyl acetate in petroleum ether); **^{1}H NMR (400 MHz, $CDCl_3$)** δ 8.05-8.02 (2H, m, H7), 7.96 (1H, dd, *J* 8.2 and 1.1 Hz, H5), 7.89 (1H, t, *J* 8.2 Hz, H4), 7.63 (1H, dd, *J* 8.2 and 1.1 Hz, H3), 7.54-7.47 (3H, m, H8 and H9); **^{13}C NMR (100 MHz, $CDCl_3$)** δ 159.0 (Q), 137.7, 137.2 (Q), 133.9 (Q), 130.3, 129.0, 127.2, 126.6, 123.5, 117.4 (Q); **IR ν_{max}/cm^{-1} (neat)** 3061, 2924, 2232, 1583, 1558, 1445; **LRMS (ESI)** found *m/z* 181.1 $[M+H]^+$, $C_{12}H_9N_2$ requires *m/z* 181.1. The spectroscopic data were found to be in agreement with that reported by Fier and co-workers.[3]

3-Phenylisonicotinonitrile (6), 5-phenylpicolinonitrile (6') and 3-phenylpicolinonitrile (6'')

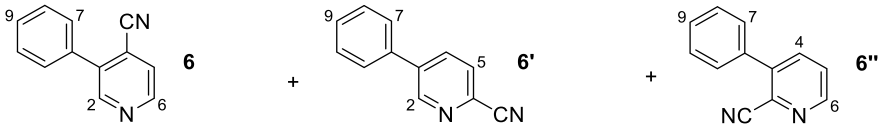

According to General Procedure A, triflic anhydride (0.14 mL, 0.84 mmol, 1.2 eq.), 3-phenylpyridine (109 mg, 0.70 mmol, 1.0 eq.), trimethylsilyl cyanide (0.44 mL, 3.50 mmol, 5.0 eq.) and *N*-methylmorpholine (100 μL, 0.91 mmol, 1.3 eq.) gave, following flash column chromatography (18% ethyl acetate in petroleum ether), separable isomers **6** as a colorless solid (79 mg, 0.438 mmol, 62%), **6'** as a colorless solid (21 mg, 0.117 mmol, 17%) and **6''** as a colorless solid (12 mg, 0.067 mmol, 10%).

6: $\boldsymbol{R_f}$ 0.27 (20% ethyl acetate in petroleum ether); **mp** 97-99 °C (CH_2Cl_2; lit.[4] 85-87 °C); **^{1}H NMR (400 MHz, $CDCl_3$)** δ 8.85 (1H, s, H2), 8.73 (1H, d, *J* 5.5 Hz, H6), 7.61 (1H, d, *J* 5.5 Hz, H5), 7.59-7.48 (5H, m, H7, H8 and H9); **^{13}C NMR (100 MHz, $CDCl_3$)** δ 151.0, 148.7, 138.7 (Q), 134.5 (Q), 129.7, 129.3, 128.9, 126.1, 118.8 (Q), 116.4 (Q); **IR ν_{max}/cm^{-1} (neat)** 3098, 3044, 2234, 1583, 1479, 1447, 1402, 1204, 1079; **LRMS (ESI)** found *m/z* 181.0 $[M+H]^+$, $C_{12}H_9N_2$ requires *m/z* 181.1. The spectroscopic data were found to be in agreement with that reported by Huang and co-workers.[4]

6': $\boldsymbol{R_f}$ 0.24 (10% ethyl acetate in petroleum ether); **mp** 88-90 °C (CH_2Cl_2, lit.[5] 93-94 °C); **^{1}H NMR (500 MHz, $CDCl_3$)** δ 8.94 (1H, dd, *J* 2.3 and 0.7 Hz, H2), 8.00 (1H, dd, *J* 8.1 and 2.3 Hz, H4), 7.76 (1H, dd, *J* 8.1 and 0.7 Hz, H5), 7.61-7.57 (2H, m, H7), 7.54-7.46 (3H, m, H8 and H9); **^{13}C NMR (125 MHz, $CDCl_3$)** δ 149.7, 139.9 (Q), 136.0 (Q), 134.9, 132.3 (Q), 129.5, 129.5, 128.6, 127.3, 117.4 (Q); **IR ν_{max}/cm^{-1} (neat)** 3062, 2233, 1584, 1468, 1368, 1029; **HRMS (ESI)** found *m/z* 181.07610 $[M+H]^+$, $C_{12}H_9N_2$ requires *m/z* 181.07602. The spectroscopic data were found to be in agreement with that reported by Sakamoto and co-workers.[5]

6'': $\boldsymbol{R_f}$ 0.10 (10% ethyl acetate in petroleum ether); **mp** 124-126 °C (CH_2Cl_2; lit.[5] 125-126 °C); **^{1}H NMR (500 MHz, $CDCl_3$)** δ 8.71 (1H, dd, *J* 4.5 and 1.6 Hz, H6), 7.87 (1H, dd, *J* 8.2 and 1.6 Hz, H4), 7.61-7.48 (6H, m, H5, H7, H8 and H9; **^{13}C NMR (125 MHz, $CDCl_3$)** δ 149.4, 142.1 (Q), 137.6, 135.3 (Q), 132.2 (Q), 129.5, 129.1, 128.8, 126.6, 117.0 (Q); **IR ν_{max}/cm^{-1} (neat)** 3051, 2924, 2236, 1577, 1554, 1448, 1411, 1122, 1005; **LRMS (ESI)** found *m/z* 181.0 $[M+H]^+$, $C_{12}H_9N_2$ requires *m/z* 181.1. The spectroscopic data were found to be in agreement with that reported by Sakamoto and co-workers.[5]

3-Methylisonicotinonitrile (7) and 5-methylpicolinonitrile (7')

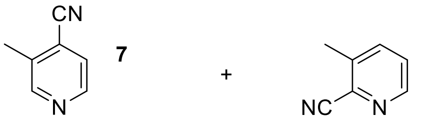

According to General Procedure A, triflic anhydride (0.14 mL, 0.84 mmol, 1.2 eq.), 3-methylpyridine (65 mg, 0.70 mmol, 1.0 eq.), trimethylsilyl cyanide (0.44 mL, 3.50 mmol, 5.0 eq.) and *N*-methylmorpholine (100 μL, 0.91 mmol, 1.3 eq.) gave, following flash column chromatography (20 % ethyl acetate in petroleum ether), separable isomer **7** as a colorless solid (50 mg, 0.423 mmol, 60%) and **7'** as a colorless solid which could not be fully separated from **7** (3.5 mg, 0.030 mmol, 4%).

7: $\boldsymbol{R}_f$ 0.18 (20% ethyl acetate in petroleum ether); **mp** 48-49 °C (CH_2Cl_2; lit.[6] 51 °C); **^{1}H NMR (400 MHz, $CDCl_3$)** δ 8.64 (1H, s, H2), 8.56 (1H, d, *J* 5.1 Hz, H6), 7.45 (1H, d, *J* 5.1 Hz, H5), 2.51 (3H, s, $ArCH_3$); **^{13}C NMR (100 MHz, $CDCl_3$)** δ 151.6, 147.7, 135.5 (Q), 125.0, 120.6 (Q), 115.7 (Q), 17.4; **IR ν_{max}/cm^{-1} (neat)** 3046, 2921, 2234, 1590, 1485, 1405, 1193, 1056; **HRMS (ESI)** found *m/z* 119.06049 $[M+H]^+$, $C_7H_7N_2$ requires *m/z* 119.06037. The spectroscopic data were found to be in agreement with that reported by Steinhauer and co-workers.[6]

7': $\boldsymbol{R}_f$ 0.18 (20% ethyl acetate in petroleum ether); **^{1}H NMR (500 MHz, $CDCl_3$)** δ 8.55 (1H, dd, *J* 4.7 and 1.1 Hz, H6), 7.68-7.66 (1H, m, H4), 7.42 (1H, dd, *J* 7.6 and 4.7 Hz, H5), 2.58 (3H, s, $ArCH_3$); **^{13}C NMR (125 MHz, $CDCl_3$)** δ 148.5, 138.4 (Q), 138.0, 134.2 (Q), 126.6, 116.3 (Q), 18.8; **HRMS (ESI)** found *m/z* 119.06062 $[M+H]^+$, $C_7H_7N_2$ requires *m/z* 119.06037. The spectroscopic data were found to be in agreement with that reported by Katritzky and co-workers.[7]

4-(Trifluoromethyl)picolinonitrile (8)

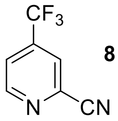

According to General Procedure A, triflic anhydride (0.14 mL, 0.84 mmol, 1.2 eq.), 4-(trifluoromethyl)pyridine (103 mg, 0.70 mmol, 1.0 eq.), trimethylsilyl cyanide (0.44 mL, 3.50 mmol, 5.0 eq.) and *N*-methylmorpholine (100 μL, 0.91 mmol, 1.3 eq.) gave, following flash column chromatography (10→25% ethyl acetate in petroleum ether), single isomer **8** as a pale yellow oil (87 mg, 0.503 mmol, 72%).

$\boldsymbol{R}_f$ 0.33 (20% ethyl acetate in petroleum ether); **^{1}H NMR (400 MHz, $CDCl_3$)** δ 8.94 (1H, d, *J* 5.1 Hz, H6), 7.92-7.91 (1H, m, H3), 7.78-7.76 (1H, m, H5); **^{13}C NMR (100 MHz, $CDCl_3$)** δ 152.3, 139.8 (Q, q, J_{FC} 35.5 Hz), 135.2 (Q), 124.2 (q, J_{FC} 3.2 Hz), 122.6 (q, J_{FC} 3.2 Hz), 121.8 (Q, q, J_{FC} 274.8 Hz), 116.1 (Q);); **^{19}F NMR (377 MHz, $CDCl_3$)** δ -65.0; **IR ν_{max}/cm^{-1} (neat)** 3075, 2243, 1401, 1327, 1174, 1140, 1083; **HRMS (ESI)** found *m/z* 173.07871 $[M+H]^+$, $C_7H_4F_3N_2$ requires *m/z* 173.03211.

The spectroscopic data were found to be in agreement with that reported by Changyou and co-workers.[8]

3-(Trifluoromethyl)isonicotinonitrile (9) and 5-(trifluoromethyl)picolinonitrile (9')

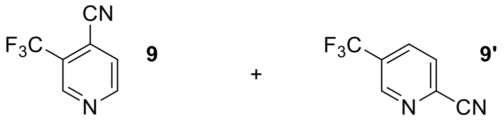

According to General Procedure A, triflic anhydride (140 μL, 0.84 mmol, 1.2 eq.), 3-(trifluoromethyl)pyridine (103 mg, 0.70 mmol, 1.0 eq.), TMSCN (0.44 mL, 3.5 mmol, 5.0 eq.) and *N*-methylmorpholine (100 μL, 0.91 mmol, 1.3 eq.) gave, following flash column chromatography (0→50% ethyl acetate in petroleum ether) separable isomers **9** as a colorless oil (46 mg, 0.260 mmol, 37%) and **9'** as a colorless oil (5 mg, 0.03 mmol, 4%).

9: $\boldsymbol{R}_{\mathbf{f}}$ 0.30 (20% ethyl acetate in petroleum ether); **^{1}H NMR (400 MHz, $CDCl_3$)** δ 9.11 (1H, s, H2), 9.04 (1H, d, *J* 5.0 Hz, H5), 7.74 (1H, d *J* 5.0 Hz, H4); **^{13}C NMR (100 MHz, $CDCl_3$)** δ 154.0, 147.8 (q, J_{FC} 5 Hz), 126.9, 126.7 (Q, q, *J* 33.0 Hz), 121.8 (Q, q, J_{FC} 274 Hz), 118.5 (Q, q, J_{FC} 5 Hz), 113.2 (Q); **^{19}F NMR (377 MHz, $CDCl_3$)** δ -61.7; **IR ν_{max}/cm^{-1} (neat);** 3282, 3108, 2959, 2924, 2533, 2244, 1649, 1363, 1284, 1137, 1071, 1016; **HRMS (EI)** found *m/z* 172.0248 $[M]^+$, $C_7H_3N_2F_3$ requires *m/z* 172.0243. The spectroscopic data were found to be in agreement with that reported by Fennewald and co-workers.[10]

9': $\boldsymbol{R}_{\mathbf{f}}$ 0.39 (20% ethyl acetate in petroleum ether); **^{1}H NMR (400 MHz, $CDCl_3$)** δ 9.00 (1H, d, *J* 0.6 Hz, H6), 8.12 (1H, ddd, *J* 8.1, 2.2, and 0.6 Hz, H4), 7.87 (1H, d, *J* 8.1 Hz, H3); **^{13}C NMR (100 MHz, $CDCl_3$)** δ 148.1 (q, J_{FC} 4 Hz), 137.1 (Q), 135.0 (q, J_{FC} 4 Hz), 129.5 (Q, q, J_{FC} 34 Hz), 128.3, 122.5 (Q, q, J_{FC} 274 Hz), 116.2 (Q); **^{19}F NMR (377 MHz, $CDCl_3$)** δ -63.0; **IR ν_{max}/cm^{-1} (neat)**; 3108, 3070, 2247, 1596, 1573, 1325, 1129, 1138, 1073, 1041; **HRMS (EI)** found *m/z* 172.0248 $[M]^+$, $C_7H_3N_2F_3$ requires *m/z* 172.0243. The spectroscopic data were found to be in agreement with that reported by Maeda and co-workers.[9]

Pyridine-2,4-dicarbonitrile (10)

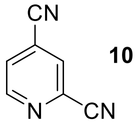

According to General Procedure A, triflic anhydride (0.14 mL, 0.84 mmol, 1.2 eq.), 4-cyanopyridine (73 mg, 0.70 mmol, 1.0 eq.), trimethylsilyl cyanide (0.44 mL, 3.50 mmol, 5.0 eq.) and *N*-

methylmorpholine (100 μL, 0.91 mmol, 1.3 eq.) gave, following flash column chromatography (20% ethyl acetate in petroleum ether), single isomer **10** as a pale yellow solid (84 mg, 0.651 mmol, 93%).

***R*f** 0.26 (20% ethyl acetate in petroleum ether); **mp** 89-90 °C (CH_2Cl_2; lit.[5] 89-80 °C); **^{1}H NMR (400 MHz, $CDCl_3$)** δ 8.94 (1H, dd, *J* 4.9 and 0.7 Hz, H6), 7.93 (1H, dd, *J* 1.5 and 0.7 Hz, H3), 7.79 (1H, dd, *J* 4.9 and 1.5 Hz, H5); **^{13}C NMR (100 MHz, $CDCl_3$)** δ 152.2, 135.3 (Q), 129.6, 128.5, 122.1 (Q), 115.6 (Q), 114.6 (Q); **IR ν_{max}/cm^{-1} (neat)** 3015, 2986, 2244, 1591, 1547, 1392, 1290, 1217, 1107; **LRMS (ESI)** found *m/z* 130.0 $[M+H]^+$, $C_7H_4N_3$ requires *m/z* 130.0. The spectroscopic data were found to be in agreement with that reported by Sakamoto and co-workers.[5]

Methyl 2-cyanoisonicotinate (11)

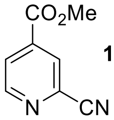

11

According to General Procedure A, triflic anhydride (0.14 mL, 0.84 mmol, 1.2 eq.), methyl isonicotinate (96 mg, 0.70 mmol, 1.0 eq.), trimethylsilyl cyanide (0.44 mL, 3.50 mmol, 5.0 eq.) and *N*-methylmorpholine (100 μL, 0.91 mmol, 1.3 eq.) gave, following flash column chromatography (20% ethyl acetate in petroleum ether), single isomer **11** as a colorless solid (104 mg, 0.641 mmol, 92%).

***R*f** 0.15 (20% ethyl acetate in petroleum ether); **mp** 102-103 °C (CH_2Cl_2; lit.[11] 100-103 °C); **^{1}H NMR (400 MHz, $CDCl_3$)** δ 8.87 (1H, dd, *J* 5.1 and 1.0 Hz, H6), 8.22 (1H, dd, *J* 1.5 and 1.0 Hz, H3), 8.06 (1H, dd, *J* 5.1 and 1.5 Hz, H5), 3.98 (3H, s, CO_2CH_3); **^{13}C NMR (100 MHz, $CDCl_3$)** δ 163.7 (Q), 152.1, 138.7 (Q), 134.7 (Q), 127.7, 126.2, 116.6 (Q), 53.4; **IR ν_{max}/cm^{-1} (neat)** 3092, 3073, 2963, 2249, 1724, 1398, 1303, 1212; **HRMS (ESI)** found *m/z* 163.05045 $[M+H]^+$, $C_8H_7O_2N_2$ requires *m/z* 163.05020. The spectroscopic data were found to be in agreement with that reported by Sheridan and co-workers.[11]

Methyl 4-cyanonicotinate (12), methyl 6-cyanonicotinate (12’) and methyl 2-cyanonicotinate (12’’)

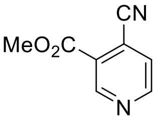

12 +

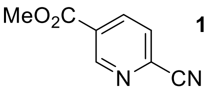

12' +

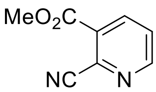

12''

According to General Procedure A, triflic anhydride (0.14 mL, 0.84 mmol, 1.2 eq.), methyl nicotinate (96 mg, 0.70 mmol, 1.0 eq.), trimethylsilyl cyanide (0.44 mL, 3.50 mmol, 5.0 eq.) and *N*-methylmorpholine (100 μL, 0.91 mmol, 1.3 eq.) gave, following flash column chromatography

(10→40% ethyl acetate in petroleum ether), inseparable isomers **12** and **12’’** as a colorless solid (92 mg, 4:1 **12**:**12’’**, 0.568 mmol, 81%), and separable isomer **13’** as a colorless solid (17 mg, 0.105 mmol, 15%).

12: ***R*f** 0.17 (40% ethyl acetate in petroleum ether); **^{1}H NMR (400 MHz, $CDCl_3$)** δ 9.35 (1H, s, H2), 8.93 (1H, d, *J* 5.2 Hz, H6), 7.68 (1H, d, *J* 5.2 Hz, H5), 4.02 (H, s, CO_2CH_3); **^{13}C NMR (100 MHz, $CDCl_3$)** δ 163.2 (Q), 153.8, 152.2, 127.7, 126.0 (Q), 120.9 (Q), 115.2 (Q), 53.2; **IR ν_{max}/cm^{-1} (neat)** 3009, 2958, 2238, 1731, 1582, 1556, 1437, 1407, 1301, 1284; **HRMS (ESI)** found *m/z* 163.05025 $[M+H]^+$, $C_8H_7O_2N_2$ requires *m/z* 163.05020.

12’: ***R*f** 0.33 (20% ethyl acetate in petroleum ether); **mp** 80-82 °C (CH_2Cl_2; lit.[12] 86-87 °C); **^{1}H NMR (400 MHz, $CDCl_3$)** δ 9.27 (1H, s, H2), 8.44 (1H, d, *J* 8.2 Hz, H4), 7.80 (1H, d, *J* 8.2 Hz, H5), 4.00 (3H, s, CO_2CH_3); **^{13}C NMR (100 MHz, $CDCl_3$)** δ 164.1 (Q), 151.8, 138.2, 136.9 (Q), 128.5 (Q), 128.1, 116.5 (Q), 53.1; **IR ν_{max}/cm^{-1} (neat)** 3068, 2987, 2249, 1726, 1588, 1438, 1296, 1283, 1110; **HRMS (ESI)** found *m/z* 163.05034 $[M+H]^+$, $C_8H_7O_2N_2$ requires *m/z* 163.05020. The spectroscopic data were found to be in agreement with that reported by Fife.[12]

12’’: ***R*f** 0.17 (20% ethyl acetate in petroleum ether); **^{1}H NMR (400 MHz, $CDCl_3$)** δ 8.84 (1H, dd, *J* 4.7 and 1.6 Hz, H6), 8.41 (1H, dd, *J* 8.1 and 1.6 Hz H4), 7.62 (1H, dd, *J* 8.1 and 4.7 Hz, H5), 4.01 (3H, s, CO_2CH_3); **^{13}C NMR (100 MHz, $CDCl_3$)** δ 163.3 (Q), 153.5, 138.7, 134.6 (Q), 129.7 (Q), 126.4, 116.0 (Q), 53.3; **IR ν_{max}/cm^{-1} (neat)** 3009, 2958, 2238, 1731, 1582, 1556, 1437, 1407, 1301, 1284; **HRMS (ESI)** found *m/z* 163.05025 $[M+H]^+$, $C_8H_7O_2N_2$ requires *m/z* 163.05020. The spectroscopic data were found to be in agreement with that reported by Ovdiichuk and co-workers.[13]

4-Cyanopyridin-3-yl acetate (13), 6-cyanopyridin-3-yl acetate (13’) and 2-cyanopyridin-3-yl acetate (13’’)

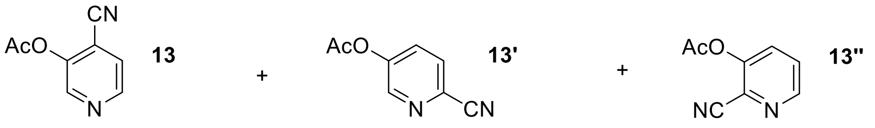

According to General Procedure A, triflic anhydride (0.14 mL, 0.84 mmol, 1.2 eq.), pyridin-3-yl acetate (96 mg, 0.70 mmol, 1.0 eq.), trimethylsilyl cyanide (0.44 mL, 3.50 mmol, 5.0 eq.) and *N*-methylmorpholine (100 μL, 0.91 mmol, 1.3 eq.) gave, following flash column chromatography (30% ethyl acetate in petroleum ether), inseparable isomers **13**, **13’** and **13’’** as a pale yellow oil (69 mg, 51:16:33 0.425 mmol, 61%).

13: ***R*f** 0.24 (40% ethyl acetate in petroleum ether); **^{1}H NMR (400 MHz, $CDCl_3$)** δ 8.64 (1H, s, H2), 8.61 (1H, d, *J* 4.9 Hz, H6), 7.55 (1H, d, *J* 4.9 Hz, H5), 2.39 (3H, s, $ArOCOCH_3$); **^{13}C NMR (100 MHz, $CDCl_3$)** δ 167.8 (Q), 147.4, 147.2 (Q), 145.4, 125.7, 114.8 (Q), 113.0 (Q), 20.6; **IR ν_{max}/cm^{-1}**

(neat) 3071, 2240, 1774, 1371, 1176, 1148, 1009; **HRMS (ESI)** found *m/z* 163.05033 $[M+H]^+$, $C_8H_7O_2N_2$ requires *m/z* 163.05020.

13': ***R*$_f$** 0.24 (40% ethyl acetate in petroleum ether); **^{1}H NMR (400 MHz, $CDCl_3$)** δ 8.49 (1H, d, *J* 2.5 Hz, H2), 7.72 (1H, d, *J* 7.7 Hz, H4), 7.64 (1H, dd, *J* 7.7 and 2.5 Hz, H5), 2.33 (3H, s, ArCOCH_3) ; **^{13}C NMR (100 MHz, $CDCl_3$)** δ 168.0 (Q), 149.4 (Q), 145.1, 130.4 (Q), 130.0, 129.2, 116.8 (Q), 21.0.

13'': ***R*$_f$** 0.24 (40% ethyl acetate in petroleum ether); **^{1}H NMR (400 MHz, $CDCl_3$)** δ 8.55-8.52 (1H, m, H6), 7.69-7.66 (1H, m, H4), 7.57-7.53 (1H, m, H5), 2.39 (3H, s, ArCOCH_3); **^{13}C NMR (100 MHz, $CDCl_3$)** δ 167.8 (Q), 150.3 (Q), 148.0, 131.2, 127.9 (Q), 127.8, 114.1 (Q), 20.8.

3-Chloroisonicotinonitrile (14), 5-chloropicolinonitrile (14') and 3-chloropicolinonitrile (14'')

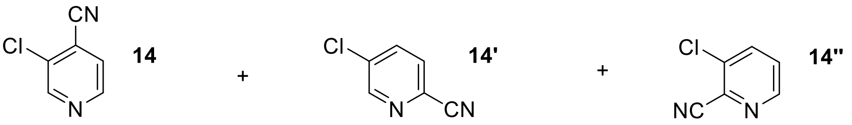

According to General Procedure A, triflic anhydride (0.14 mL, 0.84 mmol, 1.2 eq.), 3-chloropyridine (67 μL, 0.70 mmol, 1.0 eq.), trimethylsilyl cyanide (0.44 mL, 3.50 mmol, 5.0 eq.) and *N*-methylmorpholine (100 μL, 0.91 mmol, 1.3 eq.) gave, following flash column chromatography (0→70% ethyl acetate in petroleum ether), inseparable isomers **14** and **14"** as a colorless solid (56 mg, 0.400 mmol, 57%, **14**:**14"** 76:24) and separable **14'** as an off-white solid (6 mg, 0.04 mmol, 6%).

14: ***R*$_f$** 0.35 (20% ethyl acetate in petroleum ether); **^{1}H NMR (400 MHz, $CDCl_3$)** δ 8.81 (1H, d, *J* 0.7 Hz, H2), 8.67 (1H, d, *J* 5.0 Hz, H6), 7.56 (1H, dd, *J* 5.0 and 0.7 Hz, H5); **^{13}C NMR (100 MHz, $CDCl_3$)** δ 150.4, 148.1, 133.1 (Q), 126.4, 120.8 (Q), 113.7 (Q); **IR ν_{max}/cm^{-1} (neat)** 3050, 3018, 2239, 1573, 1473, 1422, 1402, 1208, 1098, 1038. The spectroscopic data were found to be in agreement with that reported by Rault and co-workers.[14]

14': ***R*$_f$** 0.50 (20% ethyl acetate in petroleum ether); **mp** 88-90 °C (CH_2Cl_2; lit.[5] 106-108 °C); **^{1}H NMR (400 MHz, $CDCl_3$)** δ 8.68 (1H, dd, *J* 2.4 and 0.8 Hz, H6), 7.84 (1H, dd, *J* 8.3 and 2.4 Hz, H4), 7.67 (1H, dd, *J* 8.3 and 0.8 Hz, H3); **^{13}C NMR (100 MHz, $CDCl_3$)** δ 150.5, 136.9, 136.1 (Q), 130.4 (Q), 129.0, 116.5 (Q); **IR ν_{max}/cm^{-1} (neat)** 3048, 2235, 1570, 1558, 1456, 1113, 1015. The spectroscopic data were found to be in agreement with that reported by Cai and co-workers.[15]

14": ***R*$_f$** 0.35 (20% ethyl acetate in petroleum ether); **^{1}H NMR (400 MHz, $CDCl_3$)** δ 8.61 (1H, dd, *J* 4.6 and 1.4 Hz, H6), 7.87 (1H, dd, *J* 8.3 and 1.4 Hz, H4), 7.50 (1H, dd, *J* 8.3 and 4.6 Hz, H5); **^{13}C NMR (100 MHz, $CDCl_3$)** δ 148.7, 137.6, 136.0 (Q), 133.1 (Q), 127.5 , 114.6 (Q). The spectroscopic data were found to be in agreement with that reported by Katritzky and co-workers.[7]

3-Bromoisonicotinonitrile (15), 5-bromopicolinonitrile (15') and 3-bromopicolinonitrile (15'')

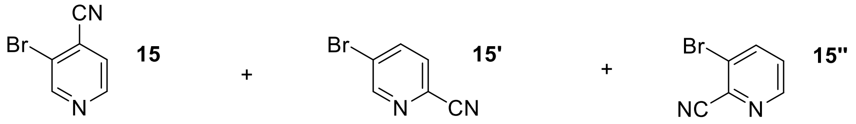

According to General Procedure A, triflic anhydride (0.14 mL, 0.84 mmol, 1.2 eq.), 3-bromopyridine (111 mg, 0.70 mmol, 1.0 eq.), trimethylsilyl cyanide (0.44 mL, 3.50 mmol, 5.0 eq.) and *N*-methylmorpholine (100 µL, 0.91 mmol, 1.3 eq.) gave, following flash column chromatography (0→10% ethyl acetate in petroleum ether), separable isomers **15** as a colorless solid (71 mg, 0.388 mmol, 55%), **15'** as a colorless solid (14 mg, 0.077 mmol, 11%) and **15''** as a colorless solid (17 mg, 0.091 mmol, 13%).

15: $\boldsymbol{R}_{\mathbf{f}}$ 0.14 (10% ethyl acetate in petroleum ether); **mp** 80-81 °C (CH_2Cl_2; lit.[16] 79-81°C); **^{1}H NMR (400 MHz, $CDCl_3$)** δ 8.95 (1H, s, H2), 8.74 (1H, d, *J* 4.8 Hz, H6), 7.57 (1H, d, *J* 4.8 Hz, H5); **^{13}C NMR (100 MHz, $CDCl_3$)** δ 152.8, 148.5, 126.9, 123.3 (Q), 122.2 (Q), 114.9 (Q); **IR ν_{max}/cm^{-1} (neat)** 3078, 3014, 2234, 1537, 1470, 1403, 1088, 1026. The spectroscopic data were found to be in agreement with that reported by Kumpan and co-workers.[16]

15': $\boldsymbol{R}_{\mathbf{f}}$ 0.22 (10% ethyl acetate in petroleum ether); **mp** 105 °C (sublimation; CH_2Cl_2); **^{1}H NMR (500 MHz, $CDCl_3$)** δ 8.82 (1H, d, *J* 2.1 Hz, H2), 8.02 (1H, dd, *J* 8.3 and 2.1 Hz, H4), 7.62 (1H, d, *J* 8.3 Hz, H5); **^{13}C NMR (125 MHz, $CDCl_3$)** δ 152.6, 139.8, 132.1 (Q), 129.2, 125.1 (Q), 116.5 (Q); **IR ν_{max}/cm^{-1} (neat)** 3048, 2235, 1562, 1553, 1454, 1092, 1009. The spectroscopic data were found to be in agreement with that reported by Song and co-workers.[17]

15'': $\boldsymbol{R}_{\mathbf{f}}$ 0.08 (10% ethyl acetate in petroleum ether); **mp** 98-100 °C (CH_2Cl_2); **^{1}H NMR (500 MHz, $CDCl_3$)** δ 8.68 (1H, dd, *J* 4.6 and 1.4 Hz, H6), 8.06 (1H, dd, *J* 8.2 and 1.4 Hz, H4), 7.44 (1H, dd, *J* 8.2 and 4.6 Hz, H5); **^{13}C NMR (125 MHz, $CDCl_3$)** δ 149.1, 140.6, 135.3 (Q), 127.6, 124.6 (Q), 115.6 (Q); **IR ν_{max}/cm^{-1} (neat)** 3046, 2236, 1557, 1420, 1250, 1059, 1027; **HRMS (ESI)** found *m/z* 182.95547 $[M+H]^+$, $C_6H_4N_2{}^{79}Br$ requires *m/z* 182.95524. The spectroscopic data were found to be in agreement with that reported by Katritzky and co-workers.[7]

4-(*Tert*-butyl)picolinonitrile (16)

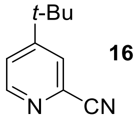

According to General Procedure A, triflic anhydride (0.14 mL, 0.84 mmol, 1.2 eq.), 4-(*tert*-butyl)pyridine (95 mg, 0.70 mmol, 1.0 eq.), trimethylsilyl cyanide (0.44 mL, 3.50 mmol, 5.0 eq.) and *N*-methylmorpholine (100 µL, 0.91 mmol, 1.3 eq.) gave, following flash column chromatography

(15→20% ethyl acetate in petroleum ether), single isomer **16** as a pale yellow oil (66 mg, 0.412 mmol, 59%).

***R*$_f$** 0.24 (10% ethyl acetate in petroleum ether); **^{1}H NMR (400 MHz, $CDCl_3$)** δ 8.58 (1H, d, *J* 5.2 Hz, H6), 7.67 (1H, dd, *J* 1.6 Hz, H3), 7.48 (1H, dd, *J* 5.2 and 1.6 Hz, H5), 1.34 (9H, s, $C(CH_3)_3$); **^{13}C NMR (100 MHz, $CDCl_3$)** δ 161.8 (Q), 151.0, 134.2 (Q), 126.0, 124.1, 117.7 (Q), 35.4 (Q), 30.3; **IR ν_{max}/cm^{-1} (neat)** 2965, 2870, 2237, 1592, 1478, 1397, 1202; **LRMS (ESI)** found *m/z* 161.2 $[M+H]^+$, $C_{10}H_{13}N_2$ requires *m/z* 161.1. The spectroscopic data were found to be in agreement with that reported by Ko and co-workers.[18]

6,7-Dihydro-5*H*-cyclopenta[*b*]pyridine-4-carbonitrile (17)

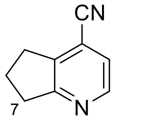

17

According to General Procedure A, triflic anhydride (0.14 mL, 0.84 mmol, 1.2 eq.), 6,7-dihydro-5*H*-cyclopenta[*b*]pyridine (83 mg, 0.70 mmol, 1.0 eq.), trimethylsilyl cyanide (0.44 mL, 3.50 mmol, 5.0 eq.) and *N*-methylmorpholine (100 µL, 0.91 mmol, 1.3 eq.) gave, following flash column chromatography (0→30% isopropanol in petroleum ether), single isomer **17** as a brown oil (40 mg, 0.28 mmol, 40%).

***R*$_f$** 0.33 (10% isopropanol in petroleum ether); **^{1}H NMR (400 MHz, $CDCl_3$)** δ 8.47 (1H, d, *J* 5.2 Hz, H6), 7.23 (1H, d, *J* 5.2 Hz, H5), 3.15 (2H, t, *J* 7.7 Hz, H7 or H9), 3.12 (2H, t, *J* 7.7 Hz, H7 or H9), 2.22 (2H, p, *J* 7.7 Hz, H8); **^{13}C NMR (100 MHz, $CDCl_3$)** δ 167.7 (Q), 148.2, 139.8 (Q), 122.0, 116.6 (Q), 115.9 (Q), 34.5, 30.4, 22.4; **IR ν_{max}/cm^{-1} (neat)** 2958, 2236, 1584, 1391, 1230, 1203, 835; **HRMS (ESI)** found *m/z* 145.0761 $[M+H]^+$, $C_9H_9N_2$ requires *m/z* 145.0760.

2-Isopropylisonicotinonitrile (18) and 6-isopropylpicolinonitrile (18')

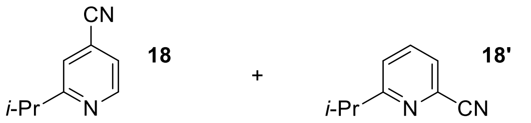

According to General Procedure A, triflic anhydride (0.14 mL, 0.84 mmol, 1.2 eq.), 2-isopropylpyridine (85 mg, 0.70 mmol, 1.0 eq.), trimethylsilyl cyanide (0.44 mL, 3.50 mmol, 5.0 eq.) and *N*-methylmorpholine (100 µL, 0.91 mmol, 1.3 eq.) gave, following flash column chromatography (0→10% ethyl acetate in petroleum ether), separable isomers **18** as an orange oil (42 mg, 0.287 mmol, 41%) and **18'** as a yellow oil (17 mg, 0.116 mmol, 17%).

18: ***R*****f** 0.44 (20% ethyl acetate in petroleum ether); **^{1}H NMR (400 MHz, CDCl$_3$)** δ 8.69 (1H, dd, *J* 4.9 and 0.8 Hz, H6), 7.38 (1H, dd, *J* 1.6 and 0.8 Hz, H3), 7.31 (1H, dd, *J* 4.9 and 1.6 Hz, H5), 3.10 (1H, sept, *J* 7.0 Hz, ArC*H*(CH$_3$)$_2$), 1.29 (6H, d, *J* 7.0 Hz, ArCH(C*H*$_3$)$_2$); **^{13}C NMR (100 MHz, CDCl$_3$)** δ 168.9 (Q), 150.1, 122.6, 122.5, 120.6 (Q), 116.9 (Q), 36.4, 22.3; **IR ν_{max}/cm^{-1} (neat)** 2967, 2931, 2237, 1593, 1551, 1475, 1398, 1206; **HRMS (ESI)** found *m/z* 147.09165 [M+H]$^+$, $C_9H_{11}N_2$ requires *m/z* 147.09167. The spectroscopic data were found to be in agreement with that reported by Mai and co-workers.[19]

18': ***R*****f** 0.53 (20% ethyl acetate in petroleum ether); **^{1}H NMR (400 MHz, CDCl$_3$)** δ 7.73 (1H, t, *J* 7.9 Hz, H4), 7.51 (1H, dd, *J* 7.9 and 1.0 Hz, H3), 7.39 (1H, dd, *J* 7.9 and 1.0 Hz, H5), 3.10 (1H, sept, *J* 6.9 Hz, ArC*H*(CH$_3$)$_2$), 1.30 (6H, d, *J* 6.9 Hz, ArCH(C*H*$_3$)$_2$); **^{13}C NMR (100 MHz, CDCl$_3$)** δ 169.4 (Q), 137.2, 133.1 (Q), 125.9, 124.4, 117.6 (Q), 36.4, 22.3; **IR ν_{max}/cm^{-1} (neat)** 2968, 2874, 2236, 1588, 1470, 1445; **LRMS (ESI)** found *m/z* 147.0 [M+H]$^+$, $C_9H_{11}N_2$ requires *m/z* 147.1. The spectroscopic data were found to be in agreement with that reported by Nienkemper and co-workers.[20]

3-Hexylisonicotinonitrile (19)

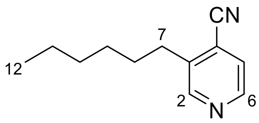

19

According to General Procedure A, triflic anhydride (0.14 mL, 0.84 mmol, 1.2 eq.), 3-hexylpyridine (114 mg, 0.70 mmol, 1.0 eq.), trimethylsilyl cyanide (0.44 mL, 3.50 mmol, 5.0 eq.) and *N*-methylmorpholine (100 µL, 0.91 mmol, 1.3 eq.) gave, following flash column chromatography (5→15% ethyl acetate in petroleum ether), single isomer **19** as a pale yellow oil (85 mg, 0.451 mmol, 64%).

R**f** 0.18 (20% ethyl acetate in petroleum ether); **^{1}H NMR (400 MHz, CDCl$_3$)** δ 8.64 (1H, s, H2), 8.58 (1H, d, *J* 4.9 Hz, H6), 7.45 (1H, d, *J* 4.9 Hz, H5), 2.87-2.80 (2H, m, H7), 1,71-1.63 (2H, m, H8), 1.40-1.24 (6H, m, H9, H10 and H11), 0.89-0.84 (3H, m, H12); **^{13}C NMR (100 MHz, CDCl$_3$)** δ 151.3, 147.8, 140.1 (Q), 125.3, 120.1 (Q), 115.8 (Q), 31.9, 31.4, 30.7, 28.8, 22.5, 14.0; **IR ν_{max}/cm^{-1} (neat)** 2956, 2929, 2858, 2233, 1465, 1409; **HRMS (ESI)** found *m/z* 189.13870 [M+H]$^+$, $C_{12}H_{16}N_2$ requires *m/z* 189.13863.

Quinoline-2-carbonitrile (20) and quinoline-4-carbonitrile (20')

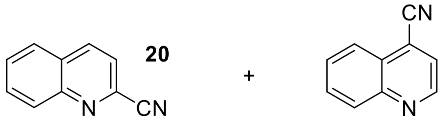

According to General Procedure A, triflic anhydride (0.14 mL, 0.84 mmol, 1.2 eq.), quinoline (90 mg, 0.70 mmol, 1.0 eq.), trimethylsilyl cyanide (0.44 mL, 3.50 mmol, 5.0 eq.) and *N*-methylmorpholine (100 μL, 0.91 mmol, 1.3 eq.) gave, following flash column chromatography (10→25% ethyl acetate in petroleum ether), separable isomers **20** as a colorless solid (71 mg, 0.461 mmol, 66%) and **20'** as a pale orange solid (21 mg, 0.136 mmol, 19%).

20: $\boldsymbol{R_f}$ 0.27 (20% ethyl acetate in petroleum ether); **mp** 94-95 °C (CH_2Cl_2; lit.[21] 89-91 °C); **^{1}H NMR (400 MHz, $CDCl_3$)** δ 8.29 (1H, d, *J* 8.6 Hz, H4), 8.13 (1H, d, *J* 9.2 Hz, H8), 7.88 (1H, d, *J* 7.8 Hz, H5), 7.84-7.80 (1H, m, H7), 7.71-7.66 (2H, m, H3 and H6); **^{13}C NMR (100 MHz, $CDCl_3$)** 148.2 (Q), 137.6, 133.6 (Q), 131.3, 129.9, 129.5, 128.7 (Q), 127.8, 123.3, 117.6 (Q); **IR ν_{max}/cm^{-1} (neat)** 3060, 2234, 1618, 1500, 1303, 1121; **LRMS (ESI)** found *m/z* 155.0 $[M+H]^+$, $C_{10}H_7N_2$ requires *m/z* 155.1. The spectroscopic data were found to be in agreement with that reported by Xie and co-workers.[21]

20': $\boldsymbol{R_f}$ 0.14 (20% ethyl acetate in petroleum ether); **mp** 100-101 °C (CH_2Cl_2; lit.[22] 106-107 °C); **^{1}H NMR (400 MHz, $CDCl_3$)** δ 9.03 (1H, d, *J* 4.4 Hz, H2), 8.22-8.17 (2H, m, H8 and H5), 7.88-7.84 (1H, m, H7), 7.78-7.72 (2H, m, H3 and H6); **^{13}C NMR (100 MHz, $CDCl_3$)** δ 149.5, 148.1 (Q), 131.2, 130.4, 129.3, 125.7 (Q), 125.0, 124.9, 118.9 (Q), 115.6 (Q); **IR ν_{max}/cm^{-1} (neat)** 3064, 3040, 2228, 1503, 1361, 1161; **LRMS (ESI)** found *m/z* 155.0 $[M+H]^+$, $C_{10}H_7N_2$ requires *m/z* 155.1. The spectroscopic data were found to be in agreement with that reported by Guo and co-workers.[23]

6-Methylquinoline-2-carbonitrile (21) and 6-methylquinoline-4-carbonitrile (21')

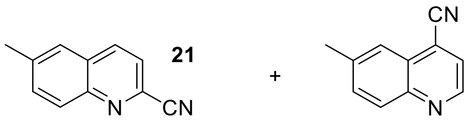

According to General Procedure A, triflic anhydride (0.14 mL, 0.84 mmol, 1.2 eq.), 6-methylquinoline (100 mg, 0.70 mmol, 1.0 eq.), trimethylsilyl cyanide (0.44 mL, 3.50 mmol, 5.0 eq.) and *N*-methylmorpholine (100 μL, 0.91 mmol, 1.3 eq.) gave, following flash column chromatography (0→40% ethyl acetate in petroleum ether), separable isomers **21** as a colorless, crystalline solid (94 mg, 0.56 mmol, 80%) and **21'** which was precipitated from ethanol as a colorless, amorphous solid (6 mg, 0.04 mmol, 5%).

21: $\boldsymbol{R_f}$ 0.28 (20% ethyl acetate in petroleum ether); **mp** 131-133 °C (CH_2Cl_2; lit.[23] 127-128 °C); **^{1}H NMR (400 MHz, $CDCl_3$)** δ 8.19 (1H, d, *J* 8.4 Hz, H4), 8.02 (1H, d, *J* 8.4 Hz, H3), 7.68-7.61 (3H, m, H5, H7 and H8), 2.58 (3H, s, CH_3); **^{13}C NMR (100 MHz, $CDCl_3$)** δ 146.7 (Q), 139.9 (Q), 136.5,

133.6, 132.4 (Q), 129.4, 128.6 (Q), 126.4, 123.2, 117.6 (Q), 21.8; **IR ν_{max}/cm^{-1} (neat)** 3657, 2980, 2230, 1382, 1222, 1138; **HRMS (ESI)** found *m/z* 169.07611 $[M+H]^+$, $C_{11}H_9N_2$ requires *m/z* 169.07602. The spectroscopic data were found to be in agreement with that reported by Guo and co-workers.[23]

21': *R*$_f$ 0.13 (20% ethyl acetate in petroleum ether); **^{1}H NMR (400 MHz, CDCl$_3$)** δ 8.97 (1H, d, *J* 4.3 Hz, H2), 8.10 (1H, d, *J* 8.6 Hz, H8), 7.97 (1H, s, H5), 7.74-7.66 (2H, m, H3 and H7), 2.63 (3H, s, C*H*$_3$); **^{13}C NMR (125 MHz, CDCl$_3$)** δ 148.4, 146.8 (Q), 139.9 (Q), 133.5, 130.0, 125.9 (Q), 124.7, 123.8, 117.8 (Q), 115.7 (Q), 21.8; **IR ν_{max}/cm^{-1} (neat)** 2980, 2232, 1619, 1502, 1379, 862, 823; **HRMS (ESI)** found *m/z* 169.07607 $[M+H]^+$, $C_{11}H_9N_2$ requires *m/z* 169.07602. The spectroscopic data were found to be in agreement with that reported by Yamaguchi and co-workers.[24]

7-Methylquinoline-2-carbonitrile (22) and 7-methylquinoline-4-carbonitrile (22')

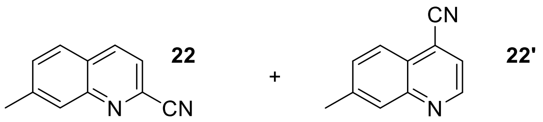

According to General Procedure A, triflic anhydride (0.14 mL, 0.84 mmol, 1.2 eq.), 7-methylquinoline (100 mg, 0.70 mmol, 1.0 eq.), trimethylsilyl cyanide (0.44 mL, 3.50 mmol, 5.0 eq.) and *N*-methylmorpholine (100 µL, 0.91 mmol, 1.3 eq.) gave, following flash column chromatography (0→40% ethyl acetate in petroleum ether), separable isomers **22** as a colorless, crystalline solid (74 mg, 0.44 mmol, 63%) and **22'** as a colorless, crystalline solid (23 mg, 0.14 mmol, 20%).

22: *R*$_f$ 0.25 (20% ethyl acetate in petroleum ether); **mp** 144-145 °C (CH_2Cl_2); **^{1}H NMR (400 MHz, CDCl$_3$)** δ 8.24 (1H, d, *J* 8.4 Hz, H4), 7.90 (1H, d, *J* 1.7 Hz, H8), 7.78 (1H, d, *J* 8.4 Hz, H5), 7.62 (1H, d, *J* 8.4 Hz, H3), 7.53 (1H, dd, *J* 8.4 and 1.7 Hz, H6), 2.59 (3H, s, C*H*$_3$); **^{13}C NMR (100 MHz, CDCl$_3$)** δ 148.3 (Q), 141.9 (Q), 137.0, 133.3 (Q), 131.7, 128.6, 127.3, 126.7 (Q), 122.5, 117.6 (Q), 21.9; **IR ν_{max}/cm^{-1} (neat)** 3658, 2980, 2232, 1382, 1152; **HRMS (ESI)** found *m/z* 167.07611 $[M+H]^+$, $C_{11}H_9N_2$ requires *m/z* 169.07602. The spectroscopic data were found to be in agreement with that reported by Sasaki and co-workers.[25]

22': *R*$_f$ 0.13 (20% ethyl acetate in petroleum ether); **mp** 102-103 °C (CH_2Cl_2; lit.[26] 102 °C); **^{1}H NMR (400 MHz, CDCl$_3$)** δ 8.99 (1H, d, *J* 4.4 Hz, H2), 8.08 (1H, d, *J* 8.4 Hz, H5), 7.97 (1H, d, *J* 1.6 Hz, H8), 7.66 (1H, d, *J* 4.4 Hz, H3), 7.59 (1H, dd, *J* 8.4 and 1.6 Hz, H6), 2.59 (3H, s, C*H*$_3$); **^{13}C NMR (100 MHz, CDCl$_3$)** δ 149.4, 148.3 (Q), 141.9 (Q), 131.4, 129.2, 124.5, 123.9, 123.8 (Q), 118.2 (Q), 115.7 (Q), 21.9; **IR ν_{max}/cm^{-1} (neat)** 3657, 2980, 2232, 1381, 1153, 954; **HRMS (ESI)** found *m/z* 169.07610 $[M+H]^+$, $C_{11}H_9N_2$ requires *m/z* 169.07602. The spectroscopic data were found to be in agreement with that reported by Yamaguchi and co-workers.[24]

Isoquinoline-1-carbonitrile (23)

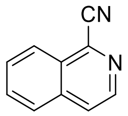

23

According to General Procedure A, triflic anhydride (0.14 mL, 0.84 mmol, 1.2 eq.), isoquinoline (90 mg, 0.70 mmol, 1.0 eq.), trimethylsilyl cyanide (0.44 mL, 3.50 mmol, 5.0 eq.) and *N*-methylmorpholine (100 μL, 0.91 mmol, 1.3 eq.) gave, following flash column chromatography (10→25% ethyl acetate in petroleum ether), single isomer **23** as a pale yellow solid (101 mg, 0.655 mmol, 94%).

***R*$_f$** 0.20 (20% ethyl acetate in petroleum ether); **mp** 88-89 °C (CH_2Cl_2; lit.[27] 87-89 °C); **^{1}H NMR (400 MHz, $CDCl_3$)** δ 8.67 (1H, d, *J* 5.5 Hz, H3), 8.39-8.35 (1H, m, H8), 7.97-7.95 (1H, m, H4), 7.92 (1H, d, *J* 5.4 Hz, H5), 7.90-7.80 (2H, m, H6 and H7); **^{13}C NMR (100 MHz, $CDCl_3$)** δ 143.3, 135.9 (Q), 134.8 (Q), 131.8, 129.9, 129.3 (Q), 127.3, 125.3, 124.5, 115.8 (Q); **IR ν_{max}/cm^{-1} (neat)** 3059, 2230, 1623, 1579, 1389, 1343; **LRMS (ESI)** found *m/z* 155.0 $[M+H]^+$, $C_{10}H_7N_2$ requires *m/z* 155.1. The spectroscopic data were found to be in agreement with that reported by Guo and co-workers.[23]

3-Bromoisoquinoline-1-carbonitrile (24)

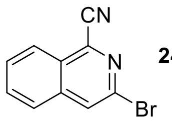

24

According to General Procedure A, triflic anhydride (0.14 mL, 0.84 mmol, 1.2 eq.), 3-bromoisoquinoline (146 mg, 0.70 mmol, 1.0 eq.), trimethylsilyl cyanide (0.44 mL, 3.50 mmol, 5.0 eq.) and *N*-methylmorpholine (100 μL, 0.91 mmol, 1.3 eq.) gave, following flash column chromatography (0→100% ethyl acetate in petroleum ether), single isomer **24** as a colorless, crystalline solid (121 mg, 0.52 mmol, 74%).

***R*$_f$** 0.34 (20% ethyl acetate in petroleum ether); **mp** 170 °C decomp. (CH_2Cl_2); **^{1}H NMR (400 MHz, $CDCl_3$)** δ 8.34-8.28 (1H, m, Ar*H*), 8.15 (1H, s, H4), 7.92-7.80 (3H, m, Ar*H*); **^{13}C NMR (100 MHz, $CDCl_3$)** δ 137.8 (Q), 134.8 (Q), 134.2 (Q), 132.7, 130.3, 128.4 (Q), 128.3, 126.4, 125.4, 114.6 (Q); **IR ν_{max}/cm^{-1} (neat)** 3658, 2980, 2231, 1383, 1148; **HRMS (ESI)** found *m/z* 232.97107 $[M+H]^+$, $C_{10}H_6N_2{}^{79}Br$ requires *m/z* 232.97089.

Pyridazine-3-carbonitrile (25)

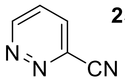

25

According to General Procedure A, triflic anhydride (0.14 mL, 0.84 mmol, 1.2 eq.), pyrazine (56 mg, 0.70 mmol, 1.0 eq.), trimethylsilyl cyanide (0.44 mL, 3.50 mmol, 5.0 eq.) and *N*-methylmorpholine (100 μL, 0.91 mmol, 1.3 eq.) gave, following flash column chromatography (10→40% ethyl acetate in petroleum ether), single isomer **25** as a yellow oil (70 mg, 0.666 mmol, 95%).

$\boldsymbol{R_f}$ 0.17 (40% ethyl acetate in petroleum ether); **^{1}H NMR (400 MHz, $CDCl_3$)** δ 9.36 (1H, dd, *J* 5.2 and 1.7 Hz, H6), 7.89 (1H, dd, *J* 8.7 and 1.7 Hz, H4), 7.71 (1H, dd, *J* 8.7 and 5.2 Hz, H5); **^{13}C NMR (100 MHz, $CDCl_3$)** δ 152.4, 140.0 (Q), 130.6, 126.7, 115.4 (Q); **IR ν_{max}/cm^{-1} (neat)** 3061, 2245, 1566, 1547, 1431, 1375, 1235; **HRMS (ESI)** found *m/z* 106.04032 $[M+H]^+$, $C_5H_4N_3$ requires *m/z* 106.03997. The spectroscopic data were found to be in agreement with that reported by Dostal and co-workers.[28]

6-Methylpyridazine-3-carbonitrile (26)

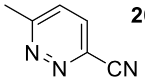

26

According to General Procedure A, triflic anhydride (0.14 mL, 0.84 mmol, 1.2 eq.), 3-methylpyrazine (66 mg, 0.70 mmol, 1.0 eq.), trimethylsilyl cyanide (0.44 mL, 3.50 mmol, 5.0 eq.) and *N*-methylmorpholine (100 μL, 0.91 mmol, 1.3 eq.) gave, following flash column chromatography (20→70% ethyl acetate in petroleum ether), single isomer **26** as an orange solid (52 mg, 0.437 mmol, 62%).

$\boldsymbol{R_f}$ 0.25 (60% ethyl acetate in petroleum ether); **mp** 84-86 °C (CH_2Cl_2; lit.[28] 86-87 °C); **^{1}H NMR (400 MHz, $CDCl_3$)** δ 7.75 (1H, d, *J* 8.6 Hz, H4), 7.52 (1H, d, *J* 8.6 Hz, H5), 2.84 (3H, s, ArCH_3); **^{13}C NMR (100 MHz, $CDCl_3$)** δ 162.4 (Q), 137.7 (Q), 130.1, 126.8, 115.7 (Q), 22.8; **IR ν_{max}/cm^{-1} (neat)** 3060, 2240, 1576, 1546, 1407, 1153; **LRMS (ESI)** found *m/z* 120.0 $[M+H]^+$, $C_6H_6N_3$ requires *m/z* 120.06. The spectroscopic data were found to be in agreement with that reported by Dostal and co-workers.[28]

Methyl 3-cyanopyridazine-4-carboxylate (27)

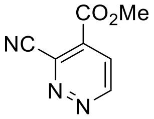
27

According to General Procedure A, triflic anhydride (0.14 mL, 0.84 mmol, 1.2 eq.), methyl pyridazine-4-carboxylate (97 mg, 0.70 mmol, 1.0 eq.), trimethylsilyl cyanide (0.44 mL, 3.50 mmol, 5.0 eq.) and *N*-methylmorpholine (100 μL, 0.91 mmol, 1.3 eq.) gave, following flash column chromatography (40% ethyl acetate in petroleum ether), single isomer **27** as a brown oil (52 mg, 0.319 mmol, 46%).

R R_f 0.35 (80% ethyl acetate in petroleum ether); **^{1}H NMR (500 MHz, $CDCl_3$)** δ 9.58 (1H, d, *J* 5.5 Hz, H6), 8.15 (1H, d, *J* 5.5 Hz, H5), 4.10 (3H, s, CO_2CH_3); **^{13}C NMR (125 MHz, $CDCl_3$)** δ 161.8 (Q), 152.8, 137.4 (Q), 131.1 (Q), 126.3, 114.2 (Q), 54.1; **IR ν_{max}/cm^{-1} (neat)** 3073, 2959, 2245, 1737, 1442, 1336, 1292, 1036; **HRMS (ESI)** found *m/z* 164.04553 $[M+H]^+$, $C_7H_6O_2N_3$ requires *m/z* 164.04545.

4-Phenylpyrimidine-2-carbonitrile (28) and 6-phenylpyrimidine-4-carbonitrile (28’)

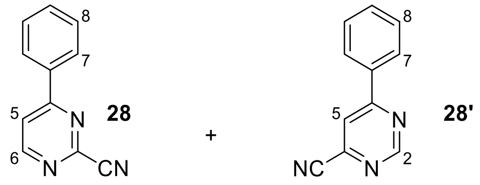

According to General Procedure A, triflic anhydride (0.14 mL, 0.84 mmol, 1.2 eq.), 4-phenylpyrimidine (109 mg, 0.70 mmol, 1.0 eq.), trimethylsilyl cyanide (0.44 mL, 3.50 mmol, 5.0 eq.) and *N*-methylmorpholine (100 μL, 0.91 mmol, 1.3 eq.) gave, following flash column chromatography (5→15% ethyl acetate in petroleum ether), separable isomers **28** as a pale yellow solid (66 mg, 0.364 mmol, 52%) and **28’** as a colorless solid (12 mg, 0.066 mmol, 10%).

28: R_f 0.27 (10% ethyl acetate in petroleum ether); **mp** 156-158 °C (CH_2Cl_2); **^{1}H NMR (400 MHz, $CDCl_3$)** δ 8.83 (1H, d, *J* 5.3 Hz, H6), 8.15-8.11 (2H, m, H7), 7.89 (1H, d, *J* 5.3 Hz, H5), 7.61-7.52 (3H, m, H8 and H9); **^{13}C NMR (100 MHz, $CDCl_3$)** δ 164.4 (Q), 158.4, 145.4 (Q), 134.4 (Q), 132.4, 129.4, 127.4, 118.8, 115.9 (Q); **IR ν_{max}/cm^{-1} (neat)** 3069, 2928, 2245, 1570, 1424, 1366, 1314; **HRMS (ESI)** found *m/z* 182.07153 $[M+H]^+$, $C_{11}H_8N_3$ requires *m/z* 182.07127.

28’: R_f 0.27 (10% ethyl acetate in petroleum ether); **mp** 155-158 °C (CH_2Cl_2; lit.[29] 160-161 °C); **^{1}H NMR (400 MHz, $CDCl_3$)** δ 9.36 (1H, d, *J* 1.4 Hz, H2), 8.14-8.11 (2H, m, H7), 8.04 (1H, d, *J* 1.4 Hz, H5), 7.62-7.54 (3H, m, H8 and H9); **^{13}C NMR (100 MHz, $CDCl_3$)** δ 165.9 (Q), 159.7, 141.9 (Q), 134.6 (Q), 132.5, 129.4, 127.4, 120.1, 115.7 (Q); **IR ν_{max}/cm^{-1} (neat)** 3061, 2918, 1682, 1599, 1547,

1449, 1236; **HRMS (ESI)** found *m/z* 182.07147 $[M+H]^+$, $C_{11}H_8N_3$ requires *m/z* 182.07127. The spectroscopic data were found to be in agreement with that reported by Yamanaka and co-workers.[29]

5-Phenylpyrimidine-4-carbonitrile (29) and 5-phenylpyrimidine-2,4-dicarbonitrile (30)

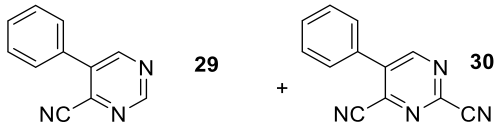

According to General Procedure A, triflic anhydride (0.14 mL, 0.84 mmol, 1.2 eq.), 5-phenylpyrimidine (109 mg, 0.70 mmol, 1.0 eq.), trimethylsilyl cyanide (0.44 mL, 3.50 mmol, 5.0 eq.) and *N*-methylmorpholine (100 µL, 0.91 mmol, 1.3 eq.) gave, following flash column chromatography (0→10% diethyl ether in dichloromethane), separable isomers **29** as an orange, microcrystalline solid (44 mg, 0.24 mmol, 35%) and **30** as a yellow, amorphous solid (15 mg, 0.07 mmol, 10%)

29: $\boldsymbol{R_f}$ 0.42 (5% diethyl ether in dichloromethane **mp** 89-90 °C (CH_2Cl_2); **^{1}H NMR (400 MHz, $CDCl_3$)** δ 9.32 (1H, s, H2), 9.04 (1H, s, H6), 7.64-7.53 (5H, m, Ph*H*); **^{13}C NMR (100 MHz, $CDCl_3$)** δ 158.6, 157.6, 138.7 (Q), 137.7 (Q), 131.5 (Q), 130.3, 129.4, 128.6, 115.0 (Q); **IR ν_{max}/cm^{-1} (neat)** 2239, 1536, 1416, 1397, 758, 718, 698; **HRMS (ESI)** found *m/z* 182.07144 $[M^+H]+$, $C_{11}H_8N_3$ requires *m/z* 182.07127. The spectroscopic data were found to be in agreement with that reported by Yamanaka and co-workers.[30]

30: $\boldsymbol{R_f}$ 0.71 (5% diethyl ether in dichloromethane); **^{1}H NMR (400 MHz, $CDCl_3$)** δ 9.14 (1H, s, H6), 7.65 (5H, s, Ph*H*); **^{13}C NMR (100 MHz, $CDCl_3$)** δ 160.0, 143.4 (Q), 139.7 (Q), 139.6 (Q), 131.5, 130.0 (Q), 129.9, 128.7, 114.4 (Q), 113.7 (Q); **IR ν_{max}/cm^{-1} (neat)** 2246, 1524, 1410, 698. **HRMS (EI)** found *m/z* 206.0583 [M]+, $C_{12}H_6N_4$ requires *m/z* 206.0587.

3-(Methylthio)pyrazine-2-carbonitrile (31)

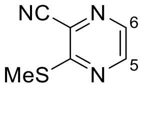

According to General Procedure A, triflic anhydride (0.14 mL, 0.84 mmol, 1.2 eq.), 2-(methylthio)pyrazine (88 mg, 0.70 mmol, 1.0 eq.), trimethylsilyl cyanide (0.44 mL, 3.50 mmol, 5.0 eq.) and *N*-methylmorpholine (100 µL, 0.91 mmol, 1.3 eq.) gave, following flash column chromatography (10% ethyl acetate in petroleum ether), single isomer **31** as a colorless solid (97 mg, 0.642 mmol, 92%).

$\boldsymbol{R_f}$ 0.18 (10% ethyl acetate in petroleum ether); **mp** 80-81 °C (CH_2Cl_2; lit.[31] 83-84 °C); **^{1}H NMR (400 MHz, $CDCl_3$)** δ 7.74 (1H, d, *J* 8.4 Hz, H5), 7.52 (1H, d, *J* 8.4 Hz, H6), 2.83 (3H, s, SC*H*$_3$); **^{13}C**

NMR (100 MHz, $CDCl_3$) δ 162.4 (Q), 137.7 (Q), 130.1, 126.8, 115.7 (Q), 22.8; **IR ν_{max}/cm^{-1} (neat)** 2936, 2231, 1513, 1431, 1355, 1196, 1163, 1087. The spectroscopic data were found to be in agreement with that reported by Metzger and co-workers.[31]

3-Phenylpyrazine-2-carbonitrile (32), 5-phenylpyrazine-2-carbonitrile (32') and 6-phenylpyrazine-2-carbonitrile (32'')

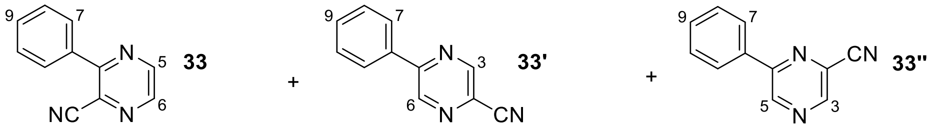

According to General Procedure A, triflic anhydride (74 µL, 0.438 mmol, 1.2 eq.), 2-phenylpyrazine (57 mg, 0.365 mmol, 1.0 eq.), trimethylsilyl cyanide (0.23 mL, 1.83 mmol, 5.0 eq.) and *N*-methylmorpholine (48 µL, 0.474 mmol, 1.3 eq.) gave, following flash column chromatography (5% ethyl acetate in petroleum ether), separable isomer **32** as a colorless solid (42 mg, 0.233 mmol, 66%), **32'** which could not be fully separated from **32** (4.6 mg, 0.025 mmol, 7%) and separable **32''** as a colorless solid (4.3 mg, 0.024 mmol, 7%).

32: $\boldsymbol{R_f}$ 0.09 (20% ethyl acetate in petroleum ether); **mp** 73-75 °C (CH_2Cl_2; lit.[32] 77-78 °C); **^{1}H NMR (400 MHz, $CDCl_3$)** δ 8.83 (1H, d, *J* 2.5 Hz, H5), 8.64 (1H, d, *J* 2.5 Hz, H6), 8.00-7.96 (2H, m, H7), 7.59-7.52 (3H, m, H8 and H9); **^{13}C NMR (100 MHz, $CDCl_3$)** δ 157.1 (Q), 146.6, 143.0, 134.3 (Q), 131.1, 129.0, 129.0, 127.9 (Q), 116.4 (Q); **IR ν_{max}/cm^{-1} (neat)** 3088, 3062, 3038, 2235, 1530, 1432, 1391, 1232, 1168; **HRMS (ESI)** found *m/z* 182.07147 $[M+H]^+$, $C_{11}H_8N_3$ requires *m/z* 182.07127. The spectroscopic data were found to be in agreement with that reported by Petiot and co-workers.[33]

32': $\boldsymbol{R_f}$ 0.16 (20% ethyl acetate in petroleum ether); **^{1}H NMR (400 MHz, $CDCl_3$)** δ 9.22 (1H, s, H3), 8.82 (1H, s, H6), 8.08-8.05 (2H, m, H7), 7.56-7.54 (3H, m, H8 and H9); **^{13}C NMR (100 MHz, $CDCl_3$)** δ 153.5 (Q), 145.8, 144.5, 134.2 (Q), 131.3, 130.1 (Q), 129.3, 127.3, 115.6 (Q); **IR ν_{max}/cm^{-1} (neat)** 3070, 2237, 1599, 1520, 1425, 1312, 1228, 1013 **HRMS (ESI)** found *m/z* 182.07140 $[M+H]^+$, $C_{11}H_8N_3$ requires *m/z* 182.07127. The spectroscopic data were found to be in agreement with that reported by Komeyama and co-workers.[34]

32'': $\boldsymbol{R_f}$ 0.20 (20% ethyl acetate in petroleum ether); **mp** 130-132 °C (CH_2Cl_2); **^{1}H NMR (400 MHz, $CDCl_3$)** δ 9.14 (1H, d, *J* 1.5 Hz, H3), 8.94 (1H, d, *J* 1.5 Hz, H5), 8.11-8.07 (2H, m, H7), 7.58-7.55 (3H, H8 and H9); **^{13}C NMR (100 MHz, $CDCl_3$)** δ 154.8 (Q), 147.6, 142.5, 134.5 (Q), 131.7, 129.5, 128.3 (Q), 127.6, 115.8 (Q); **IR ν_{max}/cm^{-1} (neat)** 3064, 2955, 2930, 2236, 1469, 1446, 1413, 1216, 1150, 1027. The spectroscopic data were found to be in agreement with that reported by Nakamura and co-workers.[35]

5-(Trifluoromethyl)pyrazine-2-carbonitrile (33)

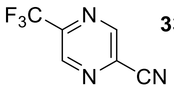

33

According to General Procedure A, triflic anhydride (140 µL, 0.84 mmol, 1.2 eq.), 2-(trifluoromethyl)pyrazine (104 mg, 0.7 mmol, 1.0 eq.), TMSCN (0.44 mL, 3.5 mmol, 5.0 eq.) and *N*-methylmorpholine (100 µL, 0.91 mmol, 1.3 eq.) gave, following flash column chromatography (0→100% ethyl acetate in petroleum ether) single isomer **33** as a colorless oil (63 mg, 0.36 mmol, 52%).

***R*$_f$** 0.55 (20% ethyl acetate in petroleum ether); **^{1}H NMR (400 MHz, $CDCl_3$)** δ 9.10 (1H, *J* 1.2 Hz, H3), 9.05 (1H, *J* 1.2 Hz, H6); **^{13}C NMR (100 MHz, $CDCl_3$)** δ 147.4, 145.7 (Q, q, J_{FC} 36.9 Hz), 142.3, 133.5 (Q), 120.1 (Q, q, J_{FC} 273.7 Hz), 114.3 (Q); **^{19}F NMR (377 MHz, $CDCl_3$)** δ -67.9; **IR ν_{max}/cm^{-1} (neat)** 3095, 2248, 1356, 1312, 1294, 1189, 1147, 1023; **HRMS (EI)** found *m/z* 173.0201 $[M]^+$, $C_6H_2N_3F_3$ requires *m/z* 173.0195.

Phthalazine-1-carbonitrile (34)

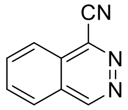

34

According to General Procedure A, triflic anhydride (0.14 mL, 0.84 mmol, 1.2 eq.), phthalazine (91 mg, 0.70 mmol, 1.0 eq.), trimethylsilyl cyanide (0.44 mL, 3.50 mmol, 5.0 eq.) and *N*-methylmorpholine (100 µL, 0.91 mmol, 1.3 eq.) gave, following flash column chromatography (10→100% ethyl acetate in petroleum ether), single isomer **34** as a pale yellow solid (92 mg, 0.591 mmol, 84%).

***R*$_f$** 0.18 (40% ethyl acetate in petroleum ether); **mp** 150-153 °C (CH_2Cl_2; lit.[36] 156-157 °C); **^{1}H NMR (400 MHz, $CDCl_3$)** δ 9.68 (1H, d, *J* 0.7 Hz, H4), 8.32-8.30 (1H, m, H8), 8.17-8.09 (3H, m, H5, H6 and H7); **^{13}C NMR (100 MHz, $CDCl_3$)** δ 152.2, 138.9 (Q), 134.9, 134.6, 127.2, 126.9 (Q), 125.8 (Q), 124.2, 114.1 (Q); **IR ν_{max}/cm^{-1} (neat)** 3063, 2981, 2240, 1613, 1569, 1403, 1371, 1281; **HRMS (ESI)** found *m/z* 156.05582 $[M+H]^+$, $C_9H_6N_3$ requires *m/z* 156.05562. The spectroscopic data were found to be in agreement with that reported by Bhattacharjee and co-workers.[37]

Quinazoline-4-carbonitrile (35)

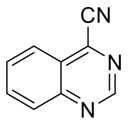

35

According to General Procedure A, triflic anhydride (0.14 mL, 0.84 mmol, 1.2 eq.), quinazoline (91 mg, 0.70 mmol, 1.0 eq.), trimethylsilyl cyanide (0.44 mL, 3.50 mmol, 5.0 eq.) and *N*-methylmorpholine (100 μL, 0.91 mmol, 1.3 eq.) gave, following flash column chromatography (20→30% ethyl acetate in petroleum ether), single isomer **35** as a pink solid (54 mg, 0.348 mmol, 50%).

***R*f** 0.12 (20% ethyl acetate in petroleum ether); **mp** 114-116 °C (CH_2Cl_2; lit.[38] 118-119 °C); **^{1}H NMR (400 MHz, $CDCl_3$)** δ 9.45 (1H, s, H2), 8.28 (1H, d, *J* 8.4 Hz, H8), 8.18 (1H, d, *J* 8.4 Hz, H5), 8.08 (1H, app t, *J* 8.4 Hz, H6), 7.90 (1H, app t, *J* 8.4 Hz, H7); **^{13}C NMR (100 MHz, $CDCl_3$)** δ 154.8, 150.9 (Q), 143.1 (Q), 135.9, 130.4, 129.4, 125.0, 124.6 (Q), 114.2 (Q); **IR ν_{max}/cm^{-1} (neat)** 3070, 3041, 2241, 1612, 1548, 1486, 1340, 1098; **LRMS (ESI)** found *m/z* 177.9 $[M+Na]^+$, $C_9H_5N_3Na$ requires *m/z* 178.0. The spectroscopic data were found to be in agreement with that reported by Wan and co-workers.[39]

[2,2'-Bipyridine]-6-carbonitrile (36) and [2,2'-bipyridine]-4-carbonitrile (36')

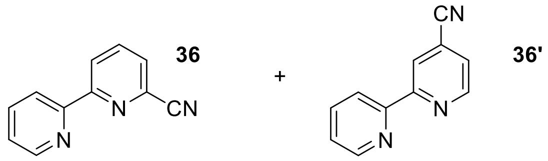

According to General Procedure A, triflic anhydride (140 μL, 0.84 mmol, 1.2 eq.), 2,2'-bipyridine (109 mg, 0.7 mmol, 1.0 eq.), TMSCN (0.44 mL, 3.5 mmol, 5.0 eq.) and *N*-methylmorpholine (100 μL, 0.91 mmol, 1.3 eq.) gave, following flash column chromatography (10→20% ethyl acetate in petroleum ether) separable isomers **36** as a colorless solid (54 mg, 0.30 mmol, 43%) and **36'** as a colorless solid (18 mg, 0.10 mmol, 14%).

36: *R*f 0.09 (20% ethyl acetate in petroleum ether); **mp** 125-128 °C (MeOH; lit.[40] 125 °C); **^{1}H NMR (400 MHz, $CDCl_3$)** δ 8.68 (2H, m, H3 and H6'), 8.47 (1H, d, *J* 8.0 Hz, H3'), 7.96 (1H, app t, *J* 7.6 Hz, H4), 7.87 (1H, m, H4'), 7.71 (1H, d, *J* 7.6 Hz, H5), 7.39 (1H, m, H5'); **^{13}C NMR (100 MHz, $CDCl_3$)** δ 157.7 (Q), 154.0 (Q), 149.3, 137.9, 137.2, 133.2 (Q), 128.1, 124.8, 124.2, 121.6, 117.4 (Q); **IR ν_{max}/cm^{-1} (neat)** 3062, 2924, 2236, 1580, 1557, 1454, 1084, 1041; **HRMS (ESI)** found *m/z*

182.07137 [M+H]$^+$, $C_{11}H_8N_3$ requires *m/z* 182.07127. The spectroscopic data were found to be in agreement with that reported by Liao and co-workers.[40].

36': *R*$_f$ 0.11 (20% ethyl acetate in petroleum ether); **mp** 93-95 °C (MeOH; lit.[41] 86 °C); **^{1}H NMR (400 MHz, CDCl$_3$)** δ 8.83 (1H, dd, *J* 4.9 and 0.7 Hz, H3), 8.72-8.68 (2H, m, H3' and H6), 8.42 (1H, m, H6'), 7.85 (1H, td, *J* 7.7 and 1.9 Hz, H4'), 7.51 (1H, dd, *J* 4.9 and 1.4 Hz, H5), 7.38 (1H, ddd, *J* 7.4, 4.6 and 1.2 Hz, H5'); **^{13}C NMR (100 MHz, CDCl$_3$)** δ 157.4 (Q), 154.0 (Q), 149.9, 149.3, 137.2, 124.78, 124.71, 122.9, 121.3 (Q), 121.2, 116.6 (Q); **IR ν_{max}/cm^{-1} (neat)** 3069, 2240, 1598, 1582, 1547, 1390, 1251, 1212, 1172, 1157, 1094, 1066; **HRMS (ESI)** found *m/z* 182.07138 [M+H]$^+$, $C_{11}H_8N_3$ requires *m/z* 182.07127. The spectroscopic data were found to be in agreement with that reported by Duric and co-workers.[41]

4,4'-Di-*tert*-butyl-[2,2'-bipyridine]-6-carbonitrile (37)

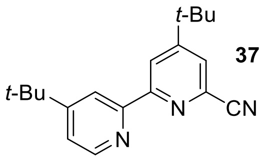

37

According to General Procedure A, triflic anhydride (0.14 mL, 0.84 mmol, 1.2 eq.), 4,4'-di-*tert*-butyl-[2,2'-bipyridine] (188 mg, 0.70 mmol, 1.0 eq.), trimethylsilyl cyanide (0.44 mL, 3.50 mmol, 5.0 eq.) and *N*-methylmorpholine (100 µL, 0.91 mmol, 1.3 eq.) gave, following flash column chromatography (5→40% ethyl acetate in petroleum ether), single isomer **37** as a colorless solid (99 mg, 0.336 mmol, 48%).

***R*$_f$** 0.27 (10% ethyl acetate in petroleum ether); **mp** 175-180 °C (sublimation, CH_2Cl_2); **^{1}H NMR (400 MHz, CDCl$_3$)** δ 8.65 (1H, d, *J* 1.8 Hz, H3), 8.59 (1H, dd, *J* 5.3 and 0.5 Hz, H6'), 8.47 (1H, dd, *J* 1.6 and 0.5 Hz, H3'), 7.69 (1H, d, *J* 1.8 Hz, H5), 7.36 (1H, dd, *J* 5.3 and 1.6 Hz, H5'), 1.40 (9H, s, ArC(C*H*$_3$)$_3$), 1.39 (9H, s, ArC(C*H*$_3$)$_3$); **^{13}C NMR (100 MHz, CDCl$_3$)** δ 162.5 (Q), 161.4 (Q), 156.0 (Q), 154.4 (Q), 149.2, 133.1 (Q), 125.7, 121.7, 121.5, 118.8, 118.0 (Q), 35.4 (Q), 35.1 (Q), 30.6, 30.4; **IR ν_{max}/cm^{-1} (neat)** 2963, 2234, 1592, 1542, 1380; **HRMS (ESI)** found *m/z* 294.19638 [M+H]$^+$, $C_{19}H_{24}N_3$ requires *m/z* 294.19647.

1,10-Phenanthroline-2-carbonitrile (38)

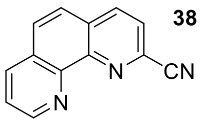

38

According to General Procedure A, triflic anhydride (0.14 mL, 0.84 mmol, 1.2 eq.), 1,10-phenanthroline (126 mg, 0.70 mmol, 1.0 eq.), trimethylsilyl cyanide (0.44 mL, 3.50 mmol, 5.0 eq.) and *N*-methylmorpholine (100 μL, 0.91 mmol, 1.3 eq.) gave, following flash column chromatography (0→2% methanol in CH_2Cl_2), single isomer **38** as a pale brown solid (84 mg, 0.409 mmol, 59%).

***R*$_f$** 0.33 (2% methanol in CH_2Cl_2); **mp** 231-234 °C (CH_2Cl_2; lit.[42] 231-233 °C); **^{1}H NMR (400 MHz, $CDCl_3$)** δ 9.28 (1H, dd, *J* 4.4 and 1.8 Hz, H9), 8.40 (1H, d, *J* 8.4 Hz, H4), 8.31 (1H, dd, *J* 8.2 and 1.8 Hz, H7), 7.97 (1H, d, *J* 8.4 Hz, H3), 7.63 (1H, d, *J* 8.8 Hz, H5), 7.85 (1H, d, *J* 8.8 Hz, H6), 7.73 (1H, dd, *J* 8.2 and 4.4 Hz, H8); **^{13}C NMR (100 MHz, $CDCl_3$)** δ 151.3, 146.8 (Q), 145.4 (Q), 137.3, 136.4, 133.5 (Q), 129.9, 129.3 (Q), 128.9 (Q), 126.3, 125.8, 124.2, 117.5 (Q); **IR ν_{max}/cm^{-1} (neat)** 3059, 2946, 2212, 1469, 1443, 1398, 1356; **LRMS (ESI)** found *m/z* 206.0 $[M+H]^+$, $C_{13}H_8N_3$ requires *m/z* 206.1. The spectroscopic data were found to be in agreement with that reported by Akerboom and co-workers.[43]

Imidazo[1,2-*a*]pyrazine-8-carbonitrile (39)

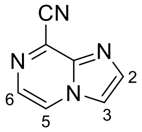

39

According to General Procedure A, triflic anhydride (0.14 mL, 0.84 mmol, 1.2 eq.), imidazo[1,2-*a*]pyrazine (83 mg, 0.70 mmol, 1.0 eq.), trimethylsilyl cyanide (0.44 mL, 3.50 mmol, 5.0 eq.) and *N*-methylmorpholine (100 μL, 0.91 mmol, 1.3 eq.) gave, following flash column chromatography (40→100% ethyl acetate in petroleum ether), single isomer **39** as a colorless solid (70 mg, 0.486 mmol, 69%).

***R*$_f$** 0.21 (100% ethyl acetate in petroleum ether); **mp** 155 °C (decomp. CH_2Cl_2); **^{1}H NMR (400 MHz, DMSO-*d*$_6$)** δ 8.96 (1H, d, *J* 4.3 Hz, H6), 8.38 (1H, d, *J* 1.0 Hz, H3), 8.10 (1H, d, *J* 4.3 Hz, H5), 8.03 (1H, d, *J* 1.0 Hz, H2); **^{13}C NMR (100 MHz, $CDCl_3$)** δ 140.1 (Q), 137.6, 129.8, 125.8 (Q), 124.8, 117.0, 115.1 (Q); **IR ν_{max}/cm^{-1} (neat)** 3154, 2980, 2237, 1599, 1491, 1446, 1370, 1283, 1254, 1142; **HRMS (ESI)** found *m/z* 145.05089 $[M+H]^+$, $C_7H_5N_4$ requires *m/z* 145.05087.

3-Bromoimidazo[1,2-a]pyrazine-8-carbonitrile (40)

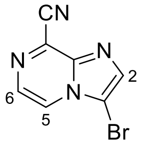

40

According to General Procedure A, triflic anhydride (140 μL, 0.84 mmol, 1.2 eq.), 3-bromoimidazo[1,2-*a*]pyrazine (139 mg, 0.7 mmol, 1.0 eq.), TMSCN (0.44 mL, 3.5 mmol, 5.0 eq.) and *N*-methylmorpholine (100 μL, 0.91 mmol, 1.3 eq.) gave, following flash column chromatography (10→20% ethyl acetate in petroleum ether) single isomer **40** as an off-white amorphous solid (63 mg, 0.36 mmol, 52%).

***R*$_f$** 0.55 (20% ethyl acetate in petroleum ether); **mp** 175-177 °C ($CHCl_3$); **^{1}H NMR (400 MHz, $CDCl_3$)** δ 8.29 (1H, d *J* 4.6 Hz, H5), 8.17 (1H, d *J* 4.6 Hz, H6), 7.97 (1H, s, H2); **^{13}C NMR (100 MHz, $CDCl_3$)** δ 140.8 (Q), 137.9, 130.7, 127.6 (Q), 119.6, 113.2 (Q), 99.4 (Q); **IR ν_{max}/cm^{-1} (neat)** 3116, 3085, 3013, 2929, 2245, 1880, 1760, 1711, 1643, 1593, 1488, 1472, 1429, 1351, 1329, 1283, 1238, 1224, 1337, 1128, 1087; **HRMS (ESI)** found *m/z* 222.96159 $[M+H]^+$, $C_7H_4N_4{}^{79}Br$ requires *m/z* 222.96139.

2-(Trifluoromethyl)imidazo[1,2-a]pyrazine-3-carbonitrile (41)

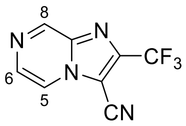

41

According to General Procedure A, triflic anhydride (0.14 mL, 0.84 mmol, 1.2 eq.), 2-(trifluoromethyl)imidazo[1,2-*a*]pyrazine (131 mg, 0.70 mmol, 1.0 eq.), trimethylsilyl cyanide (0.44 mL, 3.50 mmol, 5.0 eq.) and *N*-methylmorpholine (100 μL, 0.91 mmol, 1.3 eq.) gave, following flash column chromatography (20→40% ethyl acetate in petroleum ether), single isomer **42** as a pale yellow solid (135 mg, 0.636 mmol, 91%).

***R*$_f$** 0.19 (40% ethyl acetate in petroleum ether); **mp** 119-122 °C (CH_2Cl_2); **^{1}H NMR (500 MHz, $CDCl_3$)** δ 8.45 (1H, d, *J* 4.6 Hz, H6), 8.23 (1H, s, H8), 8.19 (1H, d, *J* 4.6 Hz, H5); **^{13}C NMR (125 MHz, $CDCl_3$)** δ 139.7 (Q), 139.3 (Q, q, J_{FC} 40.2 Hz), 131.0, 128.9 (Q), 122.5, 120.5 (Q, q, J_{FC} 266.5 Hz), 144.1, 113.1 (Q); **^{19}F NMR (377 MHz, $CDCl_3$)** δ -63.0; **IR ν_{max}/cm^{-1} (neat)** 3119, 2235, 1495, 1355, 1229, 1139, 1114; **HRMS (ESI)** found *m/z* 211.02328 $[M-H]^-$, $C_8H_2N_4F_3$ requires *m/z* 211.02370.

3-Bromopyrazolo[1,5-*a*]pyrimidine-7-carbonitrile (42) and 3-bromopyrazolo[1,5-*a*]pyrimidine-5-carbonitrile (42')

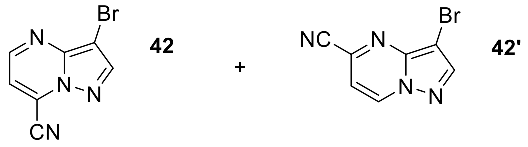

According to General Procedure A, triflic anhydride (140 μL, 0.84 mmol, 1.2 eq.), 3-bromopyrazolo[1,5-*a*]pyrimidine (139 mg, 0.7 mmol, 1.0 eq.), TMSCN (0.44 mL, 3.5 mmol, 5.0 eq.) and *N*-methylmorpholine (100 μL, 0.91 mmol, 1.3 eq.) gave, following flash column chromatography (10→20% ethyl acetate in petroleum ether) separable isomers **42** as yellow crystals (92 mg, 0.41 mmol, 59%) and **42'** as yellow crystals (48 mg, 0.22 mmol, 31%).

42: $\boldsymbol{R_f}$ 0.18 (20% ethyl acetate in petroleum ether); **mp** 186-188 °C (MeOH); **^{1}H NMR (400 MHz, $CDCl_3$)** δ 8.65 (1H, d, *J* 4.0 Hz, H5), 8.30 (1H, s, H2), 7.32 (1H, d, *J* 4.0 Hz, H6); **^{13}C NMR (100 MHz, $CDCl_3$)** δ 148.4, 146.3, 145.4 (Q), 114.5, 114.5 (Q), 109.9 (Q), 88.1 (Q); **IR ν_{max}/cm^{-1} (neat)** 3140, 3077, 3034, 2243, 1607, 1528, 1451, 1362, 1295, 1280, 1237, 1216, 1147, 1078, 1049; **HRMS (ESI)** found *m/z* 220.94657 [M-H$^+$]$^-$, $C_7H_2N_4{}^{79}Br$ requires *m/z* 220.94683.

42': $\boldsymbol{R_f}$ 0.10 (20% ethyl acetate in petroleum ether); **mp** 156-158 °C (MeOH);**^{1}H NMR (400 MHz, $CDCl_3$)** δ 8.80 (1H, d, *J* 4.0 Hz, H6), 8.31 (1H, s, H2),7.13 (1H, d, *J* 4.0 Hz, H7); **^{13}C NMR (125 MHz, $CDCl_3$)** δ 147.2, 144.6 (Q), 136.7, 132.3 (Q), 115.5 (Q), 110.2, 88.4 (Q); **IR ν_{max}/cm^{-1} (neat)** 3093, 2923, 2852, 2236, 1900, 1797, 1600, 1540, 1522, 1505, 1443, 1400, 1316, 1302, 1267, 1234, 1187, 1234, 1187, 1156, 1108. 1048, 1009; **HRMS (ESI)** found *m/z* 220.94698 [M-H]$^-$, $C_7H_2N_4{}^{79}Br$ requires *m/z* 220.94683.

1-Methyl-1*H*-pyrazolo[3,4-*b*]pyridine-4-carbonitrile (43) and 1-methyl-1*H*-pyrazolo[3,4-*b*]pyridine-3-carbonitrile (43')

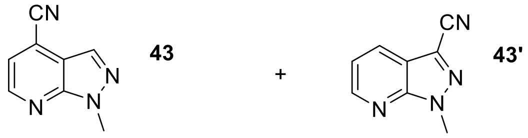

According to General Procedure A, triflic anhydride (0.14 mL, 0.84 mmol, 1.2 eq.), 1-methyl-1*H*-pyrazolo[3,4-*b*]pyridine (93 mg, 0.70 mmol, 1.0 eq.), trimethylsilyl cyanide (0.44 mL, 3.50 mmol, 5.0 eq.) and *N*-methylmorpholine (100 μL, 0.91 mmol, 1.3 eq.) gave, following flash column chromatography (30% ethyl acetate in petroleum ether), separable isomer **43** as a colorless solid (37 mg, 0.231 mmol, 33%) and **43'** which could not be fully separated from **43** (11 mg, 0.070 mmol, 10%).

43: $\boldsymbol{R}_{\mathbf{f}}$ 0.26 (40% ethyl acetate in petroleum ether); **mp** 118-120 °C (sublimation, CH_2Cl_2); **^{1}H NMR (400 MHz, $CDCl_3$)** δ 8.68 (1H, d, *J* 4.4 Hz, H6), 8.20 (1H, s, H3), 7.41 (1H, d, *J* 4.4 Hz, H5), 4.21 (3H, s, NC*H*$_3$); **^{13}C NMR (100 MHz, $CDCl_3$)** δ 150.2 (Q), 148.5, 130.6, 119.9, 115.3 (Q), 114.2 (Q), 112.8 (Q), 34.4; **IR ν_{max}/cm^{-1} (neat)** 3110, 3086, 2243, 1586, 1572, 1334; **HRMS (ESI)** found *m/z* 159.06662 $[M+H]^+$, $C_8H_7N_4$ requires *m/z* 159.06652.

43': $\boldsymbol{R}_{\mathbf{f}}$ 0.19 (40% ethyl acetate in petroleum ether); **^{1}H NMR (400 MHz, $CDCl_3$)** δ 8.69-8.67 (1H, m, H6), 8.21-8.19 (1H, m, H4), 7.35 (1H, dd, *J* 8.2 and 4.5 Hz, H5), 4.25 (3H, s, NC*H*$_3$); **^{13}C NMR (100 MHz, $CDCl_3$)** δ 161.5 (Q), 161.4 (Q), 150.4, 128.8, 119.4, 117.1 (Q), 113.0 (Q), 35.1; **IR ν_{max}/cm^{-1} (neat)** 3109, 3086, 2237, 1586, 1572, 1425, 1360, 1266; **HRMS (ESI)** found *m/z* 159.06665 $[M+H]^+$, $C_8H_7N_4$ requires *m/z* 159.06652.

3-Bromo-1-methyl-1*H*-pyrrolo[2,3-*b*]pyridine-4-carbonitrile (44)

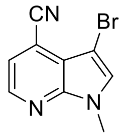

44

According to General Procedure A, triflic anhydride (0.14 mL, 0.84 mmol, 1.2 eq.), 3-bromo-1-methyl-1*H*-pyrrolo[2,3-*b*]pyridine (148 mg, 0.70 mmol, 1.0 eq.), trimethylsilyl cyanide (0.44 mL, 3.50 mmol, 5.0 eq.) and *N*-methylmorpholine (100 μL, 0.91 mmol, 1.3 eq.) gave, following flash column chromatography (20→80% ethyl acetate in petroleum ether), single isomer **44** as an orange solid (30 mg, 0.127 mmol, 18%).

$\boldsymbol{R}_{\mathbf{f}}$ 0.11 (20% ethyl acetate in petroleum ether); **mp** 180 °C (decomp.CH_2Cl_2); **^{1}H NMR (400 MHz, $CDCl_3$)** δ 8.41 (1H, d, *J* 5.0 Hz, H6), 7.42 (1H, s, H2), 7.38 (1H, d, *J* 5.0 Hz, H5), 3.91 (3H, s, NC*H*$_3$); **^{13}C NMR (100 MHz, $CDCl_3$)** δ 147.0 (Q), 143.2, 131.7, 120.2, 117.5 (Q), 115.4 (Q), 110.7 (Q), 86.8 (Q), 31.7; **IR ν_{max}/cm^{-1} (neat)** 3097, 3065, 2232, 1716, 1576, 1563, 1509, 1374, 1275; **HRMS (ESI)** found *m/z* 235.98216 $[M+H]^+$, $C_9H_7N_3{}^{79}Br$ requires *m/z* 235.98179.

7-Methyl-7*H*-pyrrolo[2,3-*d*]pyrimidine-4-carbonitrile (45)

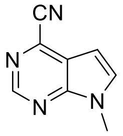

45

According to General Procedure A, triflic anhydride (0.14 mL, 0.84 mmol, 1.2 eq.), 7-methyl-7*H*-pyrrolo[2,3-*d*]pyrimidine (93 mg, 0.70 mmol, 1.0 eq.), trimethylsilyl cyanide (0.44 mL, 3.50 mmol, 5.0 eq.) and *N*-methylmorpholine (100 μL, 0.91 mmol, 1.3 eq.) gave, following flash column

chromatography (40→100% ethyl acetate in petroleum ether), single isomer **45** as a brown solid (36 mg, 0.224 mmol, 32%).

***R*f** 0.58 (2% methanol in CH_2Cl_2); **mp** 124-126 °C (CH_2Cl_2); **^{1}H NMR (400 MHz, $CDCl_3$)** δ 8.93 (1H, s, H6), 7.43 (1H, d, *J* 3.6 Hz, H2), 6.72 (1H, d, *J* 3.6 Hz, H3), 3.93 (3H, s, NC*H*$_3$); **^{13}C NMR (100 MHz, $CDCl_3$)** δ 151.6 (Q), 151.3, 133.7, 131.2 (Q), 120.7 (Q), 115.4 (Q), 98.6, 31.4; **IR ν_{max}/cm^{-1} (neat)** 3113, 3087, 2241, 1585, 1562, 1520, 1370, 1343, 1233; **HRMS (ESI)** found *m/z* 159.06657 $[M+H]^+$, $C_8H_7N_4$ requires *m/z* 159.06652.

7-Bromo-1-methyl-2-phenyl-1*H*-imidazo[4,5-*c*]pyridine-4-carbonitrile (46)

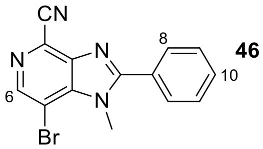

According to General Procedure A, triflic anhydride (71 µL, 0.42 mmol, 1.2 eq.), 7-bromo-1-methyl-2-phenyl-1*H*-imidazo[4,5-*c*]pyridine (101 mg, 0.35 mmol, 1.0 eq.), trimethylsilyl cyanide (0.22 mL, 1.75 mmol, 5.0 eq.) and *N*-methylmorpholine (50 µL, 0.455 mmol, 1.3 eq.) gave, following flash column chromatography (20→40% ethyl acetate in petroleum ether), single isomer **46** as a pale yellow solid (95 mg, 0.302 mmol, 86%).

***R*f** 0.38 (40% ethyl acetate in petroleum ether); **mp** 210-213 °C (CH_2Cl_2); **^{1}H NMR (400 MHz, $CDCl_3$)** δ 8.52 (1H, s, H6), 7.77-7.74 (2H, m, H8), 7.61-7.54 (3H, m, H9 and H10); **^{13}C NMR (100 MHz, $CDCl_3$)** δ 159.5 (Q), 145.1, 143.4 (Q), 139.4 (Q), 131.4, 130.0, 129.1, 127.8 (Q), 123.9 (Q), 115.1 (Q), 105.8 (Q), 34.7; **IR ν_{max}/cm^{-1} (neat)** 3063, 2960, 2231, 1592, 1471, 1318, 1287, 1162; **HRMS (ESI)** found *m/z* 313.00845 $[M+H]^+$, $C_{14}H_{10}N_4{}^{79}Br$ requires *m/z* 313.00834.

5-Bromo-2-cyclobutylpyrimidine-4-carbonitrile (47)

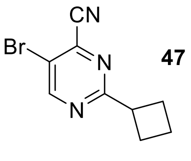

According to General Procedure A, triflic anhydride (40 µL, 0.24 mmol, 1.2 eq.), 5-bromo-2-cyclobutylpyrimidine (43 mg, 0.20 mmol, 1.0 eq.), trimethylsilyl cyanide (0.13 mL, 1.00 mmol, 5.0 eq.) and *N*-methylmorpholine (29 µL, 0.26 mmol, 1.3 eq.) gave, following flash column chromatography (10% ethyl acetate in petroleum ether), single isomer **47** as a colorless oil (36 mg, 0.151 mmol, 75%).

***R*f** 0.24 (10% ethyl acetate in petroleum ether); **^{1}H NMR (400 MHz, $CDCl_3$)** 8.92 (1H, s, H6), 3.85-3.76 (1H, m, ArC*H*(CH_2)$_2$), 2.45-2.35 (4H, m, C*H*$_2$), 2.15-2.03 (1H, m, ArCHC*H*$_2$), 2.00-1.90 (1H, m,

ArCHCH_2); **^{13}C NMR (100 MHz, CDCl$_3$)** δ 172.9 (Q), 160.1, 147.6 (Q), 119.5 (Q), 114.3 (Q), 42.2, 27.8, 18.2; **IR ν_{max}/cm^{-1} (neat)** 2988, 2944, 2243, 1541, 1524, 1416, 1219, 1148, 1048; **HRMS (ESI)** found *m/z* 235.98271 [M-H]$^-$, $C_9H_7N_3{}^{79}Br$ requires *m/z* 235.98288.

4-(1-Methyl-1*H*-pyrazol-4-yl)pyrimidine-2-carbonitrile (48) and 6-(1-methyl-1*H*-pyrazol-4-yl)pyrimidine-2,4-dicarbonitrile (49)

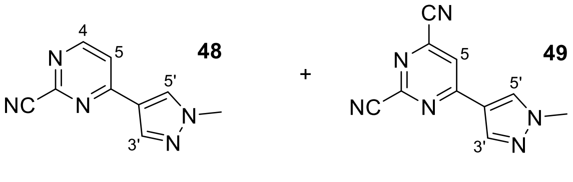

According to General Procedure A, triflic anhydride (0.11 mL, 0.667 mmol, 1.2 eq.), 6-(1-methyl-1*H*-pyrazol-4-yl)pyrimidine-2,4-dicarbonitrile (89 mg, 0.556 mmol, 1.0 eq.), trimethylsilyl cyanide (0.348 mL, 2.78 mmol, 5.0 eq.) and *N*-methylmorpholine (79 μL, 0.722 mmol, 1.3 eq.) gave, following flash column chromatography (60→100% ethyl acetate in petroleum ether), separable **48** as a brown solid (29 mg, 0.157 mmol, 28%) and **49** as a pale yellow solid (22 mg, 0.105 mmol, 19%).

48: ***R*$_f$** 0.05 (60% ethyl acetate in petroleum ether); **mp** 157-159 °C (CH_2Cl_2); **^{1}H NMR (400 MHz, CDCl$_3$)** δ 8.66 (1H, d, *J* 5.6 Hz, H4), 8.12 (1H, s, H3'), 8.03 (1H, s, H5'), 7.51 (1H, d, *J* 5.6 Hz, H5), 3.98 (3H, s, NCH_3); **^{13}C NMR (100 MHz, CDCl$_3$)** δ 160.2 (Q), 157.7, 145.4 (Q), 138.7, 131.3, 119.9 (Q), 117.8, 115.9 (Q), 39.6; **IR ν_{max}/cm^{-1} (neat)** 3118, 2231, 1590, 1516, 1381, 1229; **HRMS (ESI)** found *m/z* 186.07762 [M+H]$^+$, $C_9H_8N_5$ requires *m/z* 186.07742.

49: ***R*$_f$** 0.13 (60% ethyl acetate in petroleum ether); **mp** 164-166 °C (CH_2Cl_2); **^{1}H NMR (500 MHz, CDCl$_3$)** δ 8.19 (1H, s, H3'), 8.09 (1H, s, H5'), 7.77 (1H, s, H5), 4.02 (3H, s, NCH_3); **^{13}C NMR (125 MHz, CDCl$_3$)** δ 162.3 (Q), 145.9 (Q), 142.0 (Q), 139.3, 132.1, 120.7, 117.8 (Q), 114.7 (Q), 114.3 (Q), 39.8; **IR ν_{max}/cm^{-1} (neat)** 3117, 2245, 1560, 1507, 1326, 1300, 1209, 1004; **HRMS (ESI)** found *m/z* 211.07278 [M+H]$^+$, $C_{10}H_7N_6$ requires *m/z* 211.07267.

2-((2-Methoxyphenyl)(methyl)amino)pyrimidine-4-carbonitrile (50)

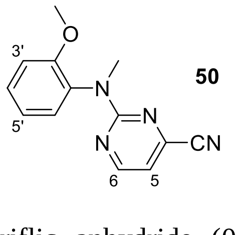

According to General Procedure A, triflic anhydride (0.14 mL, 0.84 mmol, 1.2 eq.), 2-((2-methoxyphenyl)(methyl)amino)pyrimidine (151 mg, 0.70 mmol, 1.0 eq.), trimethylsilyl cyanide (0.44 mL, 3.50 mmol, 5.0 eq.) and *N*-methylmorpholine (100 μL, 0.91 mmol, 1.3 eq.) gave, following flash

column chromatography (40% ethyl acetate in petroleum ether), single isomer **50** as a yellow solid (60 mg, 0.25 mmol, 36%).

***R*f** 0.31 (40% ethyl acetate in petroleum ether); **mp** 146-148 °C (CH_2Cl_2); **^{1}H NMR (400 MHz, $CDCl_3$)** δ 8.37 (1H, app br s, H6), 7.32 (1H, app td, *J* 8.0 and 1.6 Hz, H4'), 7.21 (1H, dd, *J* 8.0 and 1.6 Hz, H6'), 7.05-6.99 (2H, m, H3' and H5'), 6.81 (1H, d, *J* 4.6 Hz, H5), 3.77 (3H, s, OC*H*$_3$), 3.42 (3H, s, NC*H*$_3$); **^{13}C NMR (100 MHz, $CDCl_3$)** δ 162.1 (Q), 159.5, 155.1 (Q), 141.5 (Q), 132.8, 128.8, 121.2, 120.9 (Q), 116.3 (Q), 112.8, 112.3, 55.6, 38.1; **IR ν_{max}/cm^{-1} (neat)** 2942, 2234, 1571, 1542, 1499, 1345, 1159; **HRMS (ESI)** found *m/z* 241.10846 $[M+H]^+$, $C_{13}H_{13}N_4O$ requires *m/z* 241.10839.

2-(Methylthio)-6-(thiophen-2-yl)pyrimidine-4-carbonitrile (51)

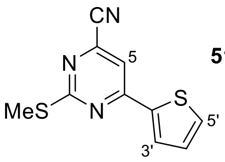

51

According to General Procedure A, triflic anhydride (0.14 mL, 0.84 mmol, 1.2 eq.), 2-(methylthio)-6-(thiophen-2-yl)pyrimidine (146 mg, 0.70 mmol, 1.0 eq.), trimethylsilyl cyanide (0.44 mL, 3.50 mmol, 5.0 eq.) and *N*-methylmorpholine (100 µL, 0.91 mmol, 1.3 eq.) gave, following flash column chromatography (5% ethyl acetate in petroleum ether), single isomer **51** as a yellow solid (131 mg, 0.561 mmol, 80%).

***R*f** 0.28 (10% ethyl acetate in petroleum ether); **mp** 165-167 °C (CH_2Cl_2); **^{1}H NMR (500 MHz, $CDCl_3$)** δ 7.81 (1H, dd, *J* 3.9 and 1.2 Hz, H3'), 7.63 (1H, dd, *J* 4.9 and 1.2 Hz, H5'), 7.47 (1H, s, H5), 7.19 (1H, dd, *J* 4.9 and 3.9 Hz, H4'), 2.61 (3H, s, SC*H*$_3$); **^{13}C NMR (125 MHz, $CDCl_3$)** δ 174.9 (Q), 160.3 (Q), 141.3 (Q), 140.3 (Q), 132.5, 129.6, 128.9, 115.6 (Q), 113.4, 14.3; **IR ν_{max}/cm^{-1} (neat)** 3085, 3011, 2245, 1569, 1511, 1428, 1342, 1270, 1226; **HRMS (ESI)** found *m/z* 234.01559 $[M+H]^+$, $C_{10}H_8N_3S$ requires *m/z* 234.01542.

(3*S*,8*R*,9*S*,10*R*,13*S*,14*S*)-17-(4-Cyanopyridin-3-yl)-10,13-dimethyl-2,3,4,7,8,9,10,11,12,13,14,15-dodecahydro-1*H*-cyclopenta[*a*]phenanthren-3-yl acetate (52), (3*S*,8*R*,9*S*,10*R*,13*S*,14*S*)-17-(6-cyanopyridin-3-yl)-10,13-dimethyl-2,3,4,7,8,9,10,11,12,13,14,15-dodecahydro-1*H*-cyclopenta[*a*]phenanthren-3-yl acetate (52') and (3*S*,8*R*,9*S*,10*R*,13*S*,14*S*)-17-(2-cyanopyridin-3-yl)-10,13-dimethyl-2,3,4,7,8,9,10,11,12,13,14,15-dodecahydro-1*H*-cyclopenta[*a*]phenanthren-3-yl acetate (52'')

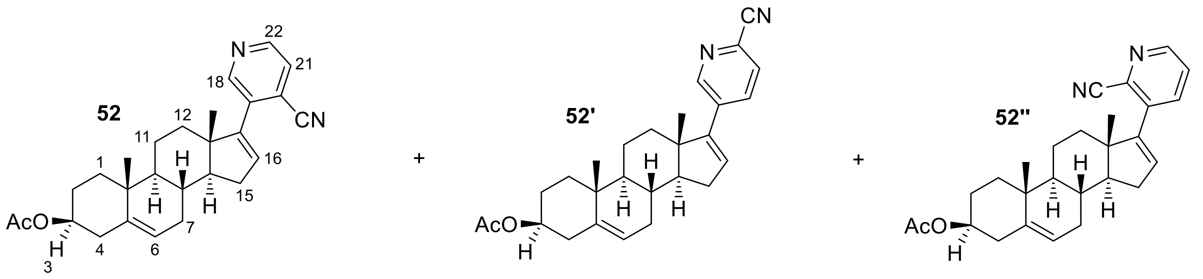

According to General Procedure A, triflic anhydride (71 µL, 0.42 mmol, 1.2 eq.), abiraterone acetate (137 mg, 0.35 mmol, 1.0 eq.), trimethylsilyl cyanide (0.22 mL, 1.75 mmol, 5.0 eq.) and *N*-methylmorpholine (50 µL, 0.455 mmol, 1.3 eq.) gave, following flash column chromatography (20% ethyl acetate in petroleum ether), separable isomers **52** as a pale yellow solid (97 mg, 0.233 mmol, 67%), **52'** as a colorless oil (3 mg, 0.007 mmol, 2%) and **52''** which could not be fully separated from **52** (6 mg, 0.014 mmol, 4%).

52: R_f 0.11 (20% ethyl acetate in petroleum ether); **mp** 224-226 °C (CH_2Cl_2); **^{1}H NMR (400 MHz, $CDCl_3$)** δ 8.67 (1H, s, H18), 8.61 (1H, d, *J* 5.1 Hz, H22), 7.51 (1H, d, *J* 5.1 Hz, H21), 6.17-6.15 (1H, m, H6), 5.42-5.39 (1H, m, H16), 4.64-4.55 (1H, m, H3), 2.42-2.28 (3H, m, H4, H7 and H7'), 2.20-2.12 (1H, m, H4'), 2.10-2.03 (1H, m, H15), 2.01 (3H, s, COC*H*$_3$), 1.89-1.81 (2H, m, H1 and H2), 1.78-1.50 (8H, m, H2', H8, H11, H11', H12, H12', H14, and H15'), 1.18-1.07 (2H, m, H1' and H9), 1.05 (3H, s, CC*H*$_3$), 1.01 (3H, s, CC*H*$_3$); **^{13}C NMR (100 MHz, $CDCl_3$)** δ 170.5 (Q), 149.6, 148.2 (Q), 148.2, 140.0 (Q), 135.2 (Q), 135.1, 126.0, 122.2, 119.8 (Q), 116.7 (Q), 73.7, 57.1, 50.2, 49.4 (Q), 38.1, 36.9, 36.8 (Q), 34.9, 32.5, 31.4, 30.6, 27.7, 21.4, 20.7, 19.2, 16.6; **IR ν_{max}/cm^{-1} (neat)** 3046, 2943, 2900, 2827, 2234, 1731, 1367, 1247, 1035; **HRMS (ESI)** found *m/z* 417.25357 $[M+H]^+$, $C_{27}H_{33}N_2O_2$ requires *m/z* 417.25365.

52': R_f 0.26 (20% ethyl acetate in petroleum ether); **^{1}H NMR (500 MHz, $CDCl_3$)** δ 8.72 (1H, d, *J* 1.9 Hz, H18), 7.76 (1H, dd, *J* 8.1 and 1.9 Hz, H20), 7.62 (1H, d, *J* 8.1 Hz, H21), 6.21-6.20 (1H, m, H6), 5.43-5.41 (1H, m, H16), 4.65-4.58 (1H, m, H3), 2.39-2.30 (3H, m, H4, H7 and H7'), 2.14-2.03 (3H, m, H1, H4', H15), 2.05 (3H, s, COC*H*$_3$), 1.91-1.84 (2H, m, H2 and H8), 1.81-1.58 (7H, m, H2', H11, H11', H12, H12', H14, and H15'), 1.52-1.45 (1H, m, H1'), 1.20-1.12 (1H, m, H9), 1.08 (3H, s, CC*H*$_3$), 1.07 (3H, s, CC*H*$_3$); **^{13}C NMR (125 MHz, $CDCl_3$)** δ 170.6 (Q), 150.4 (Q), 149.1, 140.1 (Q), 136.3 (Q), 133.7, 133.1, 131.4 (Q), 128.0, 122.1, 117.5 (Q), 73.8, 57.4, 50.1, 47.4 (Q), 38.1, 36.9, 36.8 (Q), 35.1, 32.1, 31.4, 30.3, 27.7, 21.4, 20.8, 19.3, 16.7; **IR ν_{max}/cm^{-1} (neat)** 2937, 2905, 2853,

2234, 1730, 1375, 1245, 1033; **HRMS (ESI)** found *m/z* 417.25365 $[M+H]^+$, $C_{27}H_{33}N_2O_2$ requires *m/z* 417.25365.

52'': *R*f 0.23 (20% ethyl acetate in petroleum ether); **^{1}H NMR (500 MHz, $CDCl_3$)** δ 8.58 (1H, dd, *J* 4.7 and 1.4 Hz, H22), 7.66 (1H, dd, *J* 8.1 and 1.4 Hz, H20), 7.43 (1H, dd, *J* 8.1 and 4.7 Hz, H21), 6.17-6.15 (1H, m, H6), 5.42-5.39 (1H, m, H16), 4.64-4.55 (1H, m, H3), 2.42-2.28 (3H, m, H4, H7 and H7'), 2.20-2.12 (1H, m, H4'), 2.10-2.03 (1H, m, H15), 2.01 (3H, s, $COCH_3$), 1.89-1.81 (2H, m, H1 and H2), 1.78-1.50 (8H, m, H2', H8, H11, H11', H12, H12', H14, and H15'), 1.18-1.07 (2H, m, H1' and H9), 1.05 (3H, s, CCH_3), 1.01 (3H, s, CCH_3); **^{13}C NMR (125 MHz, $CDCl_3$)** δ 170.5 (Q), 149.1, 148.2 (Q), 139.9, 138.2 (Q), 136.0, 134.3, 133.0 (Q), 125.6, 122.3 (Q), 117.5 (Q), 73.8, 57.1, 50.2, 49.4 (Q), 38.1, 36.9, 36.8 (Q), 35.0, 32.5, 31.5, 30.6, 27.7, 21.4, 20.7, 19.3, 16.6; **HRMS (ESI)** found *m/z* 417.25361 $[M+H]^+$, $C_{27}H_{33}N_2O_2$ requires *m/z* 417.25365.

N-(2-Cyano-6,7-bis(2-methoxyethoxy)quinazolin-4-yl)-N-(3-ethynylphenyl)acetamide (53)

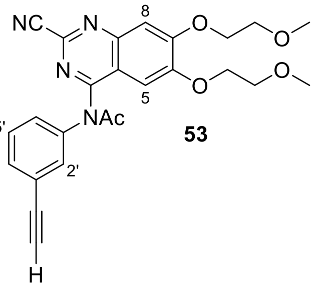

53

According to General Procedure A, triflic anhydride (67 µL, 0.398 mmol, 1.2 eq.), erlotinib acetate (170 mg, 0.332 mmol, 1.0 eq.), trimethylsilyl cyanide (0.21 mL, 1.66 mmol, 5.0 eq.) and *N*-methylmorpholine (47 µL, 0.431 mmol, 1.3 eq.) gave, following flash column chromatography (60→100% ethyl acetate in petroleum ether), single isomer **53** as a brown solid (75 mg, 0.163 mmol, 49%).

***R*f** 0.33 (100% ethyl acetate); **mp** 161-164 °C (CH_2Cl_2); **^{1}H NMR (400 MHz, $CDCl_3$)** δ 7.45-7.42 (2H, m, H2' and H6'), 7.41-7.35 (3H, m, H4', H5' and H8), 7.19 (1H, s, H5), 4.34-4.31 (2H, m, $ArOCH_2CH_2OCH_3$), 4.22-4.20 (2H, m, $ArOCH_2CH_2OCH_3$), 3.87-3.85 (2H, m, $ArOCH_2CH_2OCH_3$), 3.83-3.80 (2H, m, $ArOCH_2CH_2OCH_3$), 3.46 (3H, s, $ArOCH_2CH_2OCH_3$), 3.45 (3H, s, $ArOCH_2CH_2OCH_3$), 3.10 (1H, s, CC*H*), 2.19 (3H, s, $NCOCH_3$); **^{13}C NMR (100 MHz, $CDCl_3$)** δ 170.6 (Q), 159.2 (Q), 157.1 (Q), 152.8 (Q), 150.9 (Q), 140.6 (Q), 138.5 (Q), 131.8, 131.1, 129.7, 128.4, 123.8 (Q), 118.1 (Q), 116.2 (Q), 108.0, 103.4, 82.1 (Q), 78.9, 70.6, 70.2, 69.2, 69.1, 59.4, 59.4, 23.7; **IR ν_{max}/cm^{-1} (neat)** 3259, 2930, 2886, 2816, 2237, 2105, 1676, 1498, 1370, 1217; **HRMS (ESI)** found *m/z* 461.18173 $[M+H]^+$, $C_{25}H_{25}N_4O_5$ requires *m/z* 461.18195.

2-Methyl-1,2,3,4,10,14*b*-hexahydrobenzo[*c*]pyrazino[1,2-*a*]pyrido[3,2-*f*]azepine-9-carbonitrile (54)

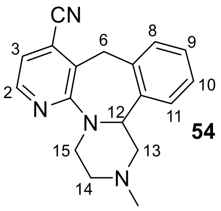

54

According to General Procedure A, triflic anhydride (71 µL, 0.42 mmol, 1.2 eq.), mirtazapine (93 mg, 0.35 mmol, 1.0 eq.), trimethylsilyl cyanide (0.22 mL, 1.75 mmol, 5.0 eq.) and *N*-methylmorpholine (50 µL, 0.455 mmol, 1.3 eq.) gave, following flash column chromatography (50→100% ethyl acetate in petroleum ether, then 0→10% methanol in CH_2Cl_2), single isomer **54** as a yellow oil (33 mg, 0.114 mmol, 33%) and recovered mirtazapine (59 mg, 63%).

***R*$_f$** 0.29 (60% ethyl acetate in petroleum ether); **^{1}H NMR (500 MHz, $CDCl_3$)** δ 8.08-8.06 (1H, m, Ar*H*), 7.37-7.31 (2H, m, Ar*H*), 7.30-7.26 (1H, m, Ar*H*), 7.24-7.22 (1H, m, Ar*H*), 6.70-6.68 (1H, m, Ar*H*), 5.55 (1H, app s, H12), 4.28 (1H, d, *J* 16.0 Hz, H6), 4.03 (1H, dt, *J* 14.4 and 5.6 Hz, H14 or H15) 3.82 (1H, dt, *J* 14.4 and 6.2 Hz, H14 or H15), 3.70 (1H, d, *J* 16.0 Hz, H6'), 3.63 (1H, d, *J* 17.4 Hz, H13), 3.60 (1H, d, *J* 17.4 Hz, H13'), 2.90-2.80 (2H, m, H14 or H15), 2.48 (3H, s, NC*H*$_3$); **^{13}C NMR (125 MHz, $CDCl_3$)** δ 154.8 (Q), 146.4, 139.0 (Q), 138.1, 130.7 (Q), 130.4, 128.9, 127.7, 120.9 (Q), 118.7 (Q), 115.9, 114.9 (Q), 56.5, 55.0, 49.3, 45.9, 42.3, 38.6; **IR ν_{max}/cm^{-1} (neat)** 2956, 2858, 2806, 2237, 1589, 1568, 1468, 1457, 1422, 1252, 1115; **HRMS (ESI)** found *m/z* 291.16055 $[M+H]^+$, $C_{18}H_{19}N_4$ requires *m/z* 291.16042.

6-Methoxy-4-((*R*)-methoxy((1*S*,2*S*,4*S*,5*R*)-5-vinylquinuclidin-2-yl)methyl)quinoline-2-carbonitrile (55)

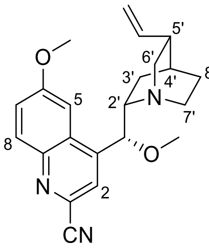

55

According to General Procedure A, triflic anhydride (0.14 mL, 0.84 mmol, 1.2 eq.), *O*-methylquinine (237 mg, 0.70 mmol, 1.0 eq.), trimethylsilyl cyanide (0.44 mL, 3.50 mmol, 5.0 eq.) and *N*-methylmorpholine (100 µL, 0.91 mmol, 1.3 eq.) gave, following flash column chromatography (0→10% methanol in CH_2Cl_2), single isomer **55** as an orange oil (135 mg, 0.371 mmol, 53%).

***R*$_f$** 0.26 (10% methanol in CH_2Cl_2); **^{1}H NMR (400 MHz, $CDCl_3$)** δ 8.10 (1H, d, *J* 8.1 Hz, H8), 7.74 (1H, s, H2), 7.51 (1H, dd, *J* 8.1 and 2.6 Hz, H7), 7.35 (1H, d, *J* 2.6 Hz, H5), 5.75 (1H, br s,

C*H*(OCH_3)), 5.64-5.56 (1H, m, C*H*=CH_2), 5.12-5.06 (2H, m, CH=CH_2), 4.17-4.05 (1H, m, 1 × H7'); 4.07 (3H, s, ArOCH_3), 3.60 (1H, dd *J* 13.6 and 10.6 Hz, 1 × H6'), 3.47 (3H, s, CH(OCH_3)), 3.37-3.30 (2H, m, H2' and 1 × H7'), 3.24-3.19 (1H, m, 1 × H6'), 2.79-2.73 (1H, m, H5'), 2.22-2/12 (3H, m, 1 × H3', H4' and 1 × H8'), 1.99-1.90 (1H, m, 1 × H8), 1.55-1.47 (1H, m, 1 × H8'); **^{13}C NMR (100 MHz, $CDCl_3$)** δ 161.7 (Q), 145.0 (Q), 141.5 (Q), 136.8, 132.7, 130.1 (Q), 128.0 (Q), 125.7, 120.7, 119.5 (Q), 117.6, 99.4, 76.5, 59.7, 57.5, 57.2, 55.2, 44.7, 36.9, 26.5, 24.3, 18.9; **IR ν_{max}/cm^{-1} (neat)** 3014, 2949, 2834, 2688, 2234, 1620, 1477, 1238, 1222, 1163, 1025; **HRMS (ESI)** found *m/z* 364.20172 $[M+H]^+$, $C_{22}H_{26}N_3O_2$ requires *m/z* 364.20195.

NMR Spectra of Novel Compounds

2-((2-Methoxyphenyl)(methyl)amino)pyrimidine (S3)

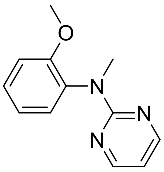

^{1}H NMR (400 MHz, $CDCl_3$)

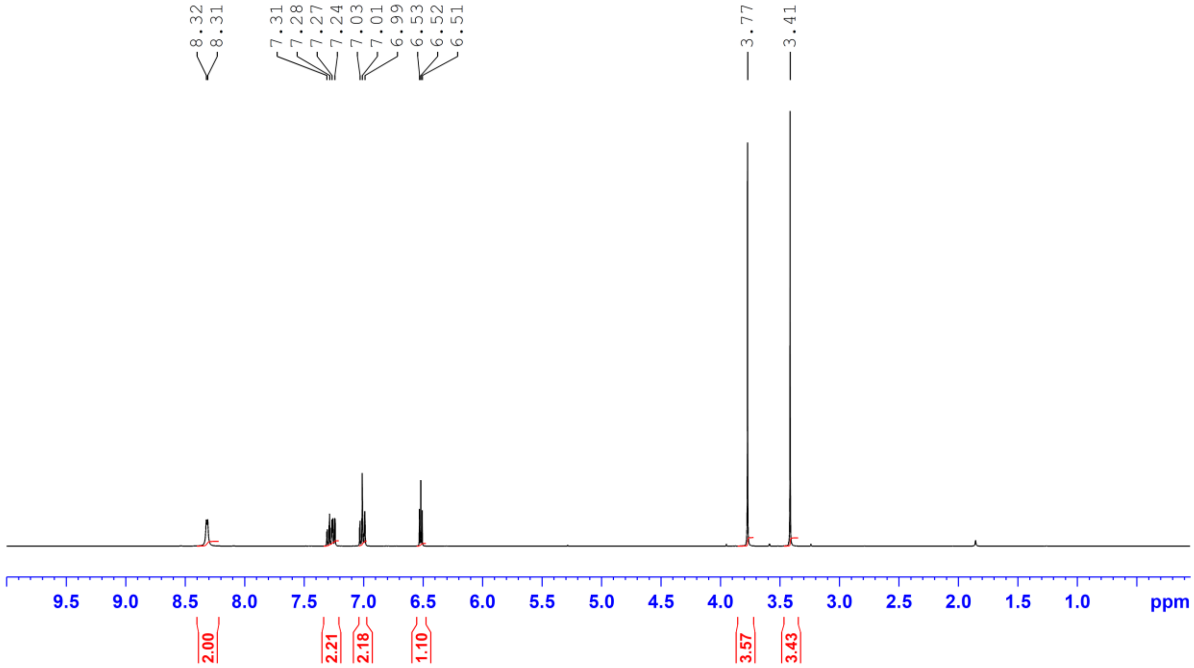

^{13}C NMR (100 MHz, $CDCl_3$)

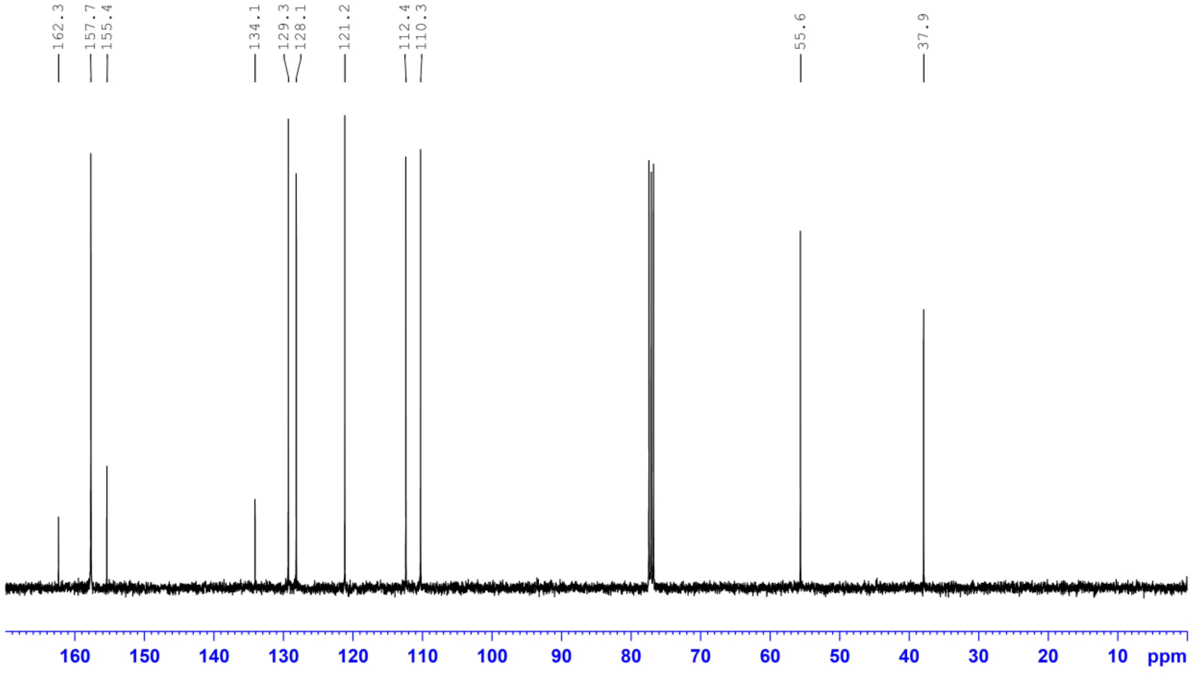

4-(1-Methyl-1*H*-pyrazol-4-yl)pyrimidine (S4)

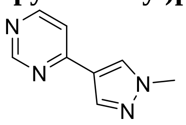

^{1}H NMR (400 MHz, $CDCl_3$)

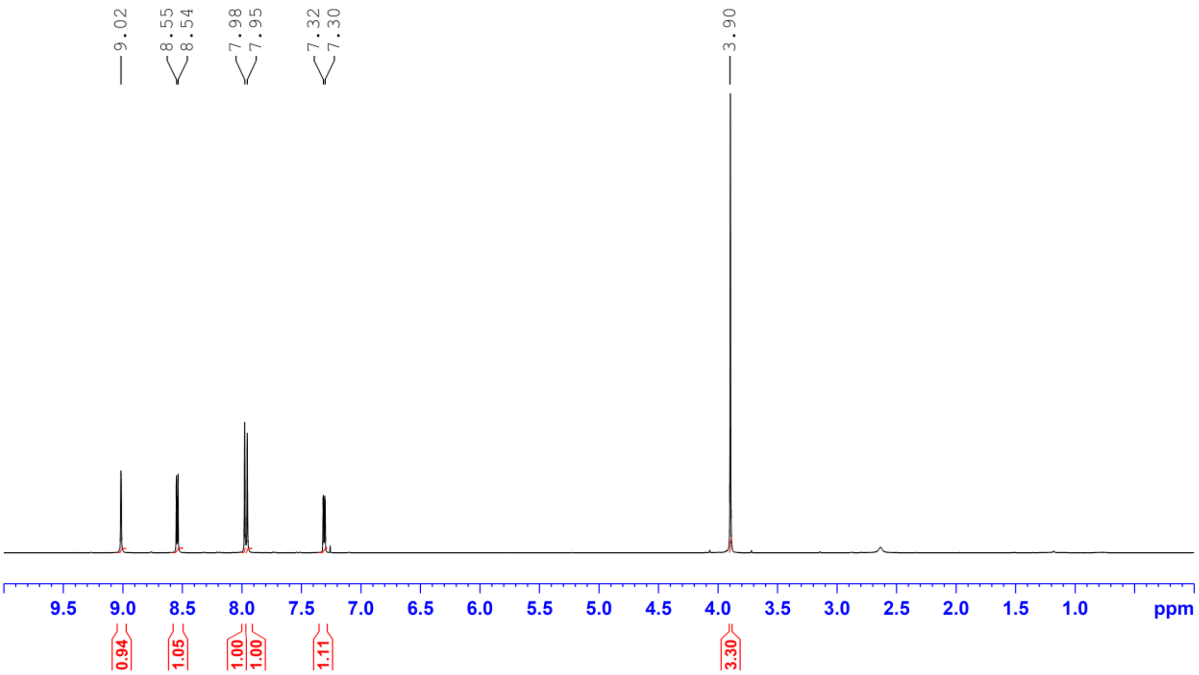

^{13}C NMR (100 MHz, $CDCl_3$)

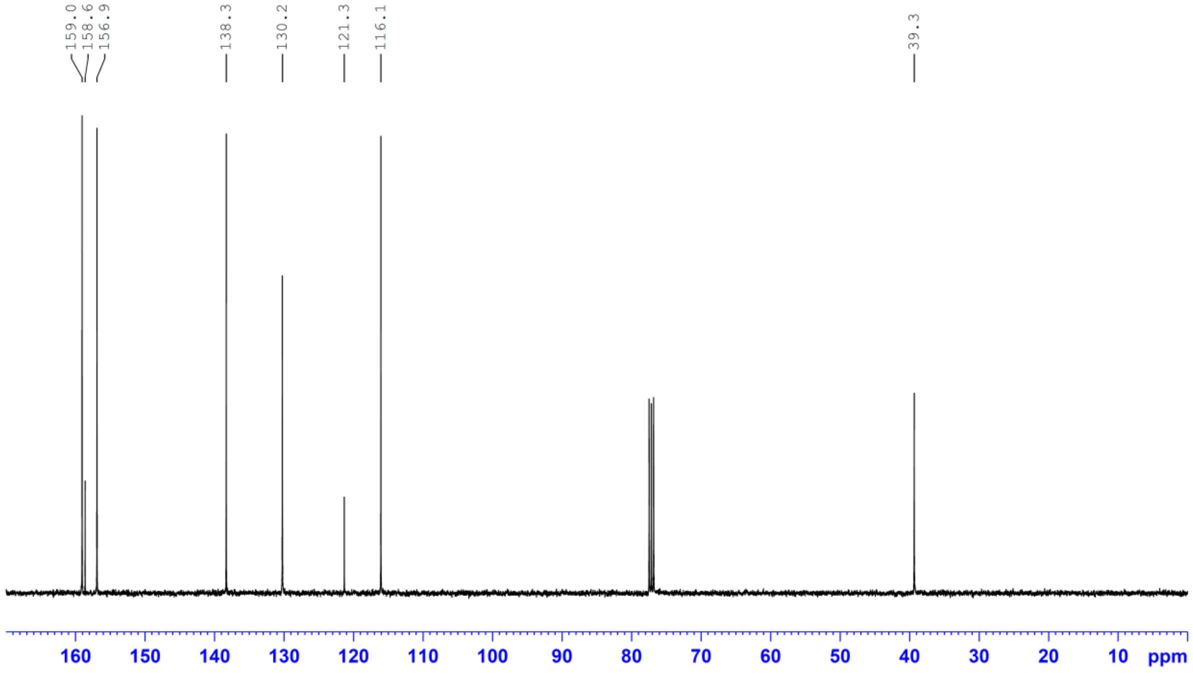

N-(6,7-bis(2-methoxyethoxy)quinazolin-4-yl)-N-(3-ethynylphenyl)acetamide (S5)

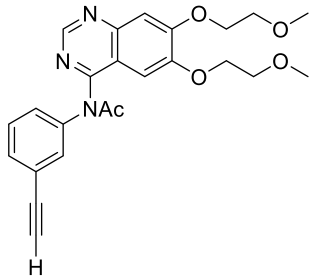

^{1}H NMR (400 MHz, $CDCl_3$)

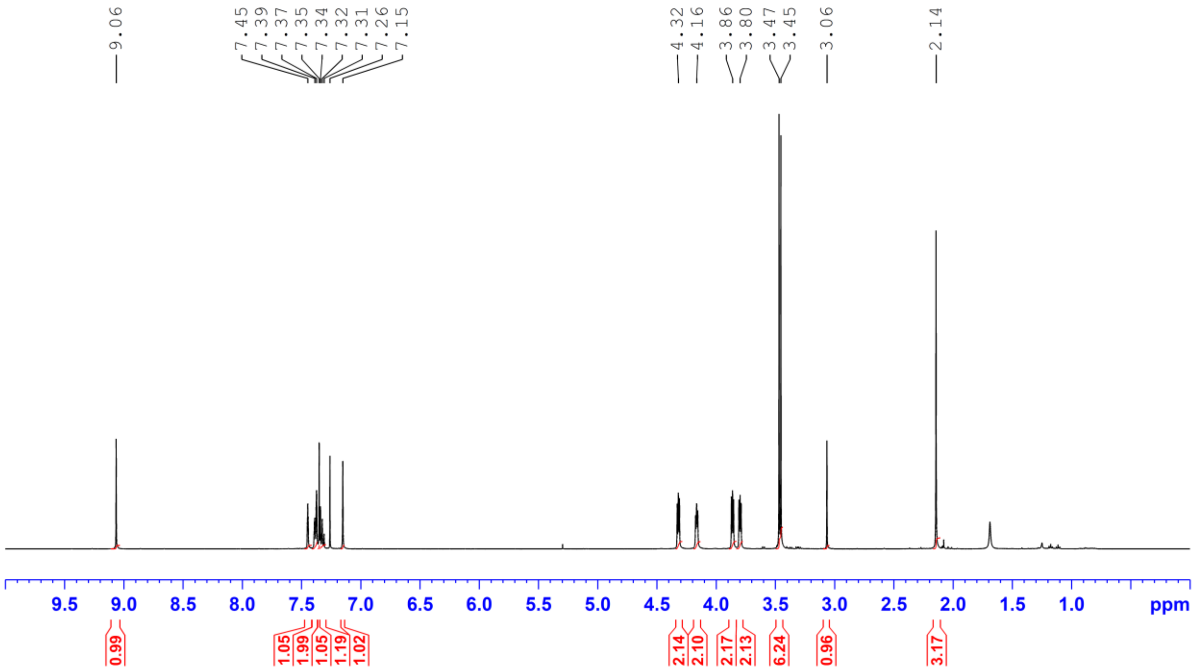

^{13}C NMR (100 MHz, $CDCl_3$)

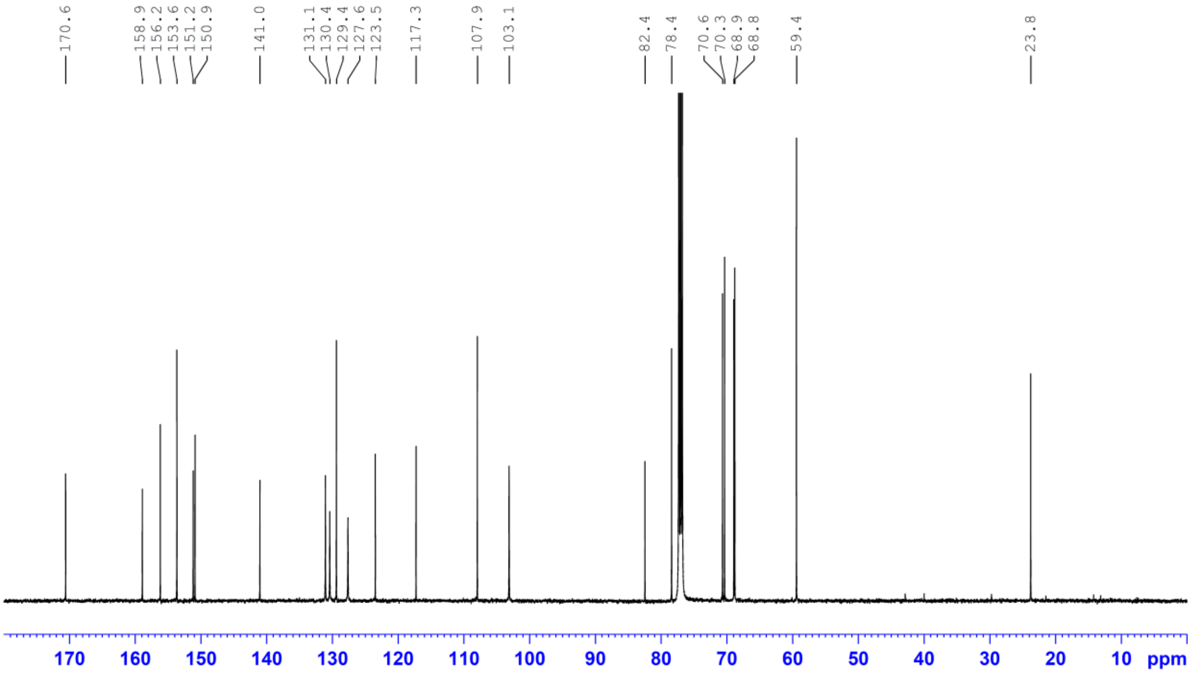

Methyl 4-cyanonicotinate (12) and methyl 2-cyanonicotinate (12'')

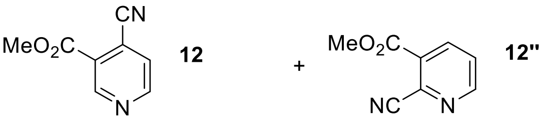

^{1}H NMR (400 MHz, $CDCl_3$)

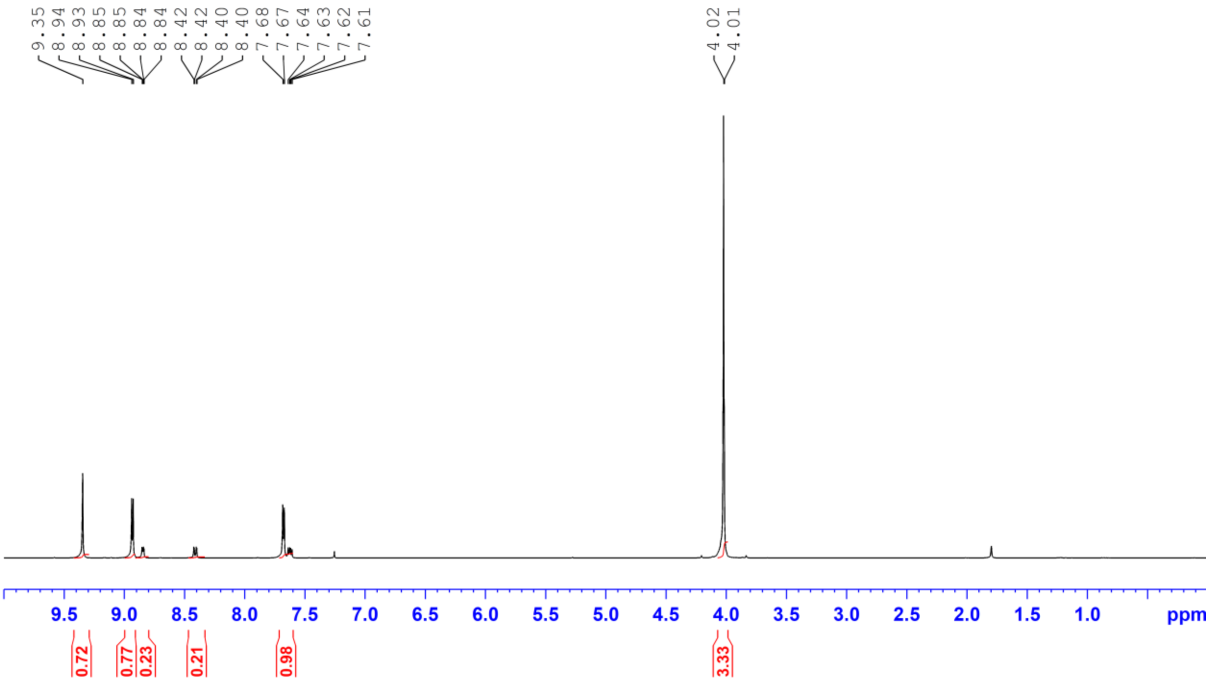

^{13}C NMR (100 MHz, $CDCl_3$)

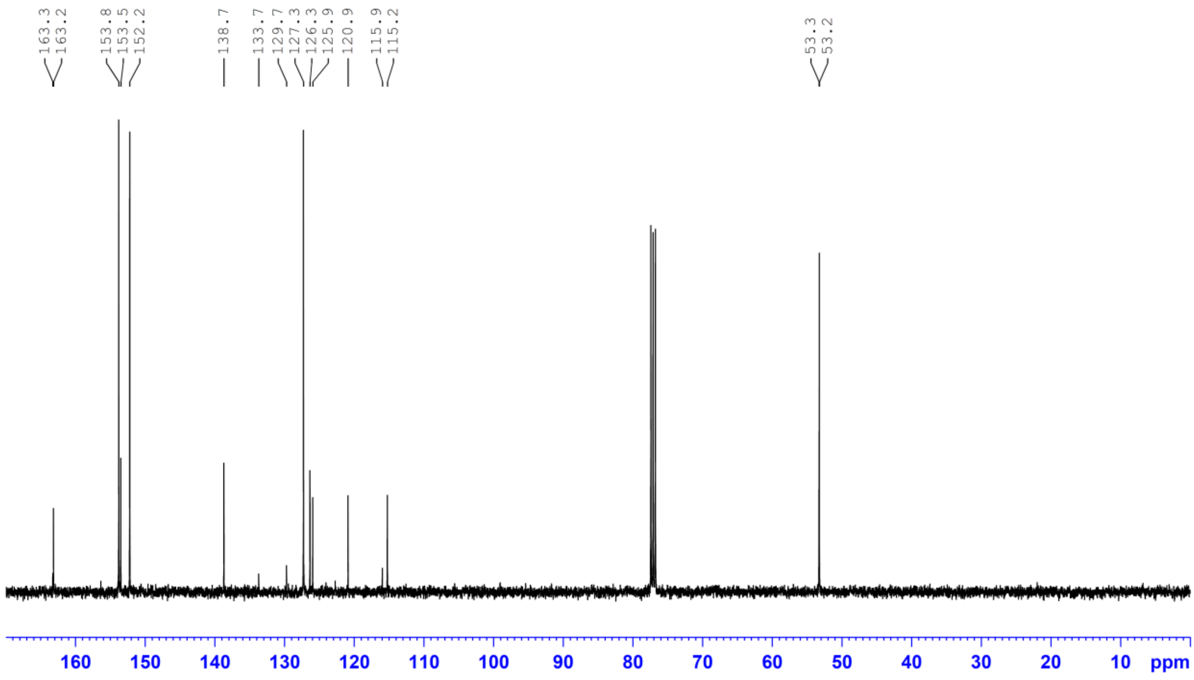

4-Cyanopyridin-3-yl acetate (13), 6-cyanopyridin-3-yl acetate (13’) and 2-cyanopyridin-3-yl acetate (13’’)

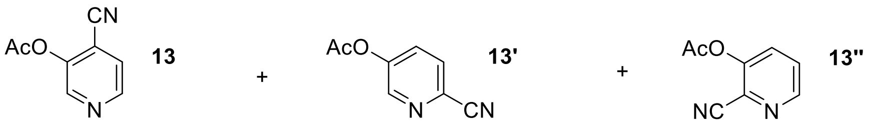

^{1}H NMR (400 MHz, $CDCl_3$)

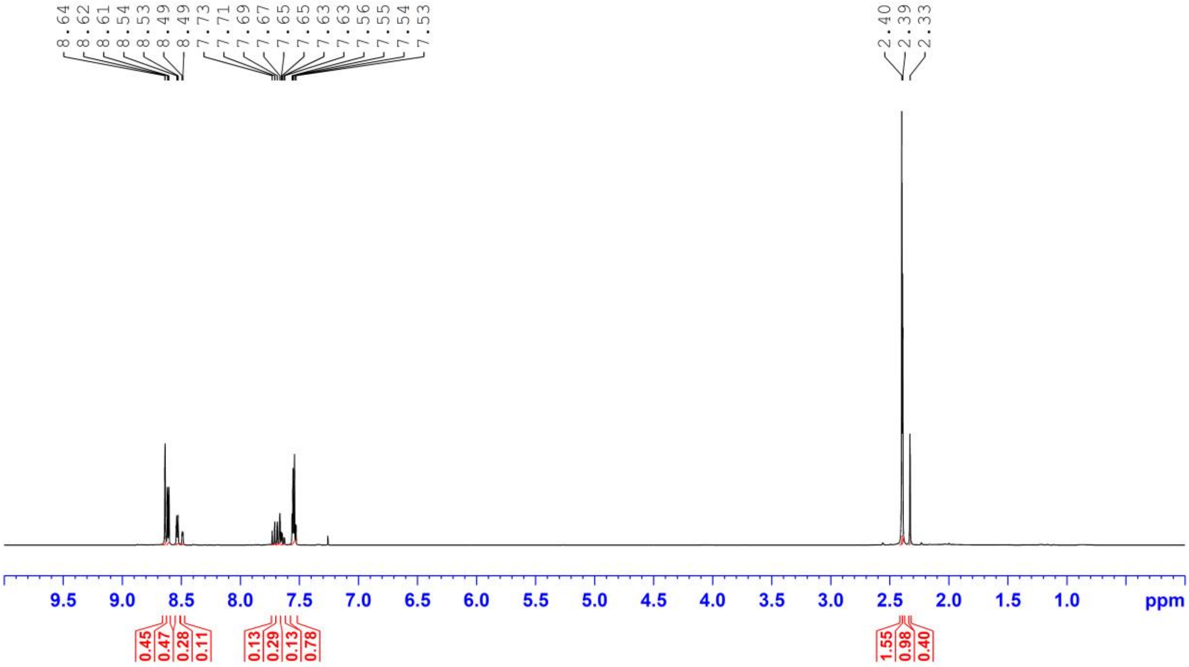

^{13}C NMR (100 MHz, $CDCl_3$)

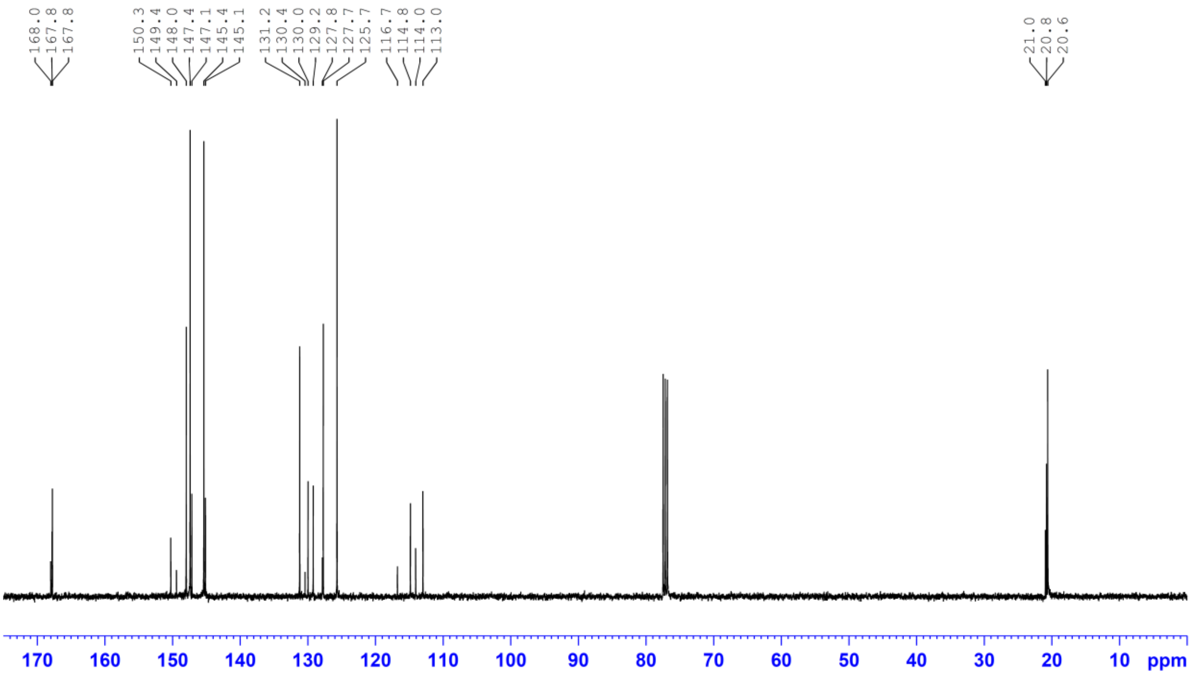

6,7-Dihydro-5*H*-cyclopenta[*b*]pyridine-4-carbonitrile (17)

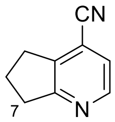

17

^{1}H NMR (400 MHz, $CDCl_3$)

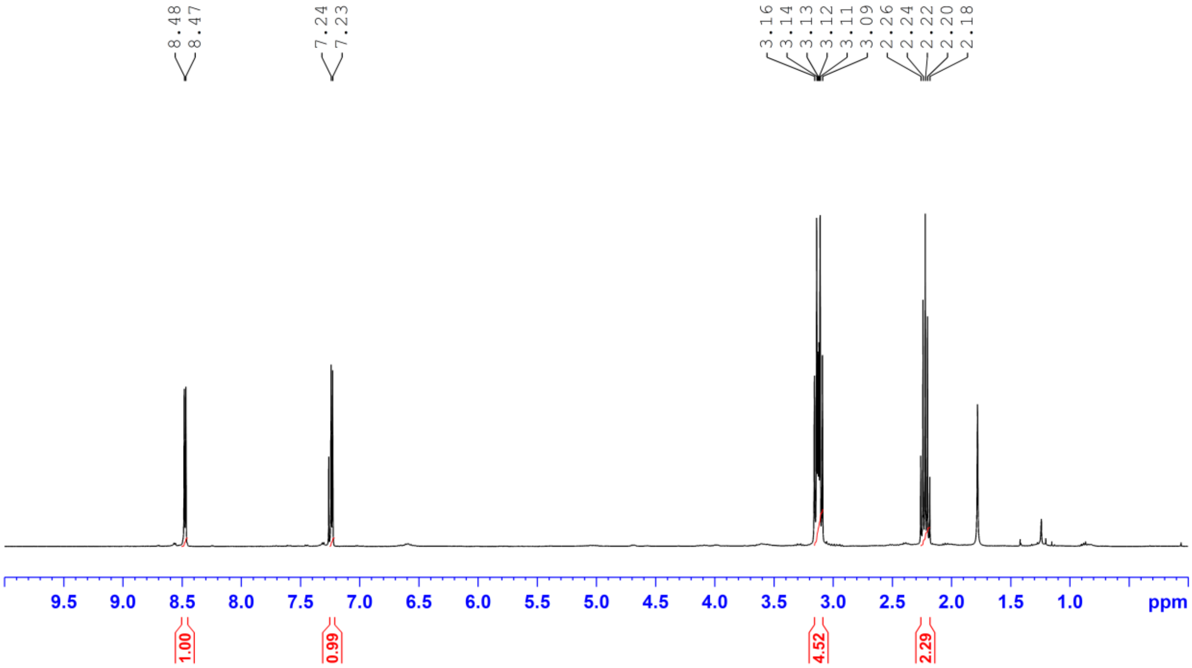

^{13}C NMR (100 MHz, $CDCl_3$)

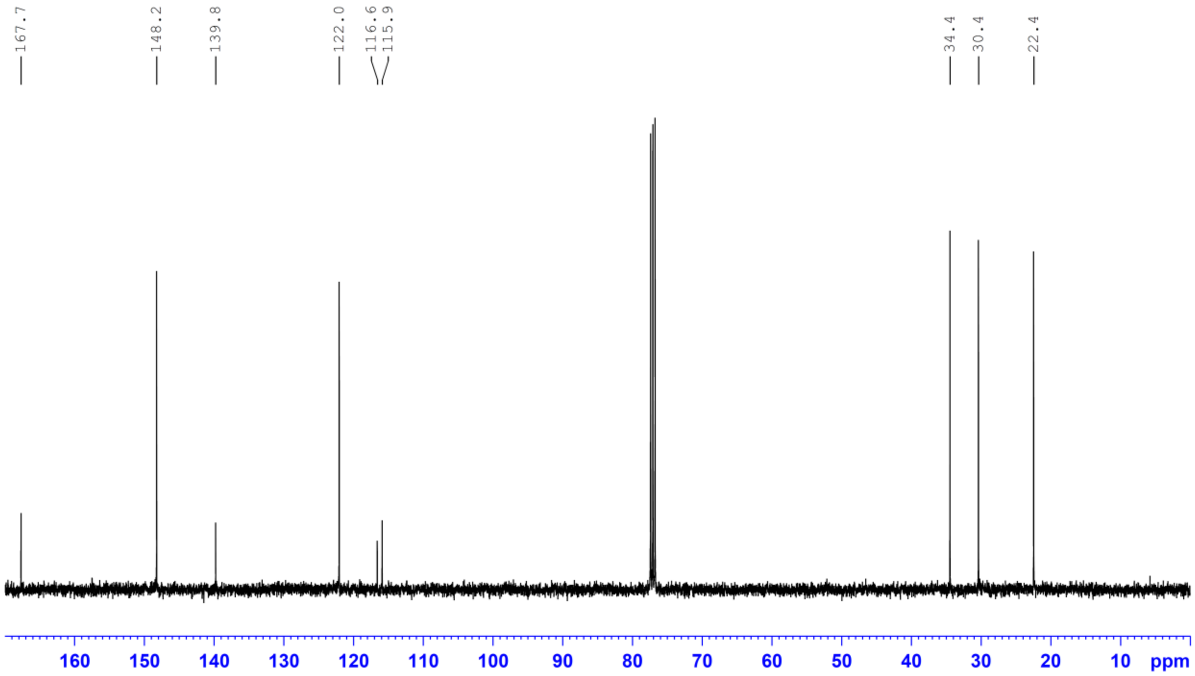

3-Hexylisonicotinonitrile (19)

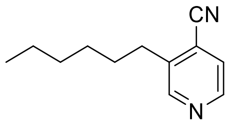

19

^{1}H NMR (400 MHz, $CDCl_3$)

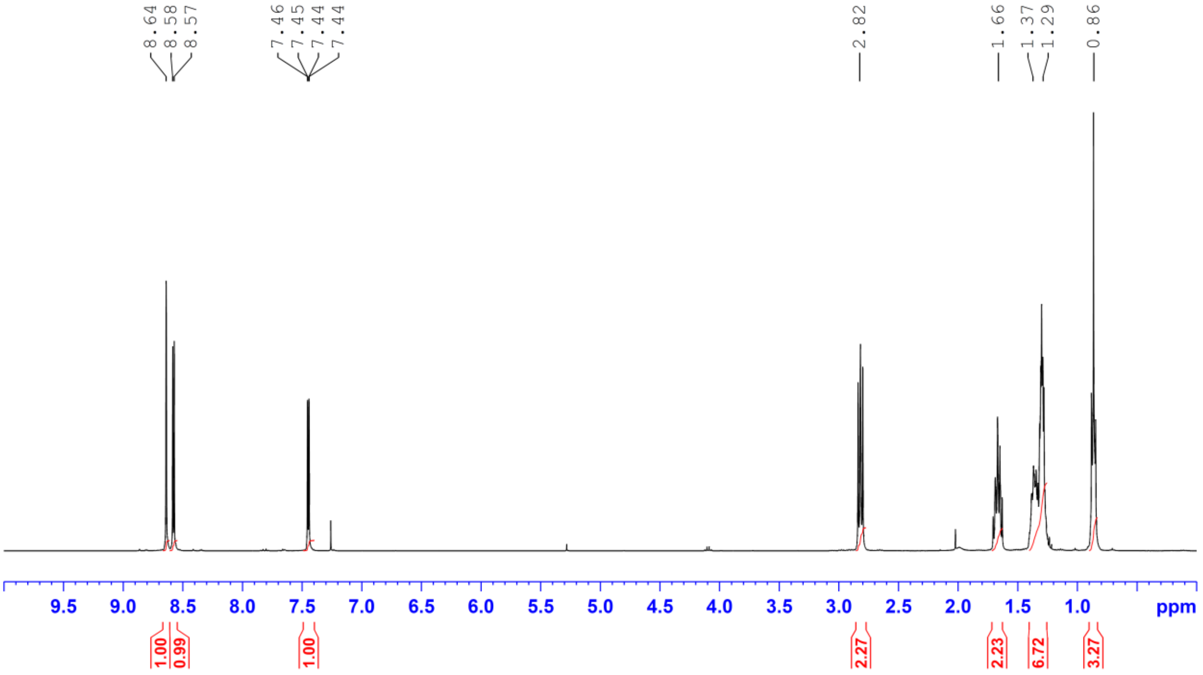

^{13}C NMR (100 MHz, $CDCl_3$)

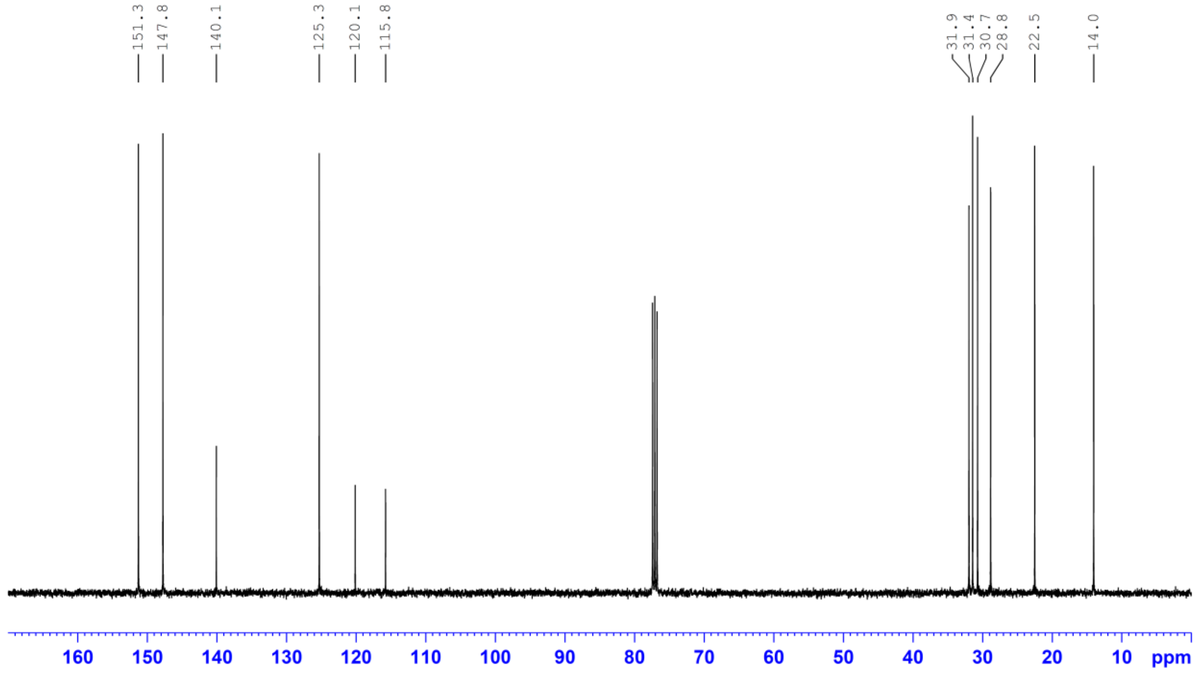

3-Bromoisoquinoline-1-carbonitrile (24)

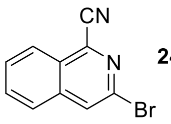

24

^{1}H NMR (400 MHz, $CDCl_3$)

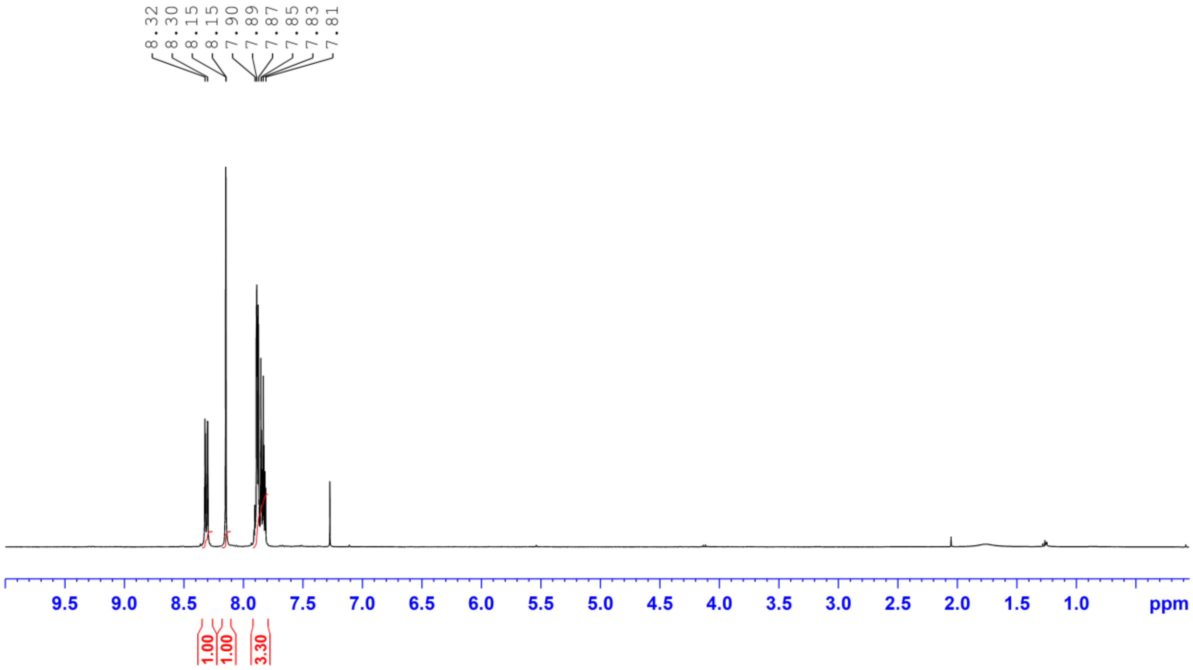

^{13}C NMR (100 MHz, $CDCl_3$)

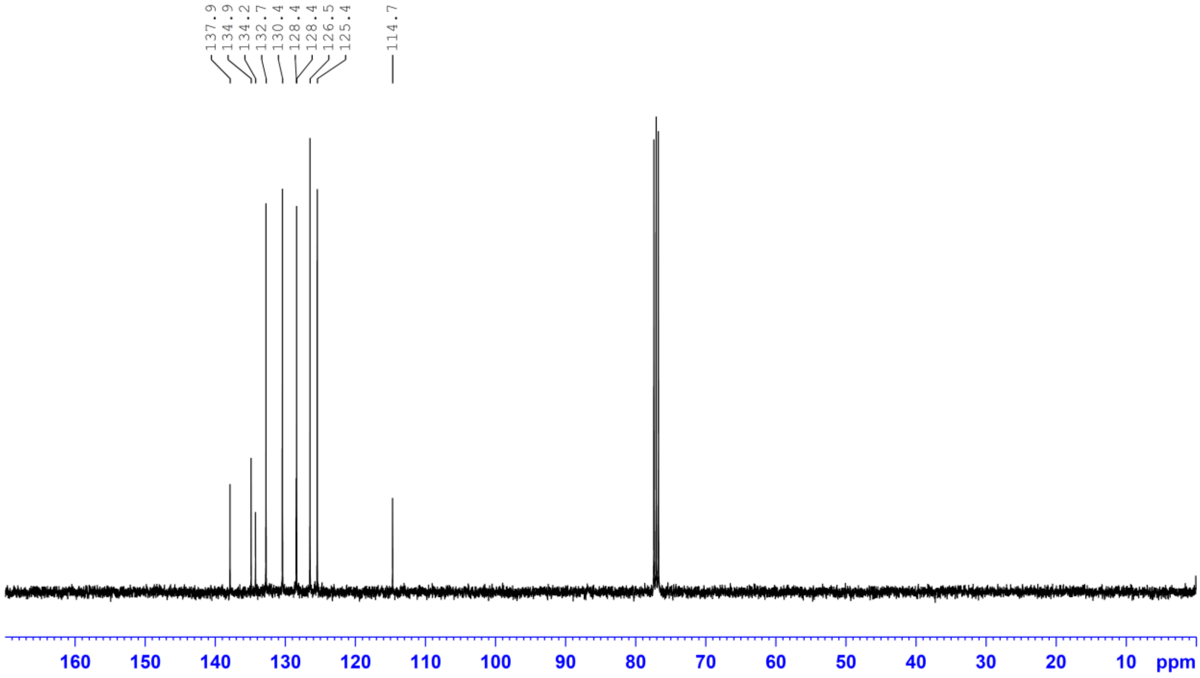

Methyl 3-cyanopyridazine-4-carboxylate (27)

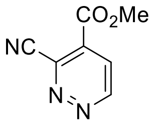

27

^{1}H NMR (500 MHz, $CDCl_3$)

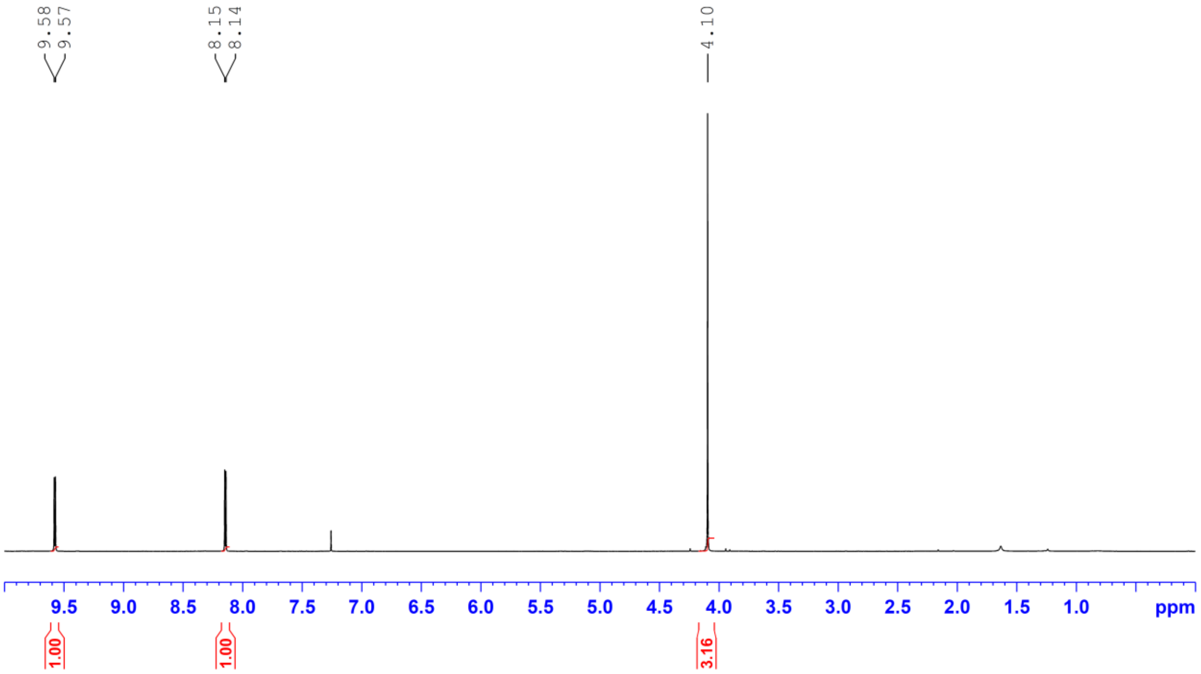

^{13}C NMR (125 MHz, $CDCl_3$)

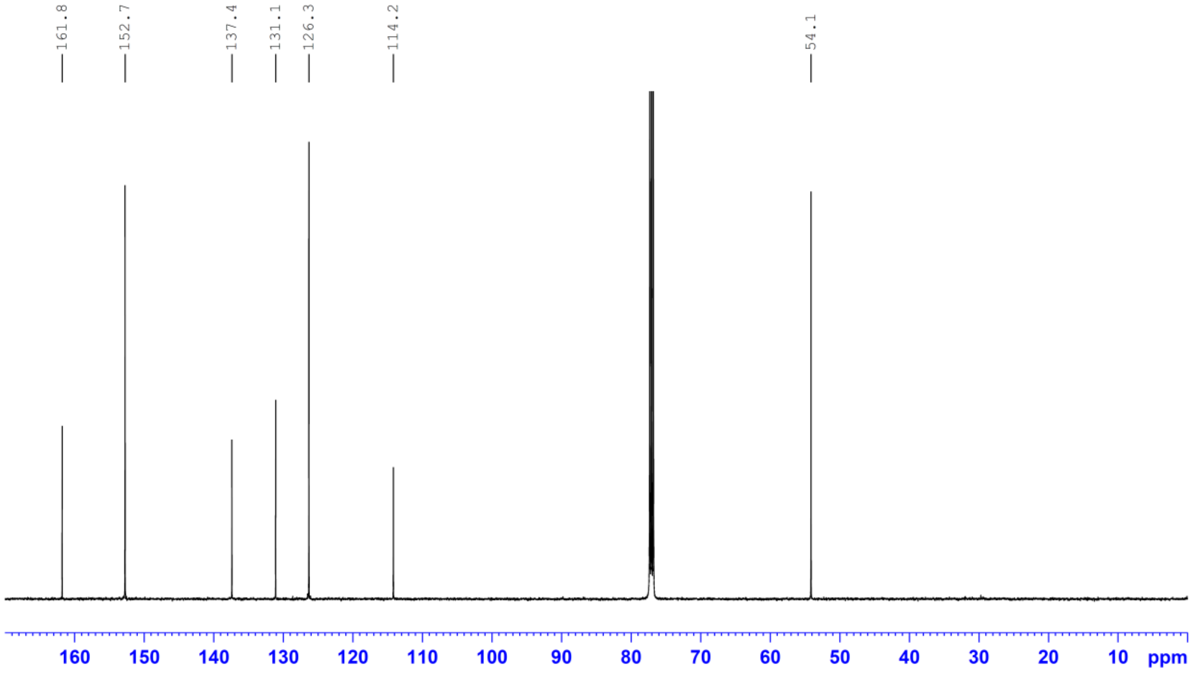

4-Phenylpyrimidine-2-carbonitrile (28)

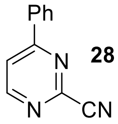

^{1}H NMR (400 MHz, $CDCl_3$)

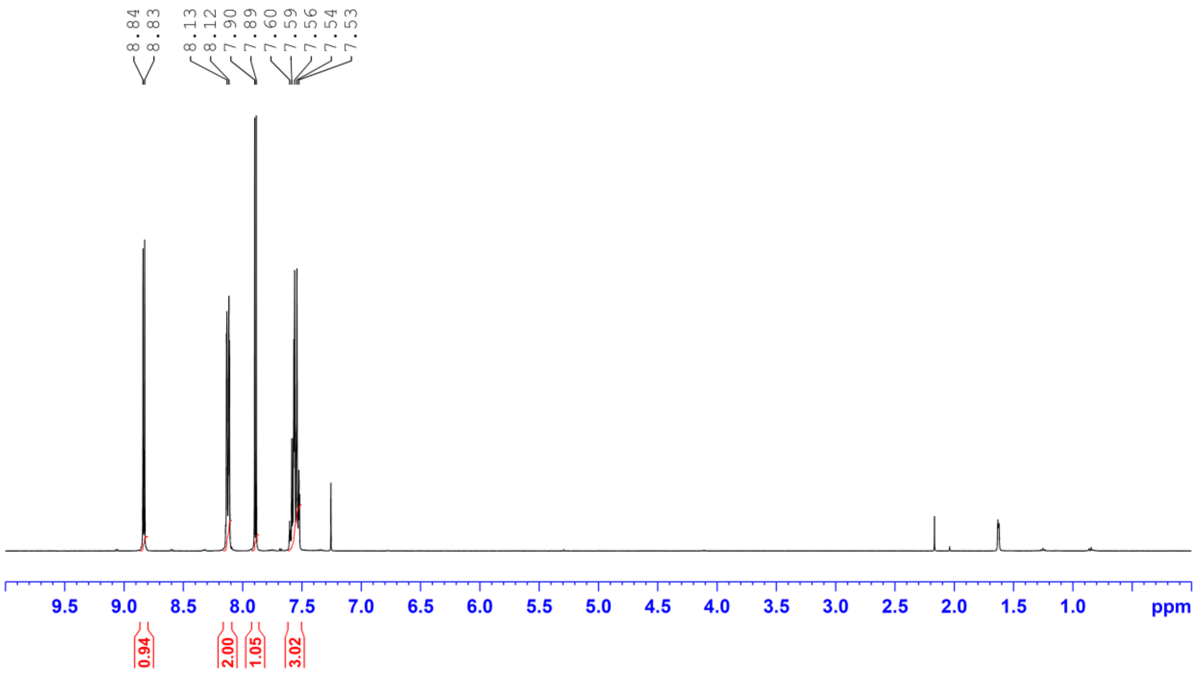

^{13}C NMR (100 MHz, $CDCl_3$)

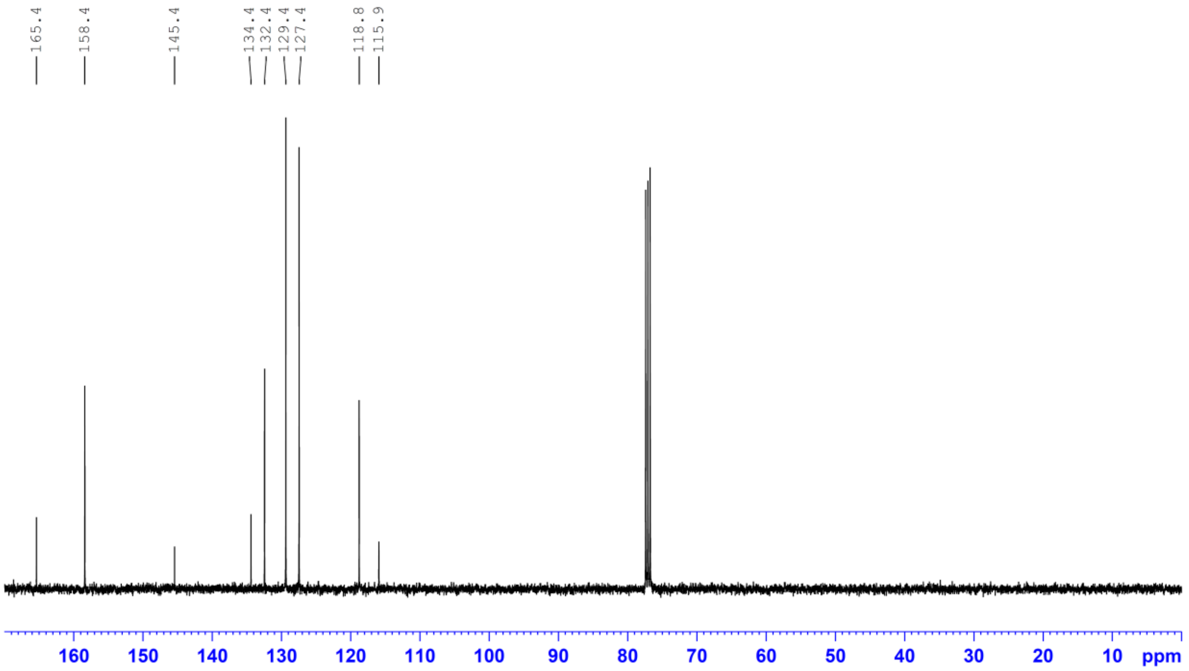

5-Phenylpyrimidine-2,4-dicarbonitrile (30)

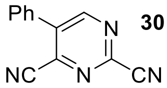

30

^{1}H NMR (400 MHz, $CDCl_3$)

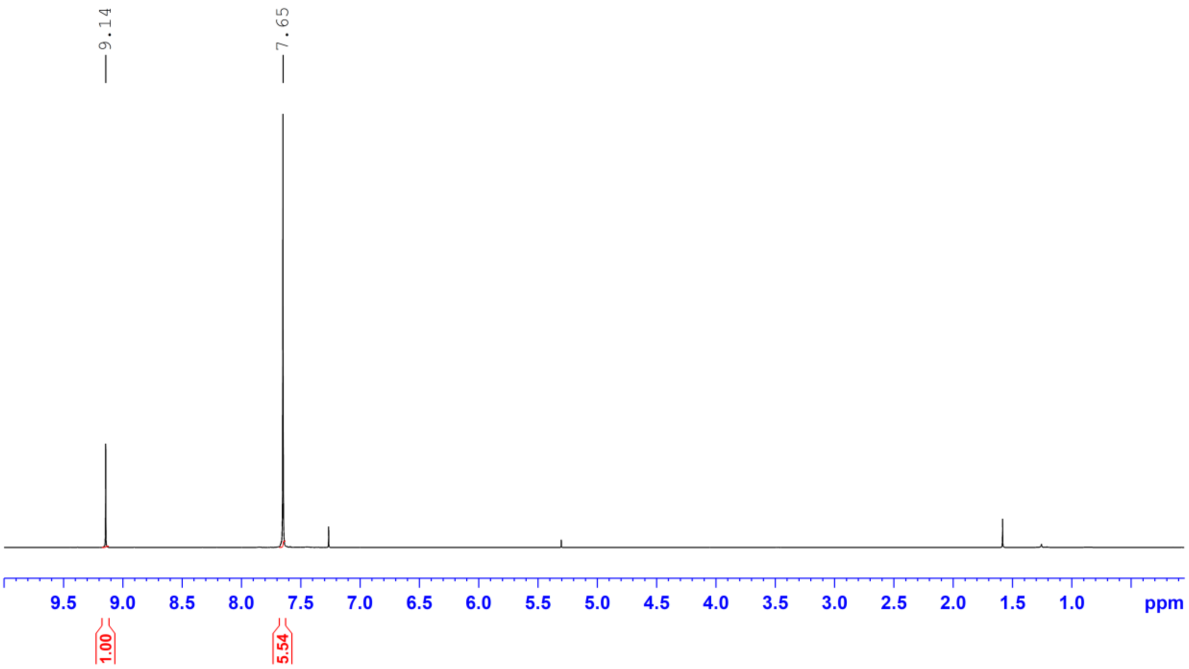

^{13}C NMR (100 MHz, $CDCl_3$)

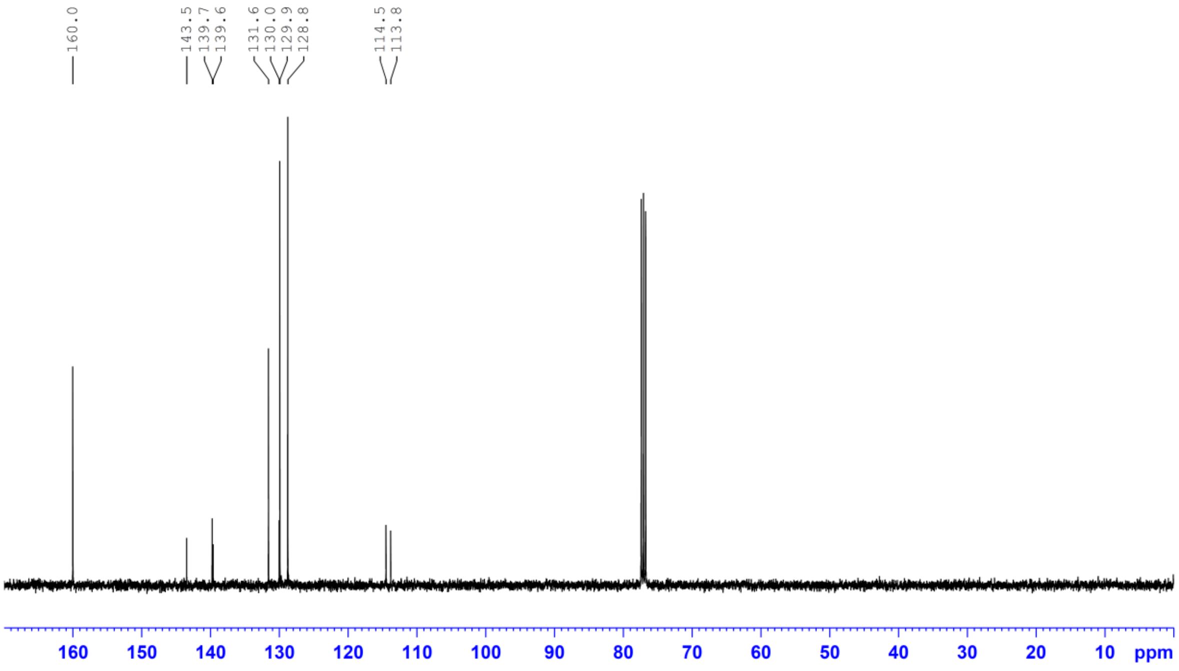

5-(Trifluoromethyl)pyrazine-2-carbonitrile (33)

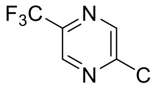

33

^{1}H NMR (400 MHz, $CDCl_3$)

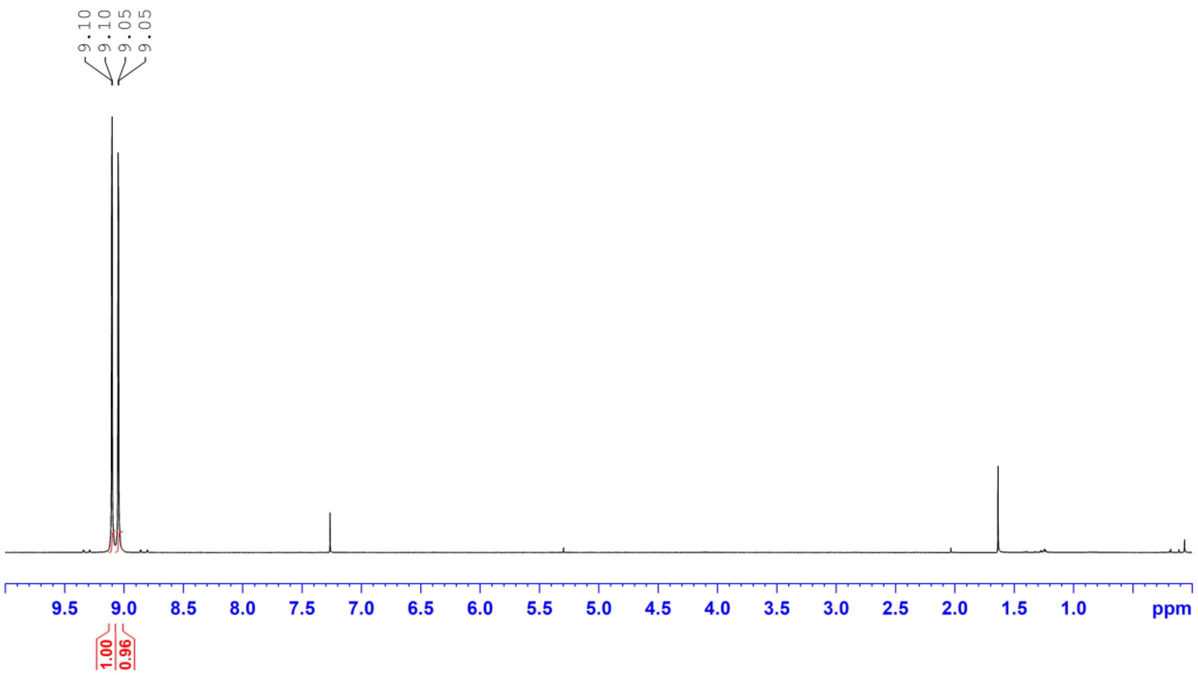

^{13}C NMR (100 MHz, $CDCl_3$)

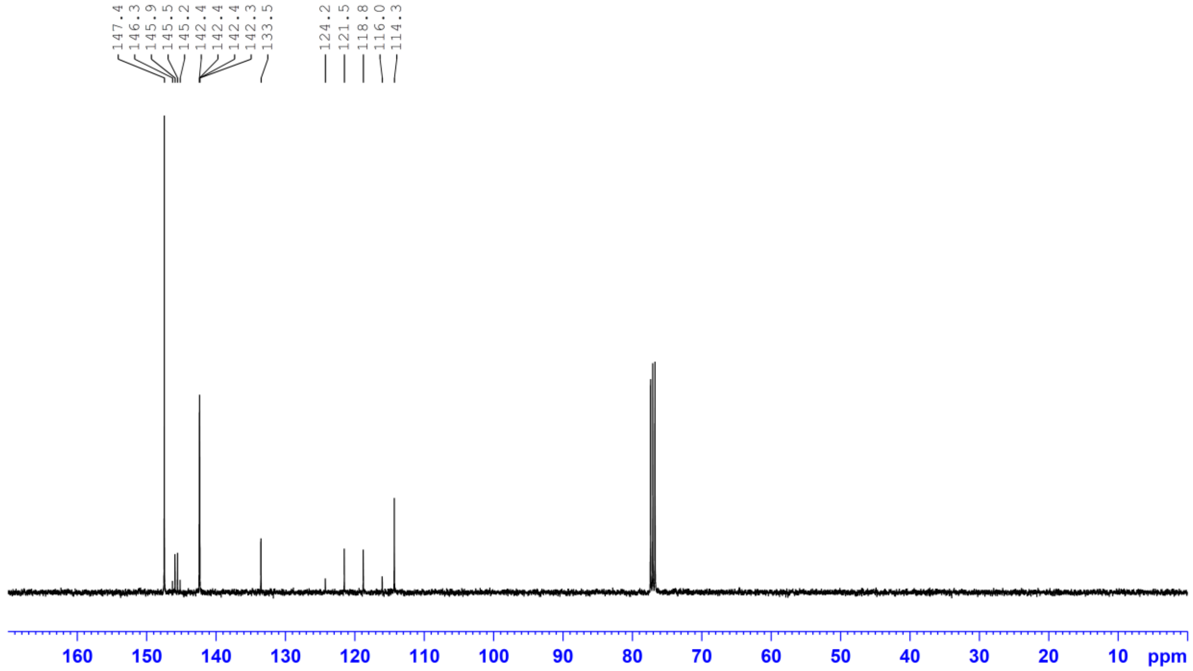

4,4'-Di-*tert*-butyl-[2,2'-bipyridine]-6-carbonitrile (37)

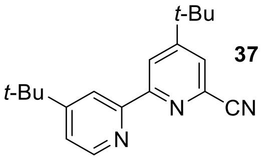

^{1}H NMR (400 MHz, $CDCl_3$)

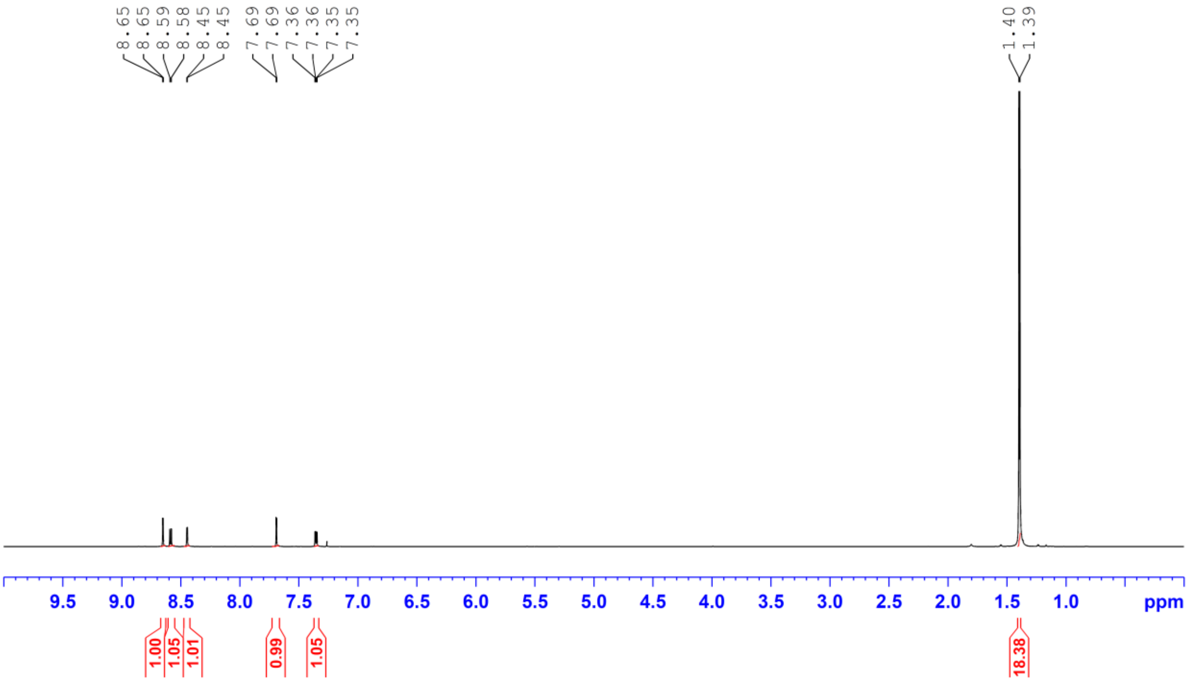

^{13}C NMR (100 MHz, $CDCl_3$)

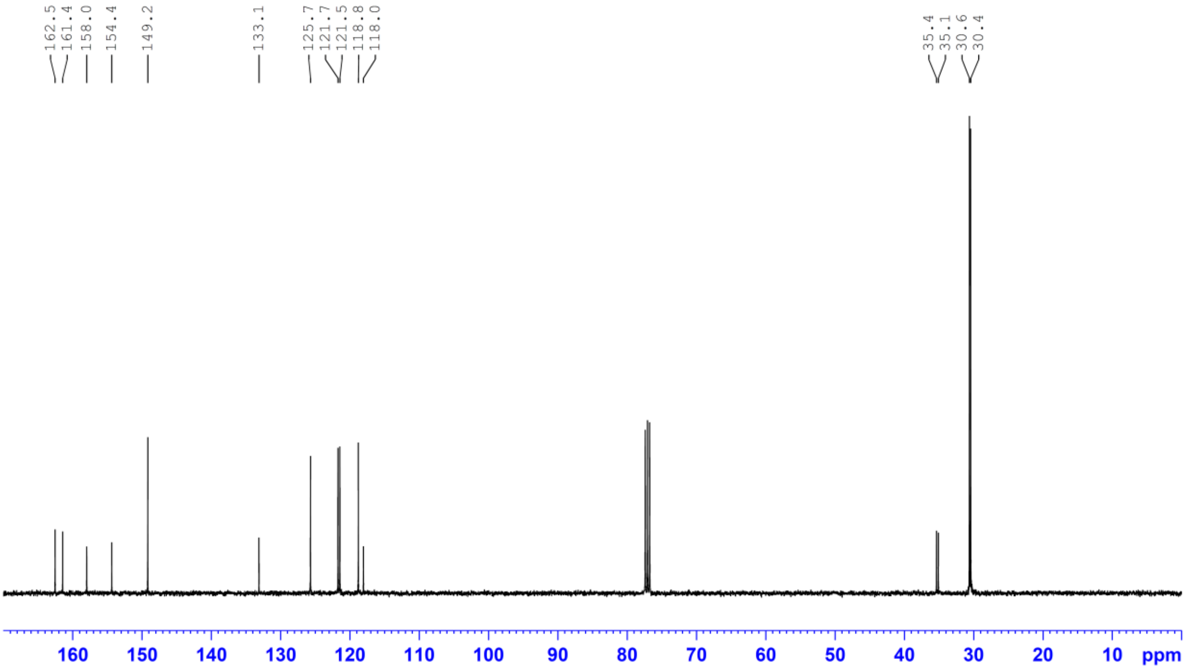

Imidazo[1,2-*a*]pyrazine-8-carbonitrile (39)

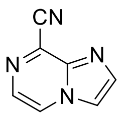

39

^{1}H NMR (500 MHz, DMSO-d_6)

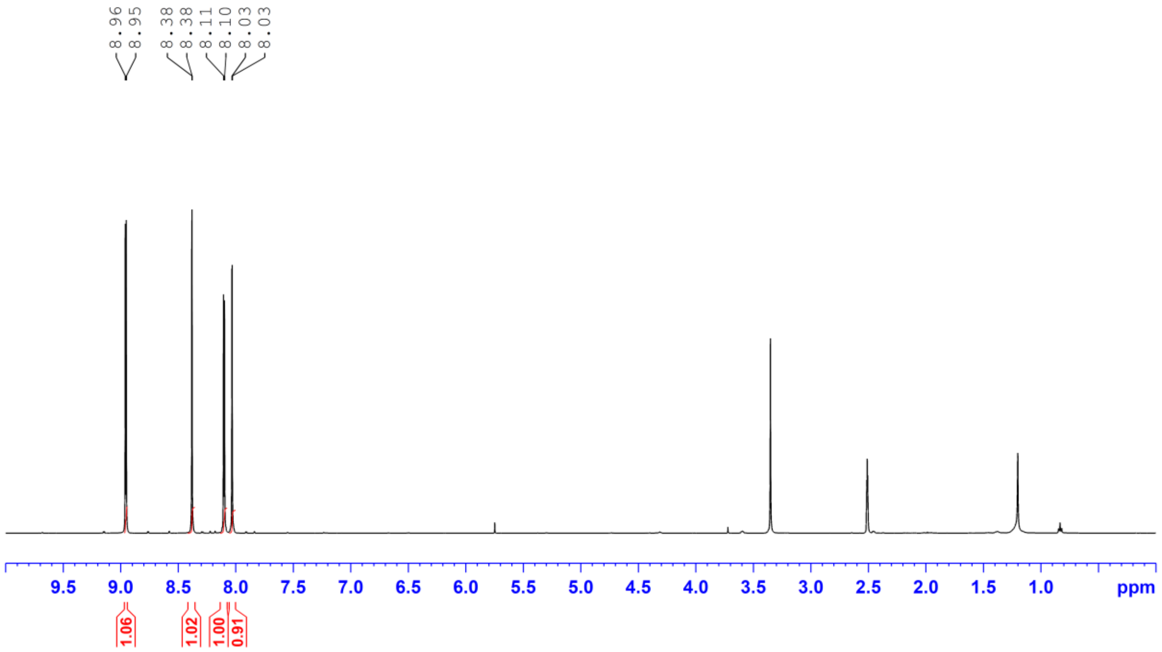

^{13}C NMR (125 MHz, DMSO-d_6)

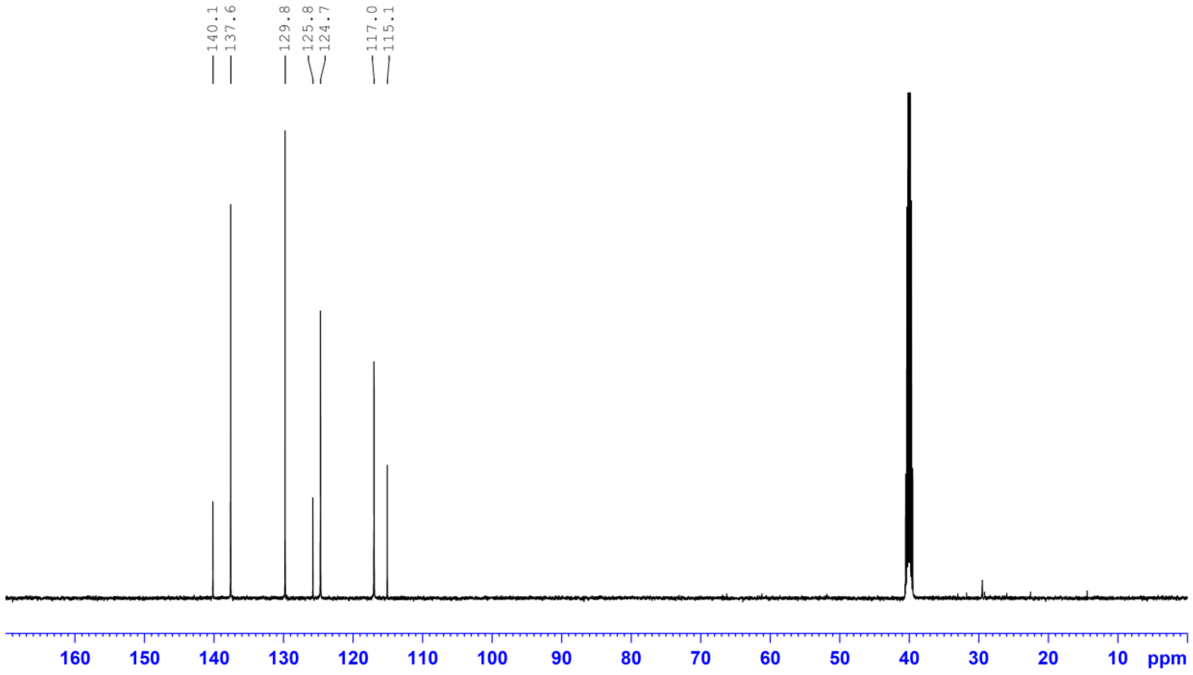

3-Bromoimidazo[1,2-a]pyrazine-8-carbonitrile (40)

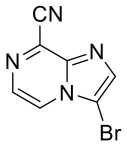

40

^{1}H NMR (400 MHz, $CDCl_3$)

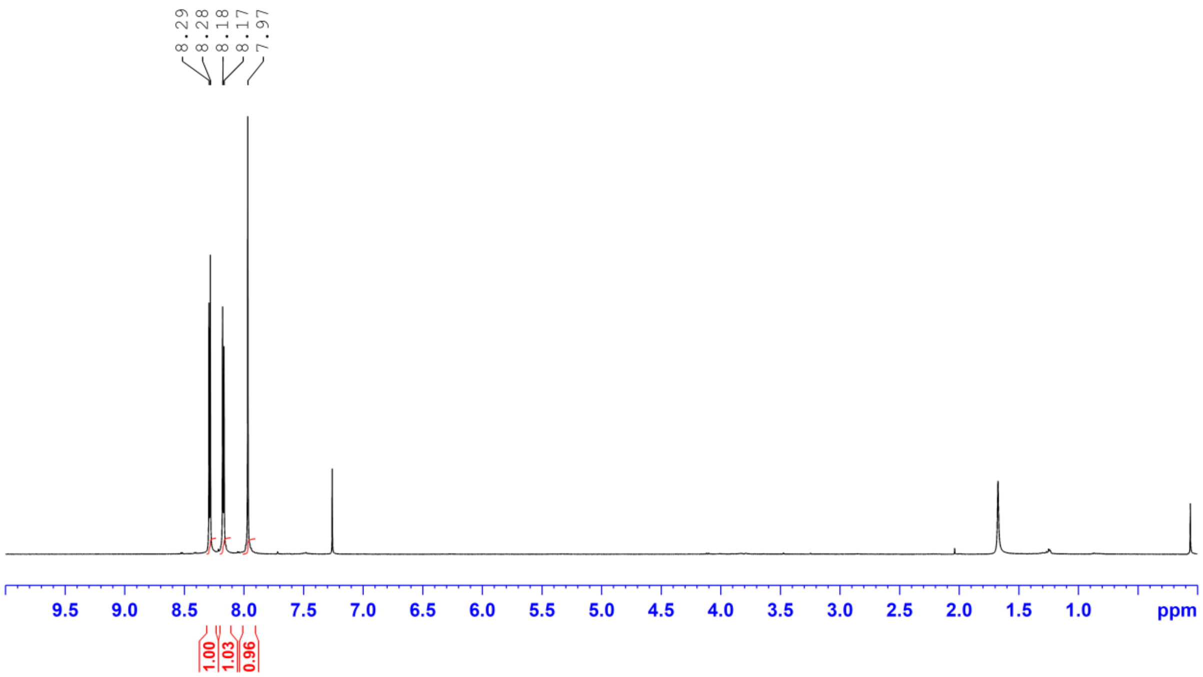

^{13}C NMR (100 MHz, $CDCl_3$)

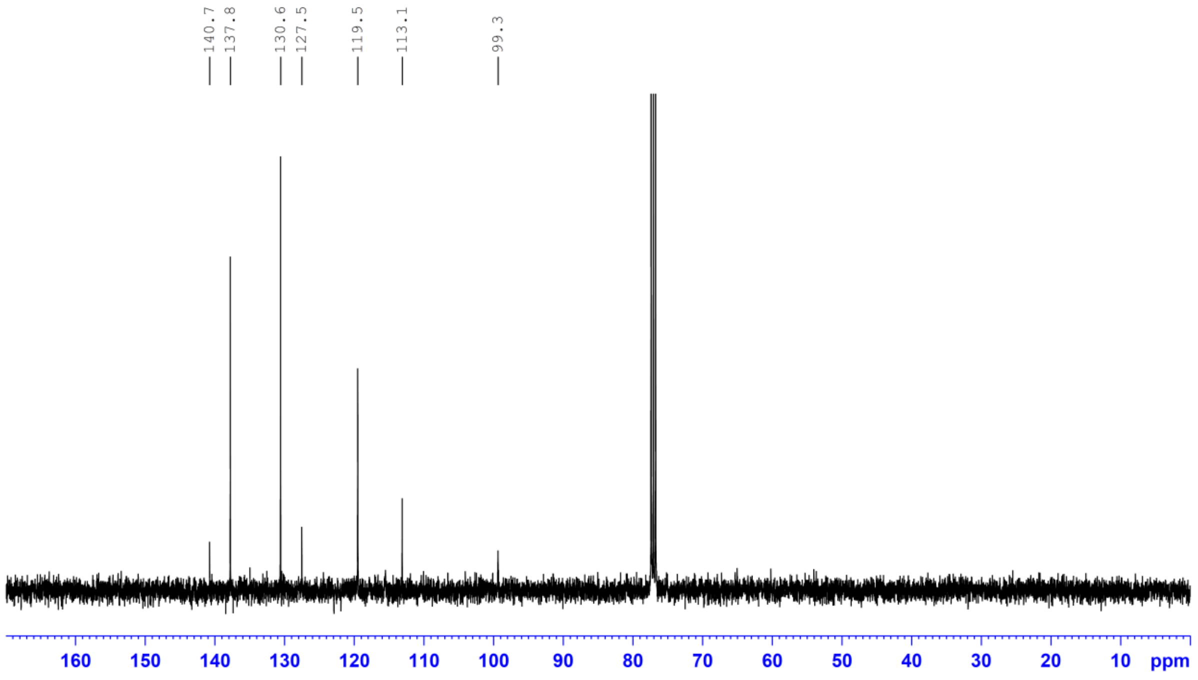

2-(Trifluoromethyl)imidazo[1,2-a]pyrazine-3-carbonitrile (41)

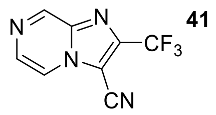

^{1}H NMR (500 MHz, $CDCl_3$)

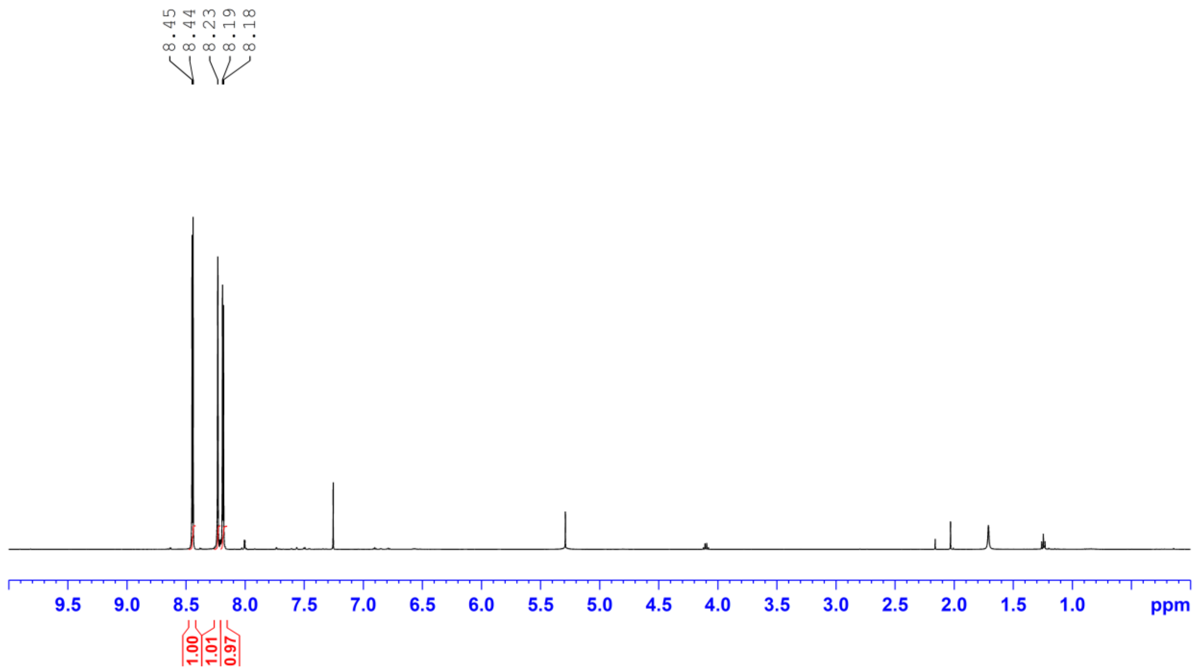

^{13}C NMR (125 MHz, $CDCl_3$)

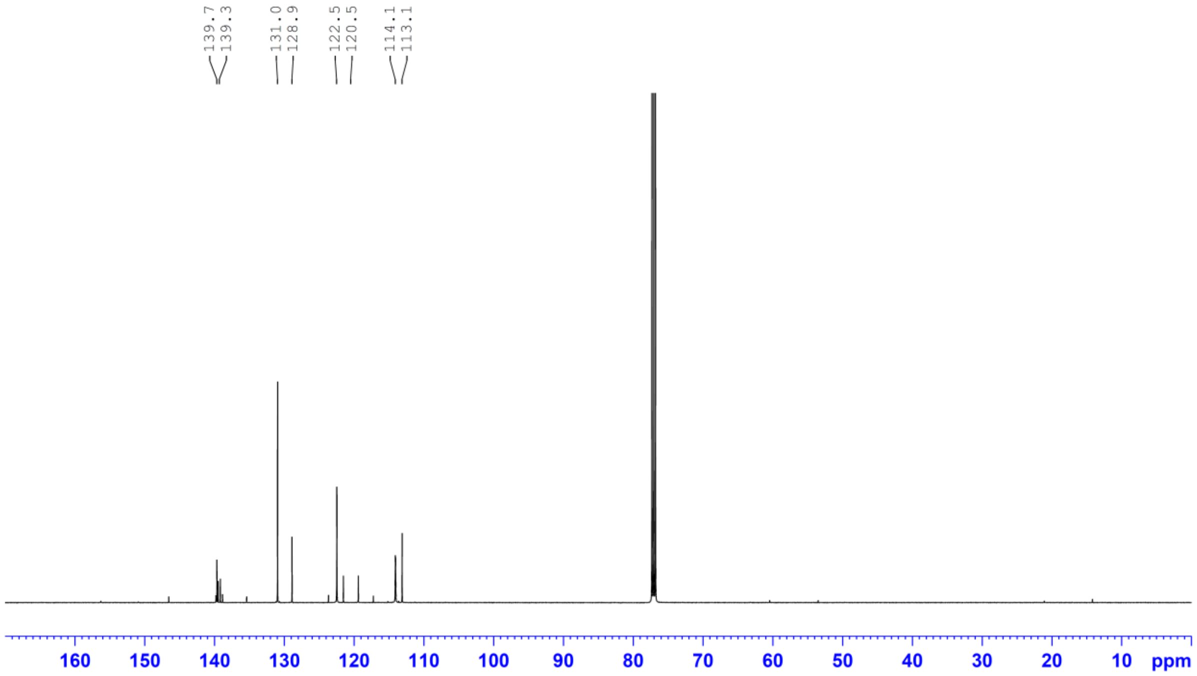

3-Bromopyrazolo[1,5-*a*]pyrimidine-7-carbonitrile (42)

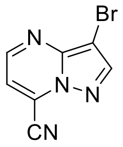

42

^{1}H NMR (400 MHz, $CDCl_3$)

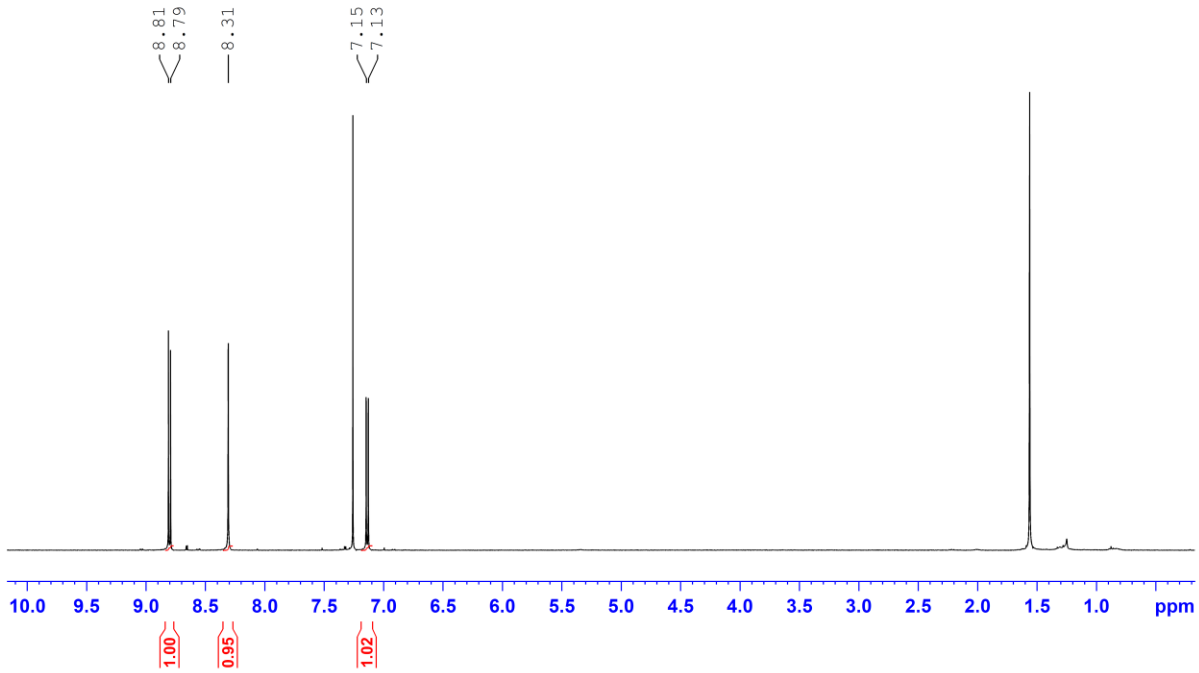

^{13}C NMR (125 MHz, $CDCl_3$)

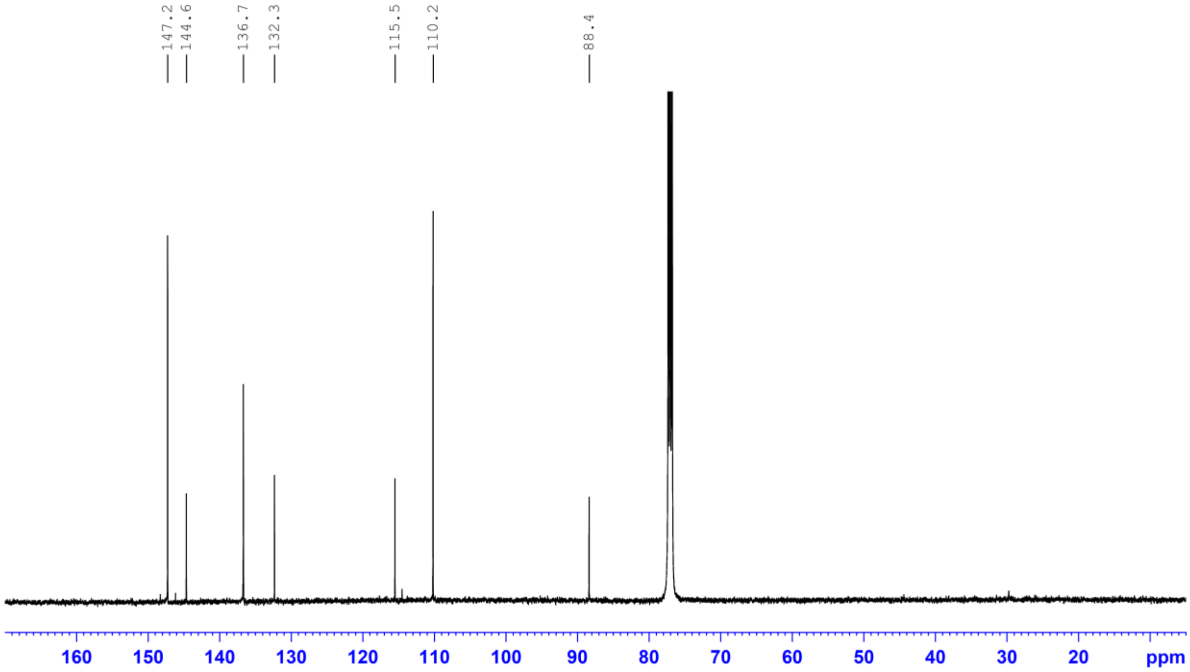

3-Bromopyrazolo[1,5-*a*]pyrimidine-5-carbonitrile (42')

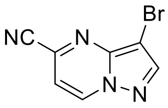

42'

^{1}H NMR (400 MHz, $CDCl_3$)

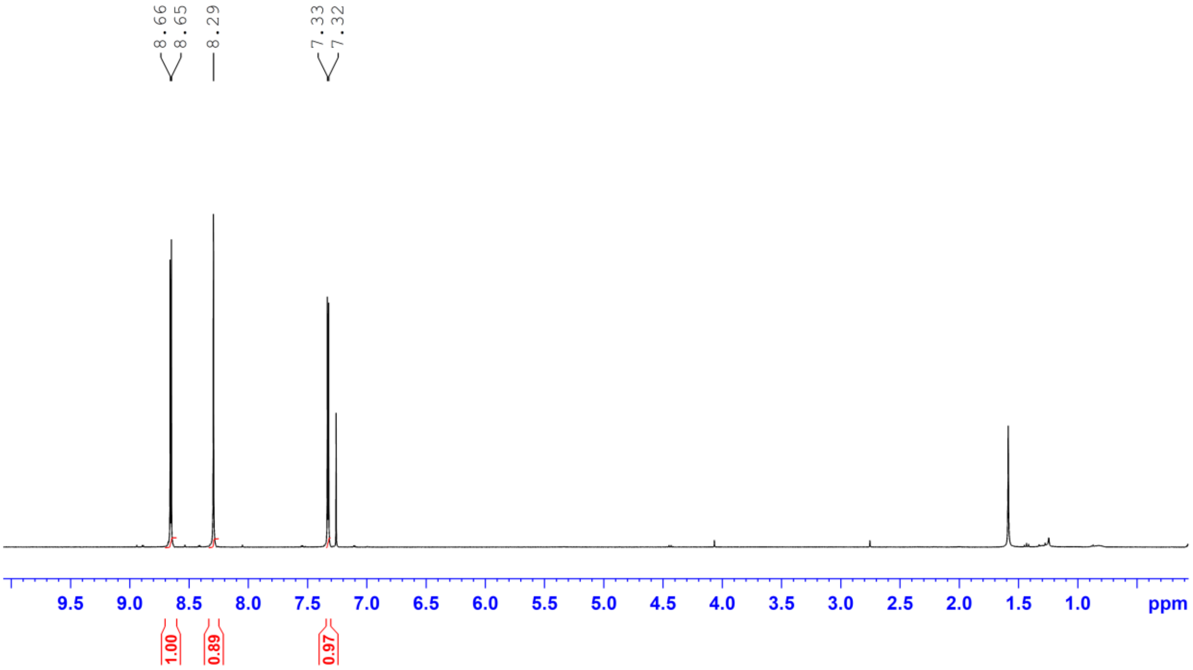

^{13}C NMR (125 MHz, $CDCl_3$)

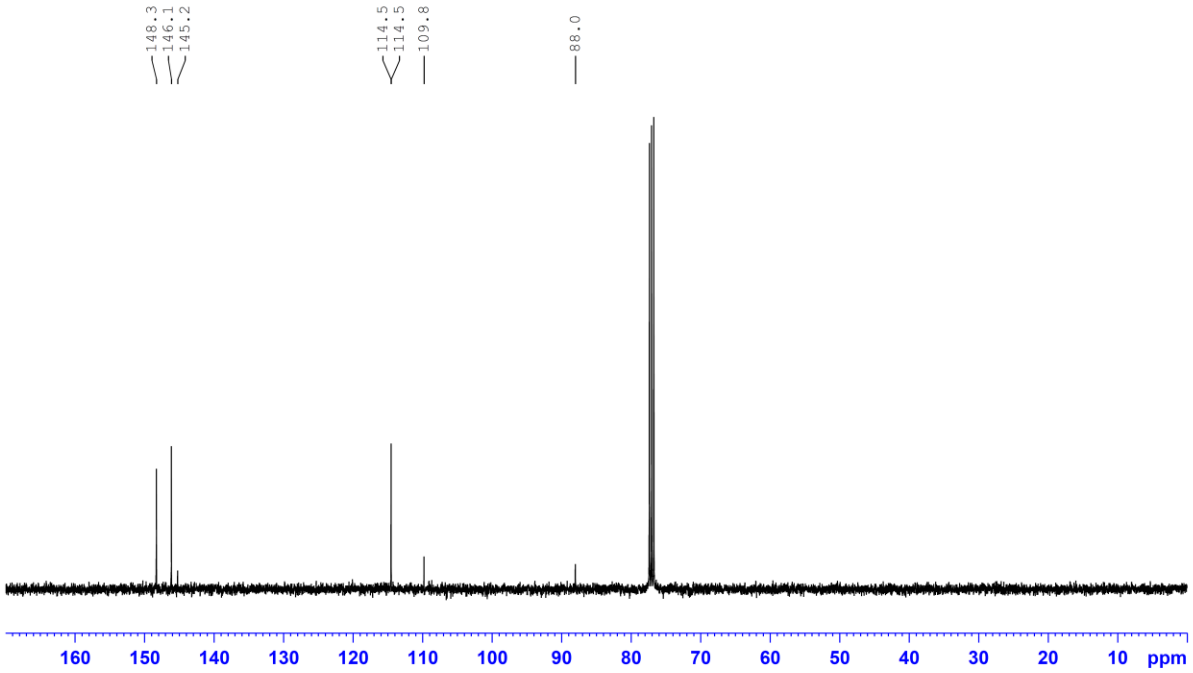

1-Methyl-1*H*-pyrazolo[3,4-*b*]pyridine-4-carbonitrile (43)

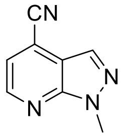

43

^{1}H NMR (500 MHz, $CDCl_3$)

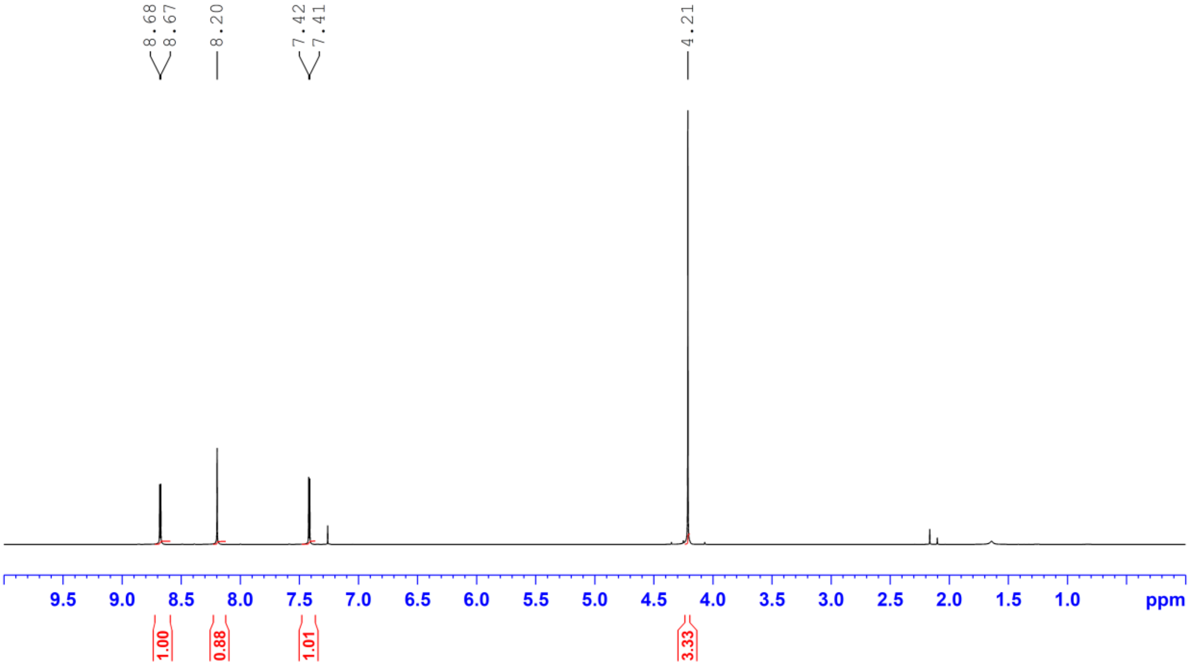

^{13}C NMR (125 MHz, $CDCl_3$)

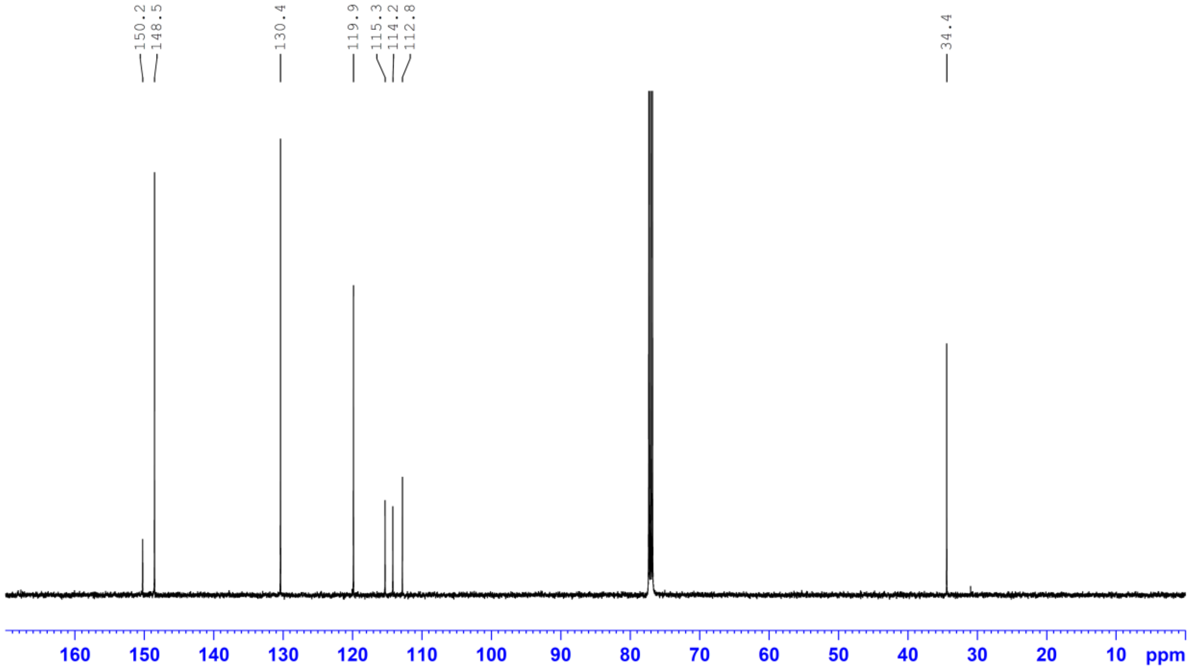

1-Methyl-1*H*-pyrazolo[3,4-*b*]pyridine-4-carbonitrile (43) and 1-methyl-1*H*-pyrazolo[3,4-*b*]pyridine-3-carbonitrile (43')

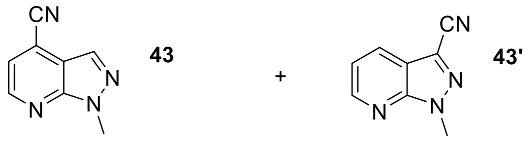

^{1}H NMR (500 MHz, $CDCl_3$)

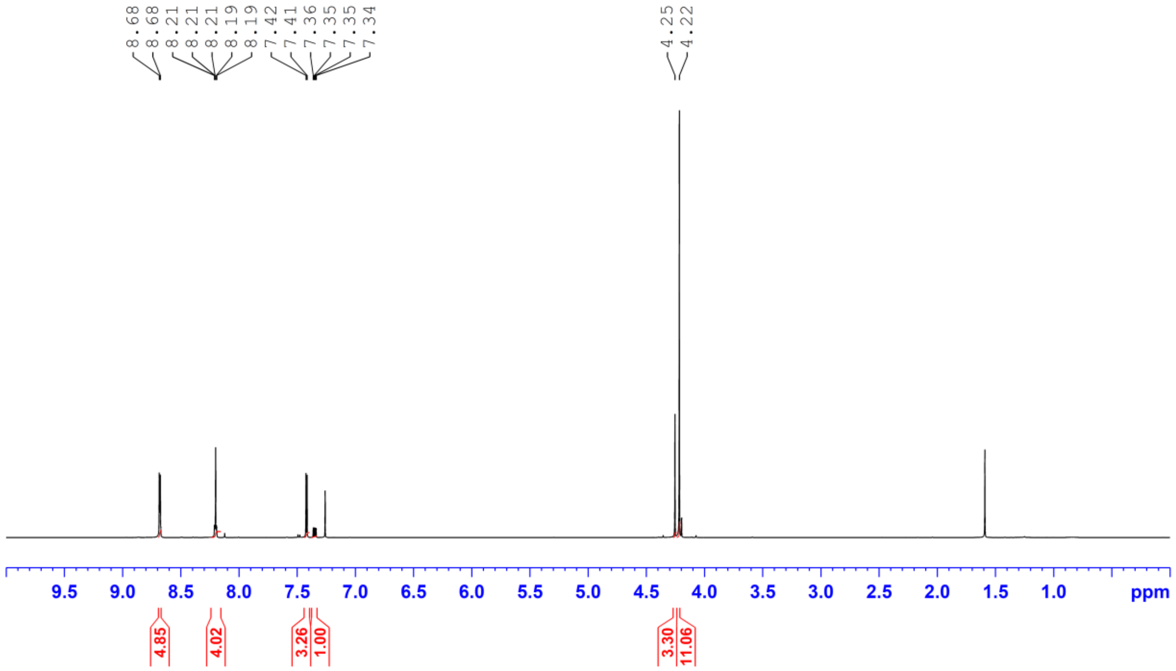

^{13}C NMR (125 MHz, $CDCl_3$)

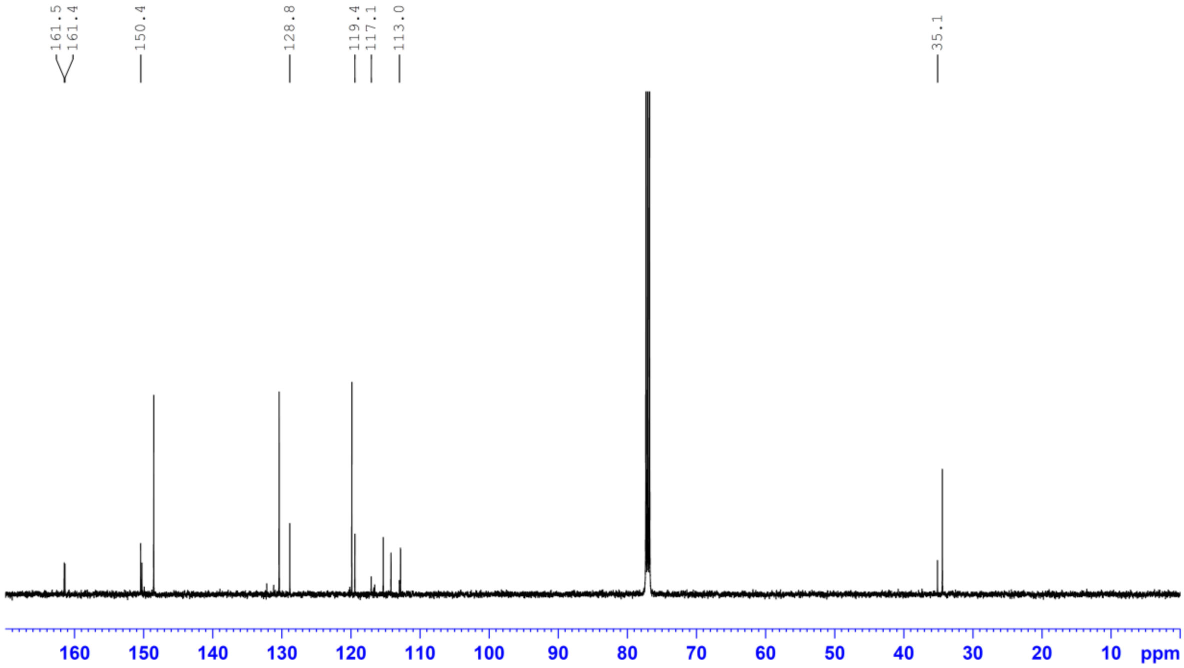

3-Bromo-1-methyl-1*H*-pyrrolo[2,3-*b*]pyridine-4-carbonitrile (44)

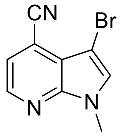

44

^{1}H NMR (400 MHz, $CDCl_3$)

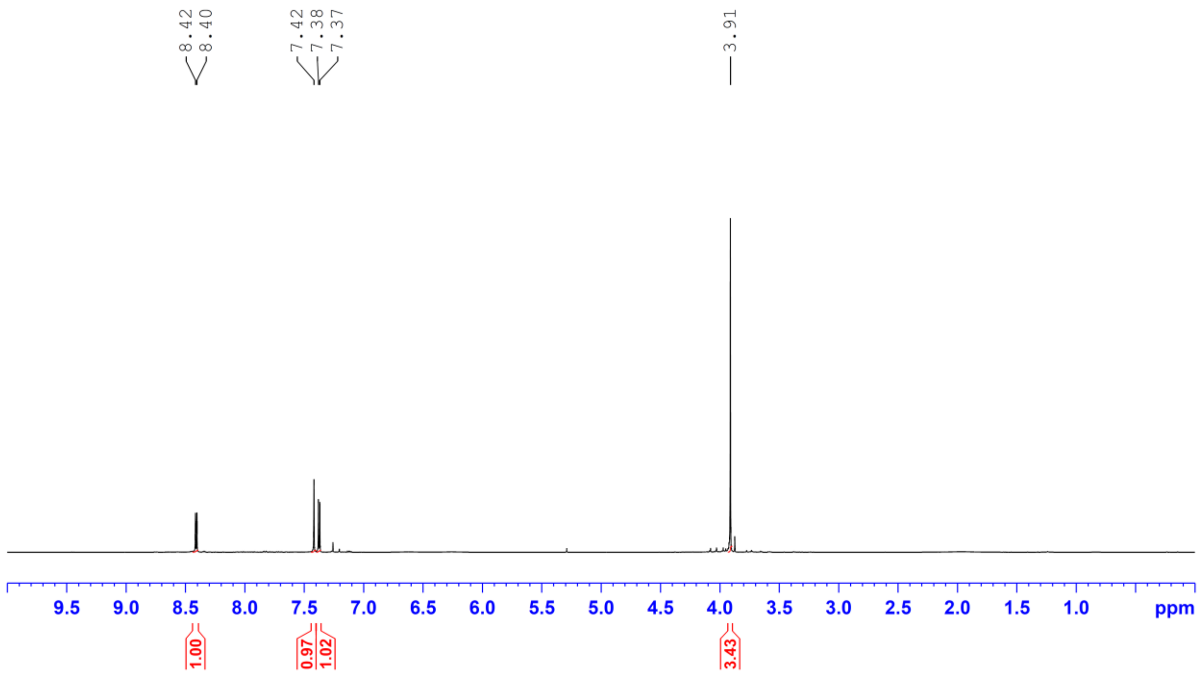

^{13}C NMR (100 MHz, $CDCl_3$)

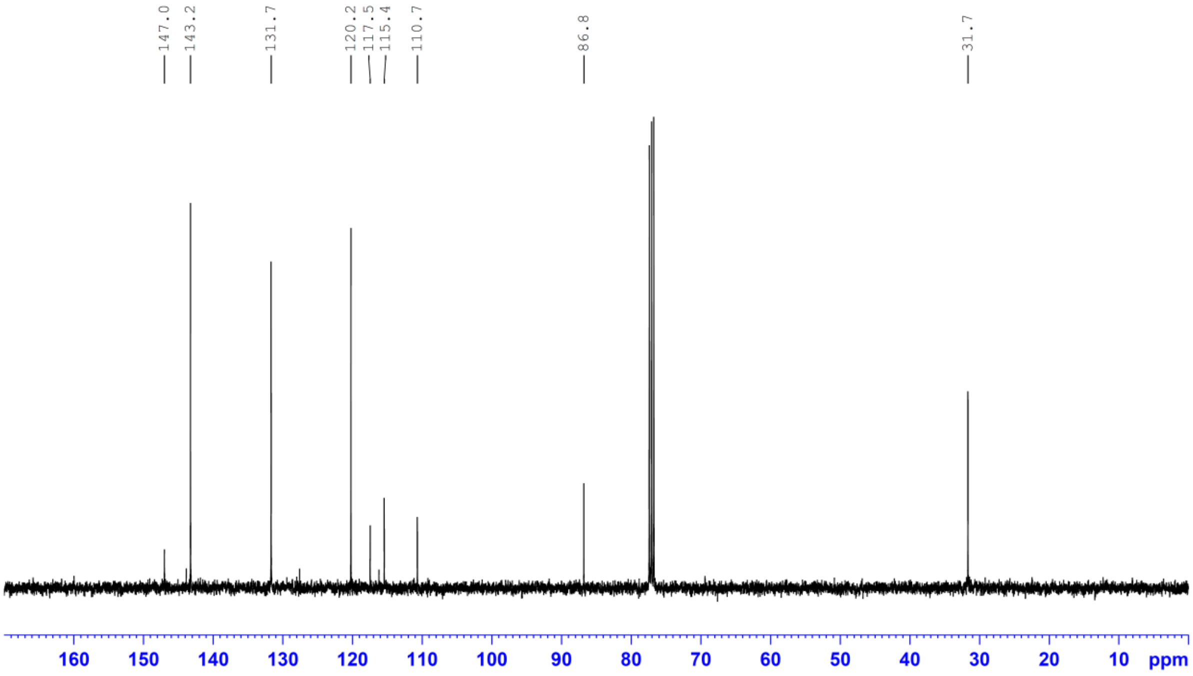

7-Methyl-7*H*-pyrrolo[2,3-*d*]pyrimidine-4-carbonitrile (45)

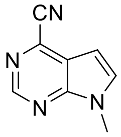

45

^{1}H NMR (400 MHz, $CDCl_3$)

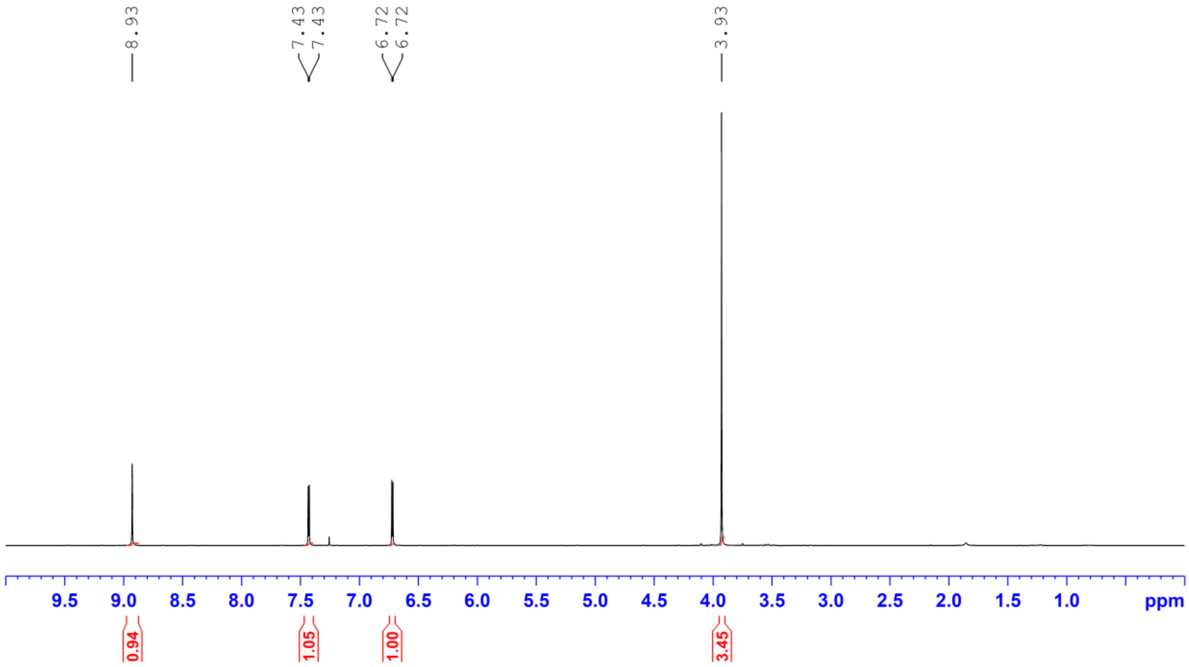

^{13}C NMR (100 MHz, $CDCl_3$)

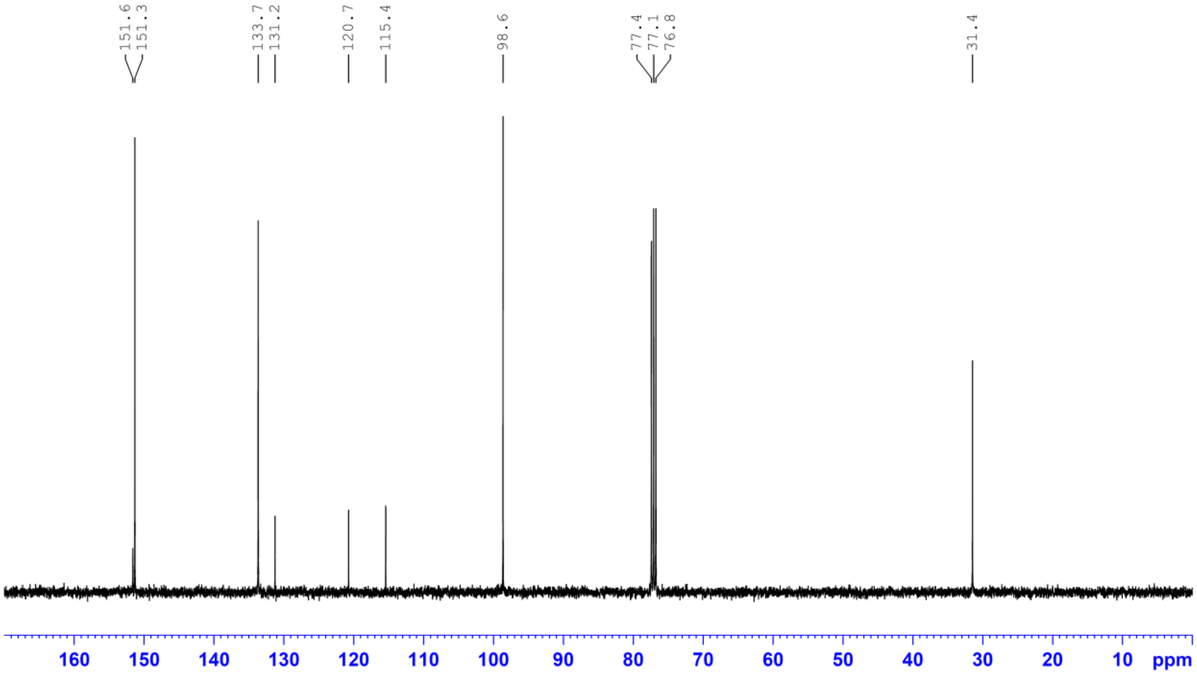

7-Bromo-1-methyl-2-phenyl-1*H*-imidazo[4,5-*c*]pyridine-4-carbonitrile (46)

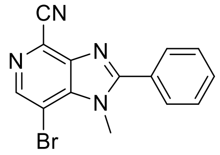

46

^{1}H NMR (400 MHz, $CDCl_3$)

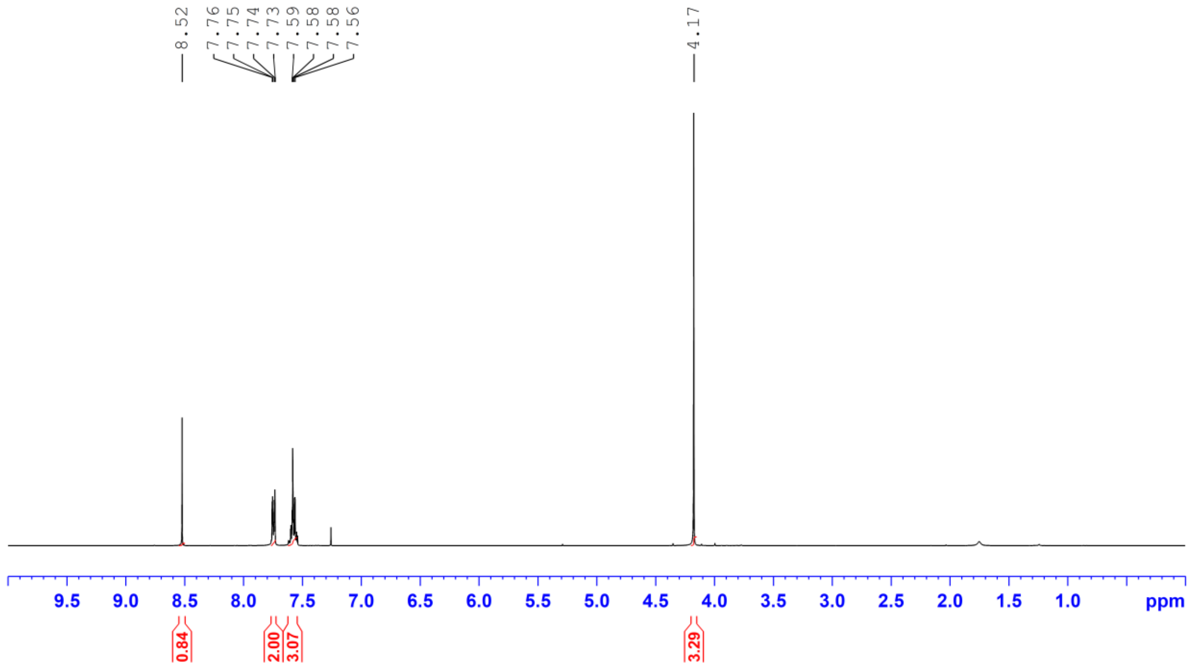

^{13}C NMR (100 MHz, $CDCl_3$)

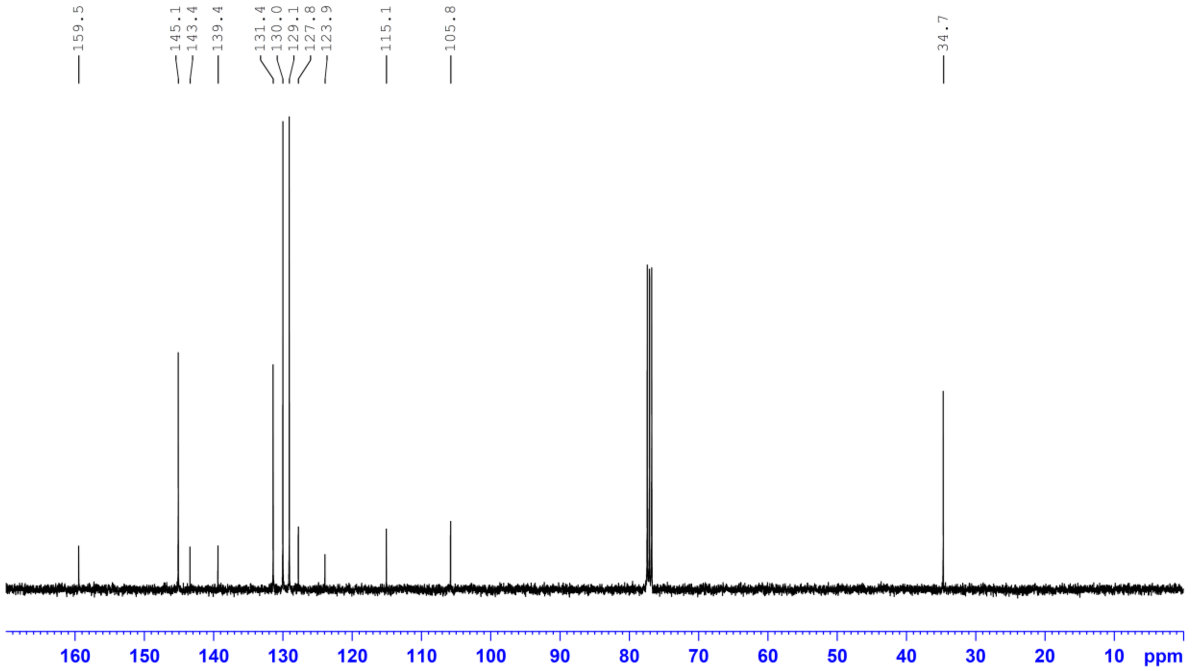

5-Bromo-2-cyclobutylpyrimidine-4-carbonitrile (47)

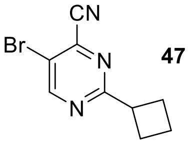

47

^{1}H NMR (400 MHz, $CDCl_3$)

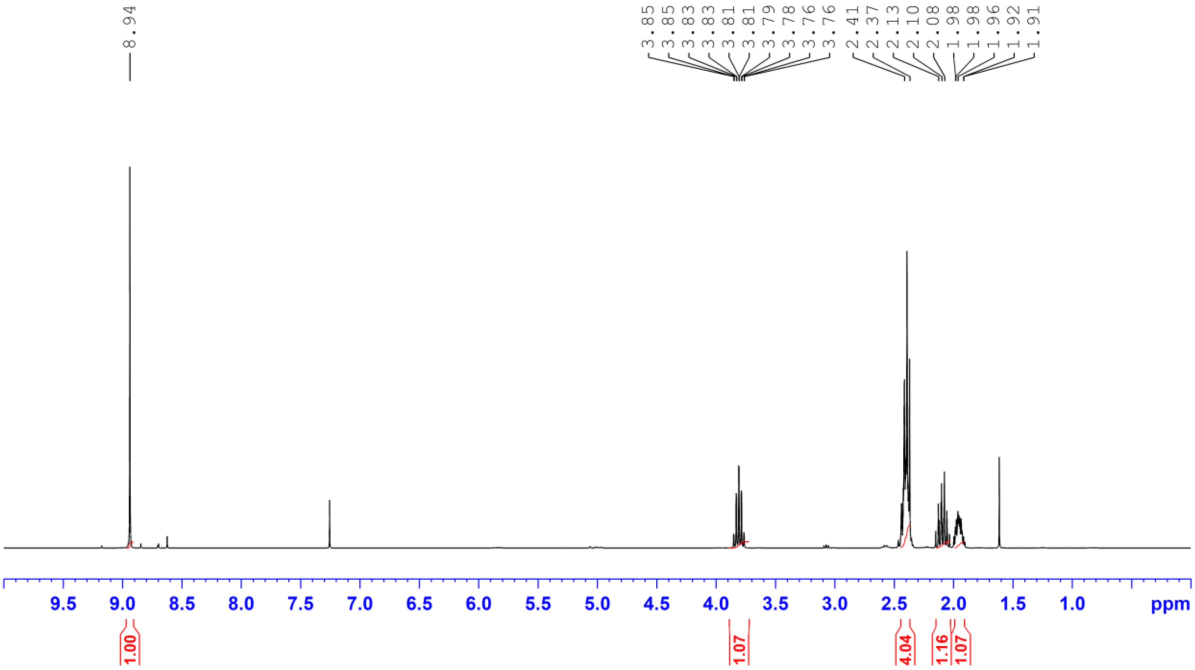

^{13}C NMR (100 MHz, $CDCl_3$)

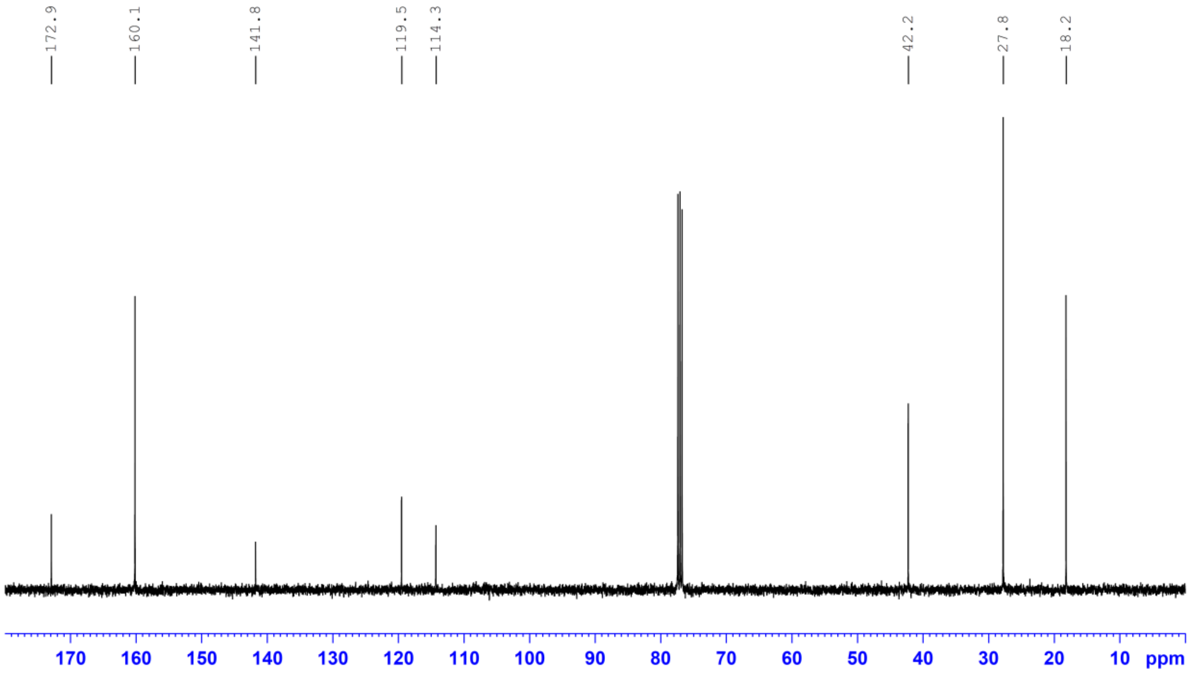

4-(1-Methyl-1*H*-pyrazol-4-yl)pyrimidine-2-carbonitrile (48)

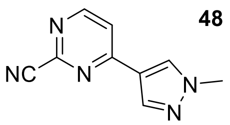

^{1}H NMR (400 MHz, $CDCl_3$)

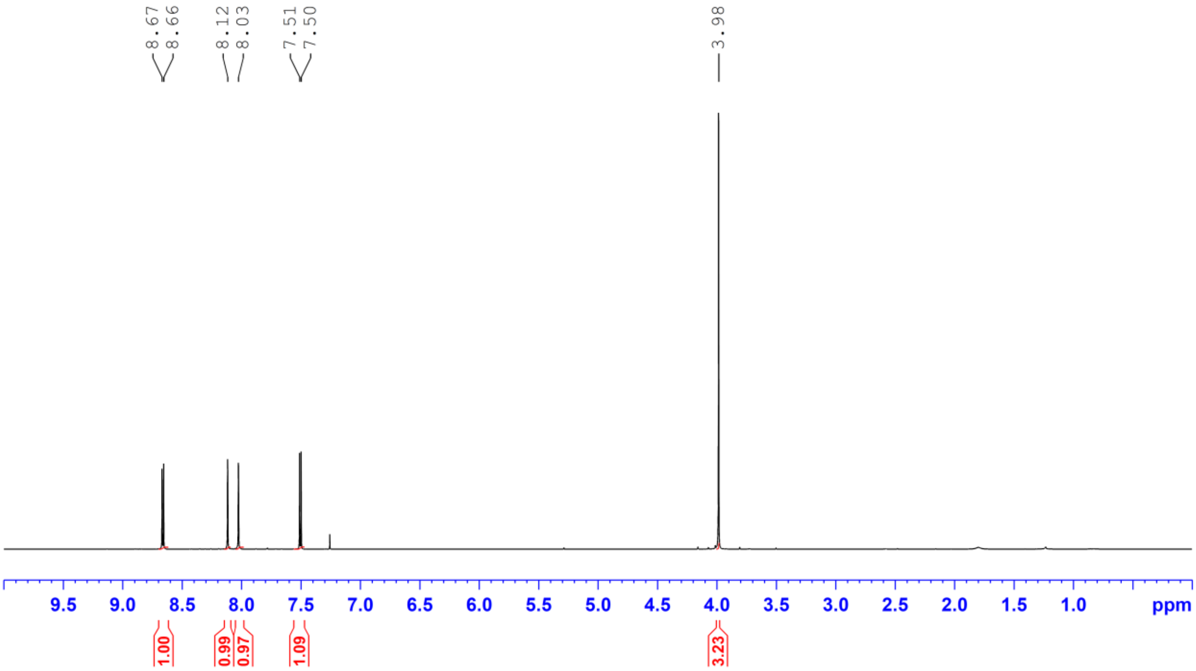

^{13}C NMR (100 MHz, $CDCl_3$)

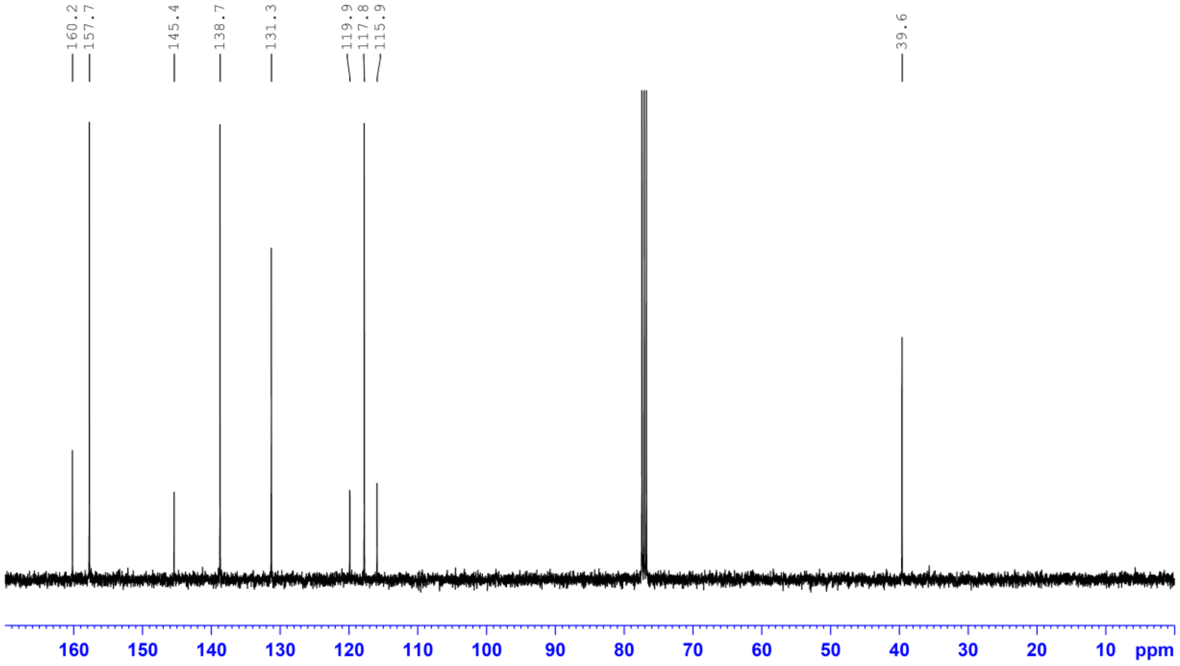

6-(1-Methyl-1*H*-pyrazol-4-yl)pyrimidine-2,4-dicarbonitrile (49)

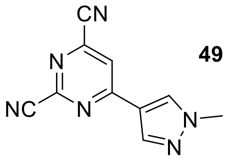

^{1}H NMR (500 MHz, $CDCl_3$)

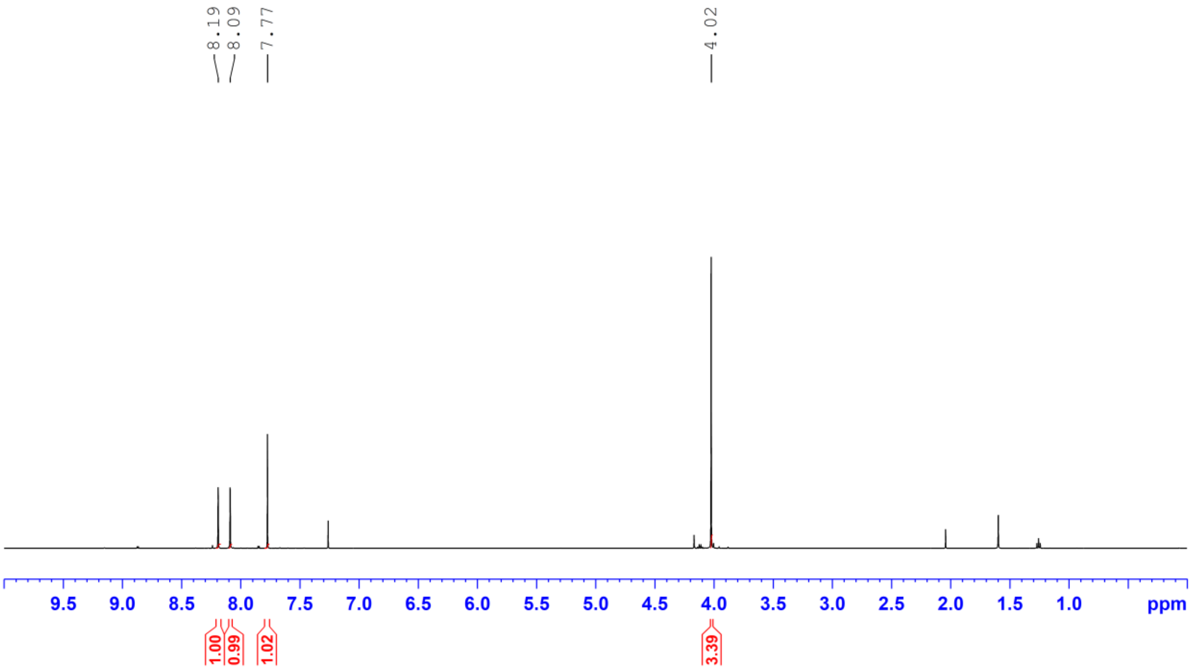

^{13}C NMR (125 MHz, $CDCl_3$)

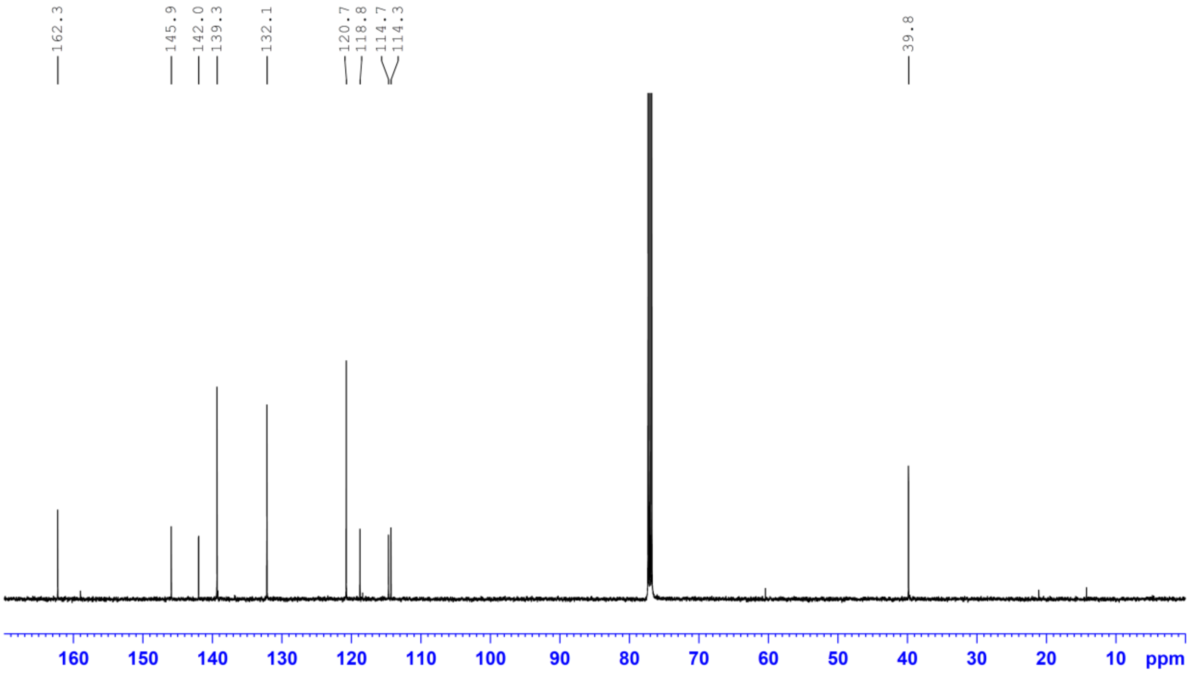

2-((2-Methoxyphenyl)(methyl)amino)pyrimidine-4-carbonitrile (50)

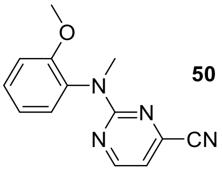

50

^{1}H NMR (400 MHz, $CDCl_3$)

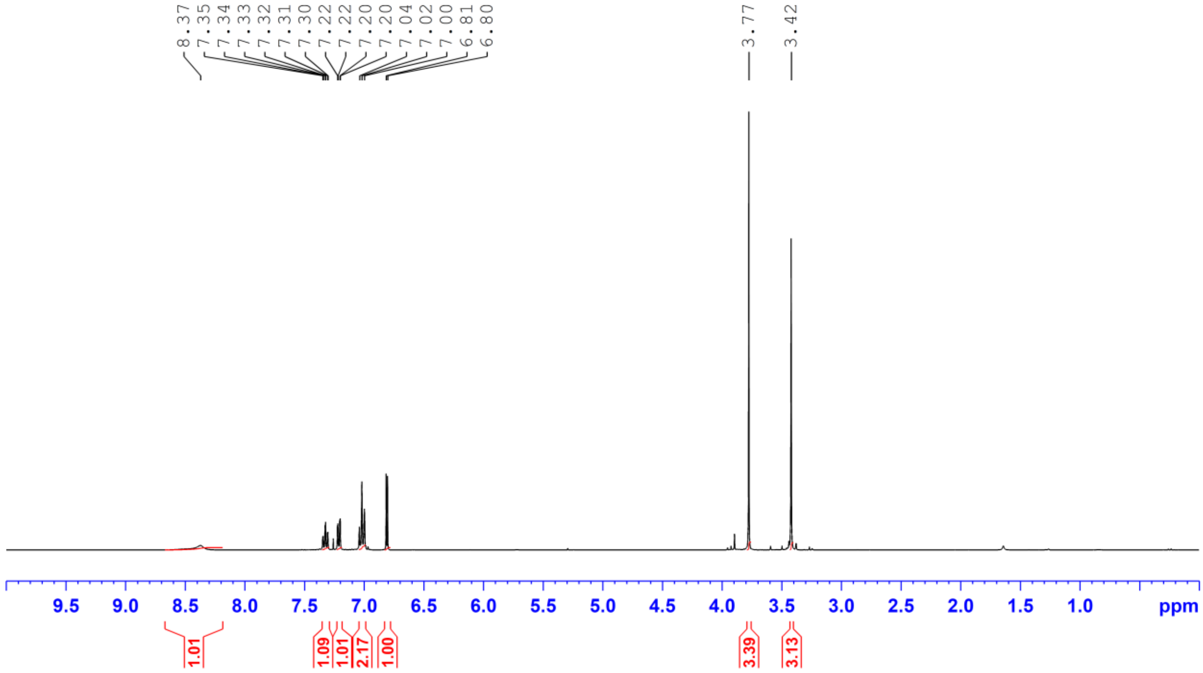

^{13}C NMR (100 MHz, $CDCl_3$)

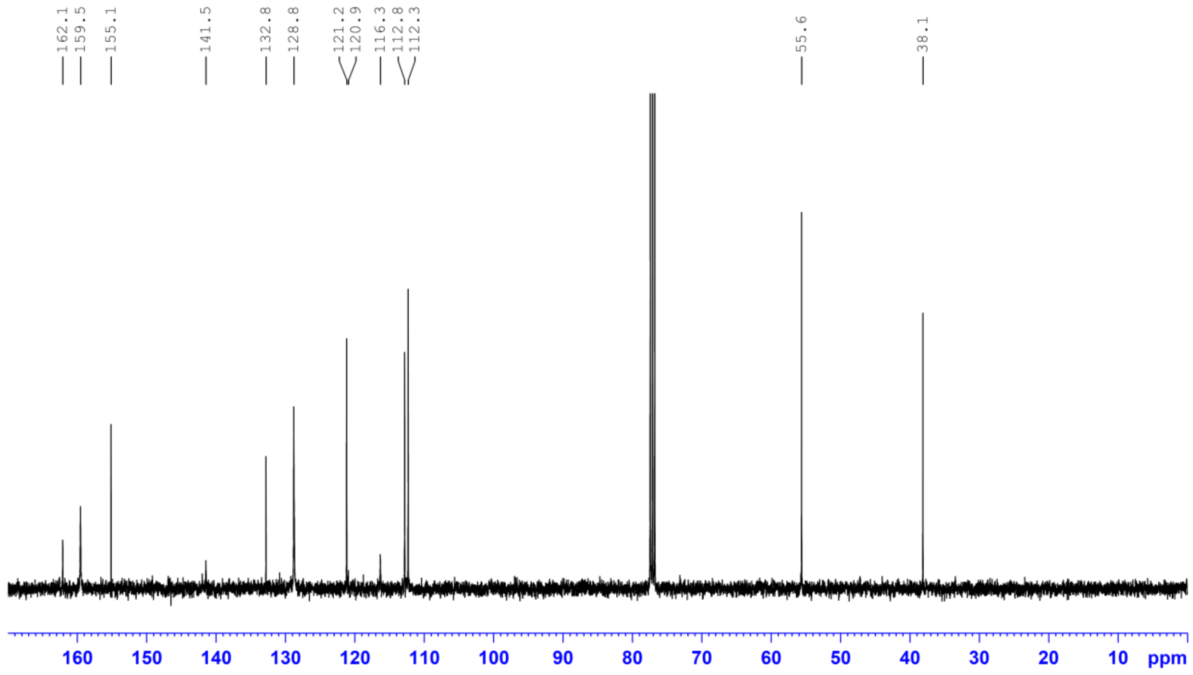

2-(Methylthio)-6-(thiophen-2-yl)pyrimidine-4-carbonitrile (51)

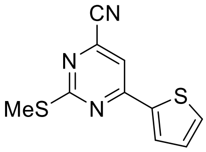

51

^{1}H NMR (500 MHz, $CDCl_3$)

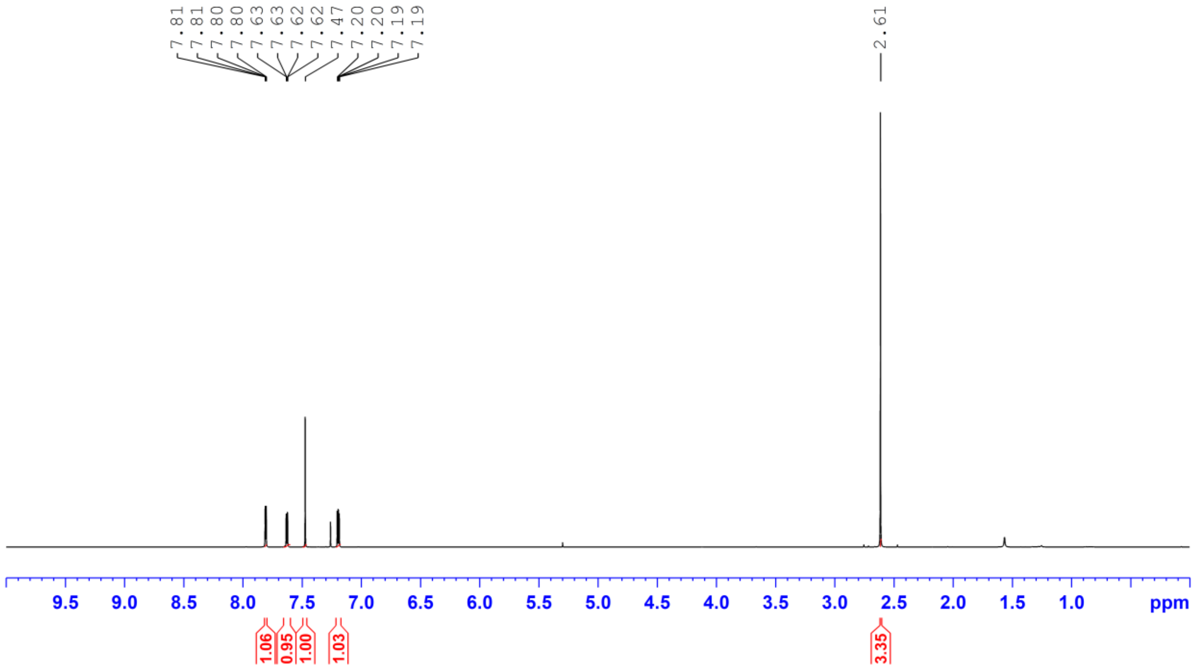

^{13}C NMR (125 MHz, $CDCl_3$)

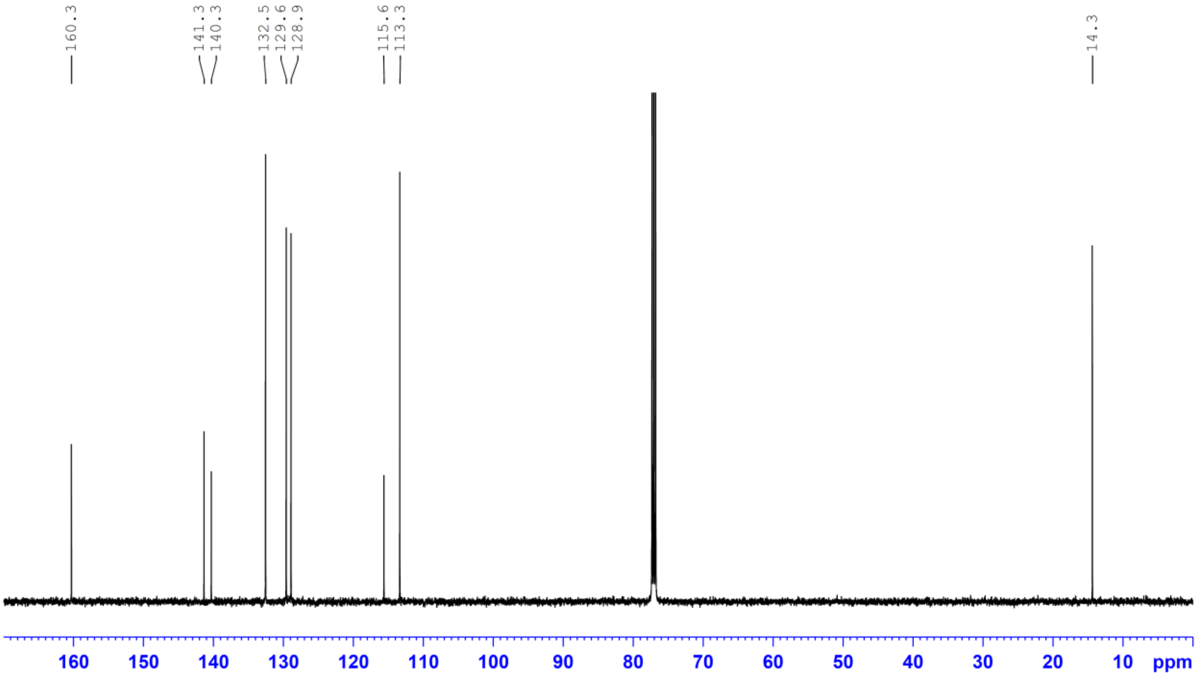

(3*S*,8*R*,9*S*,10*R*,13*S*,14*S*)-17-(4-Cyanopyridin-3-yl)-10,13-dimethyl-2,3,4,7,8,9,10,11,12,13,14,15-dodecahydro-1*H*-cyclopenta[*a*]phenanthren-3-yl acetate (52)

52

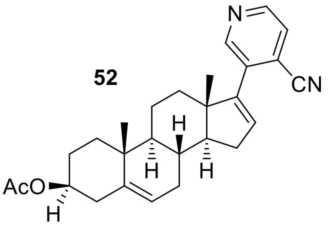

^{1}H NMR (400 MHz, $CDCl_3$)

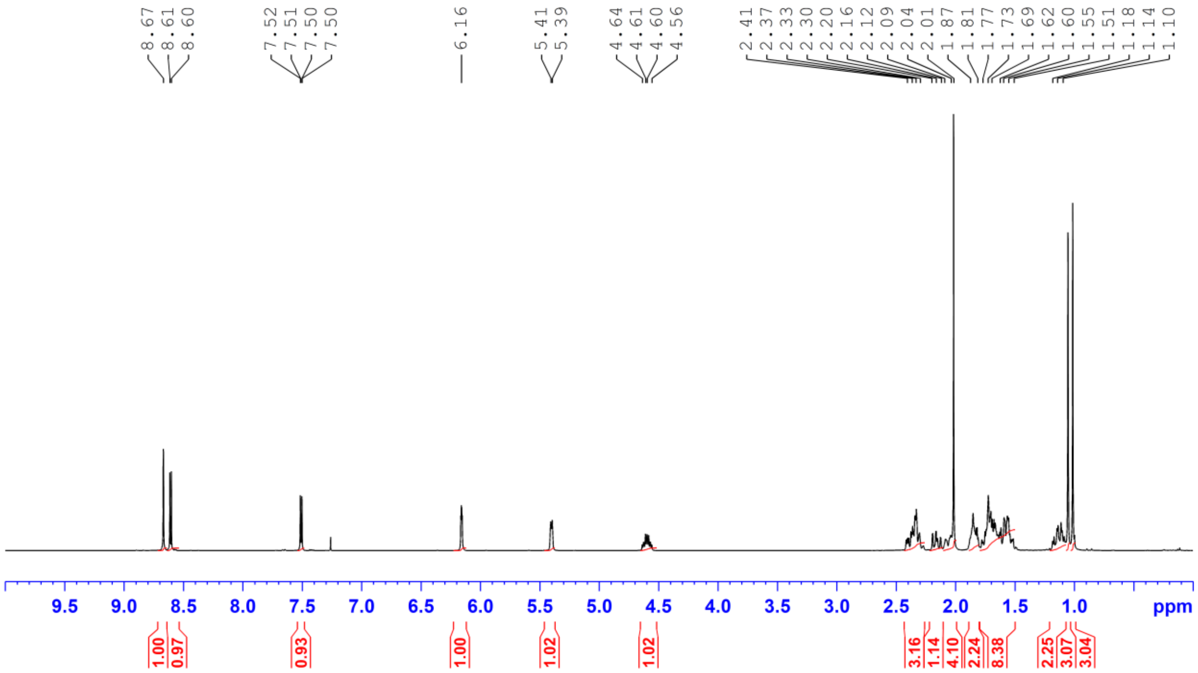

^{13}C NMR (100 MHz, $CDCl_3$)

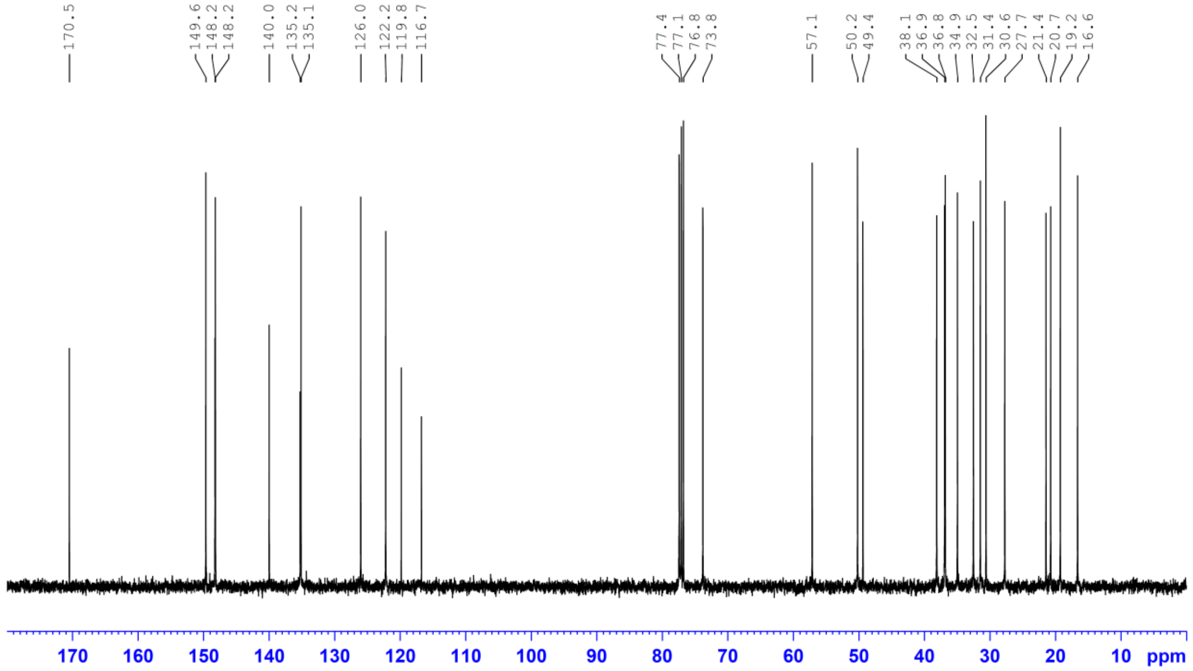

(3*S*,8*R*,9*S*,10*R*,13*S*,14*S*)-17-(6-Cyanopyridin-3-yl)-10,13-dimethyl-2,3,4,7,8,9,10,11,12,13,14,15-dodecahydro-1*H*-cyclopenta[*a*]phenanthren-3-yl acetate (52')

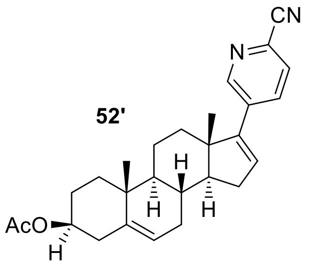

^{1}H NMR (500 MHz, $CDCl_3$)

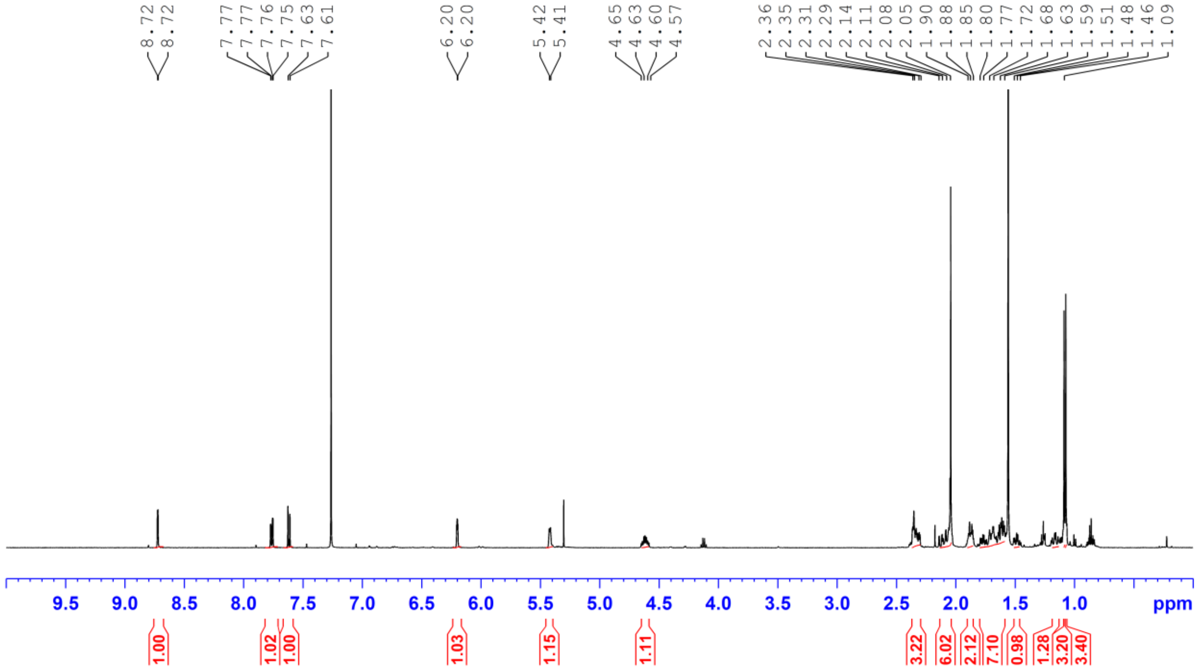

^{13}C NMR (125 MHz, $CDCl_3$)

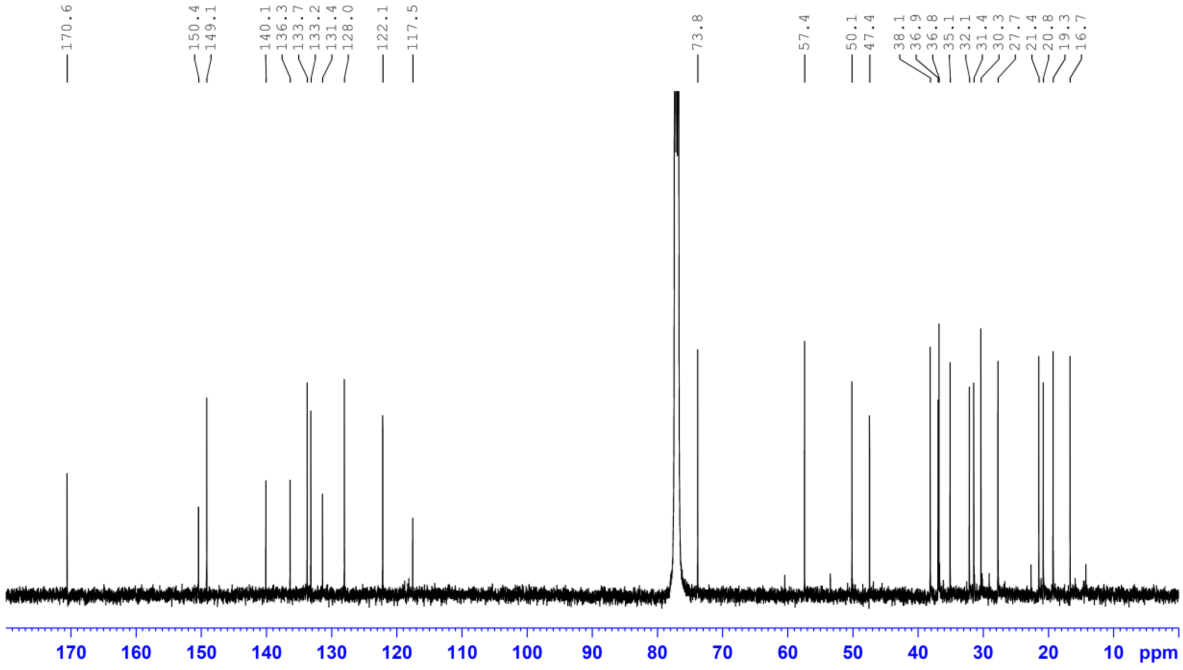

(3*S*,8*R*,9*S*,10*R*,13*S*,14*S*)-17-(2-Cyanopyridin-3-yl)-10,13-dimethyl-2,3,4,7,8,9,10,11,12,13,14,15-dodecahydro-1*H*-cyclopenta[*a*]phenanthren-3-yl acetate (52’’) and (3*S*,8*R*,9*S*,10*R*,13*S*,14*S*)-17-(4-cyanopyridin-3-yl)-10,13-dimethyl-2,3,4,7,8,9,10,11,12,13,14,15-dodecahydro-1*H*-cyclopenta[*a*]phenanthren-3-yl acetate (52)

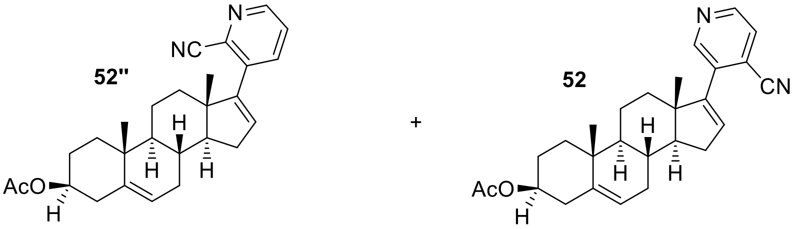

^{1}H NMR (500 MHz, CDCl$_3$)

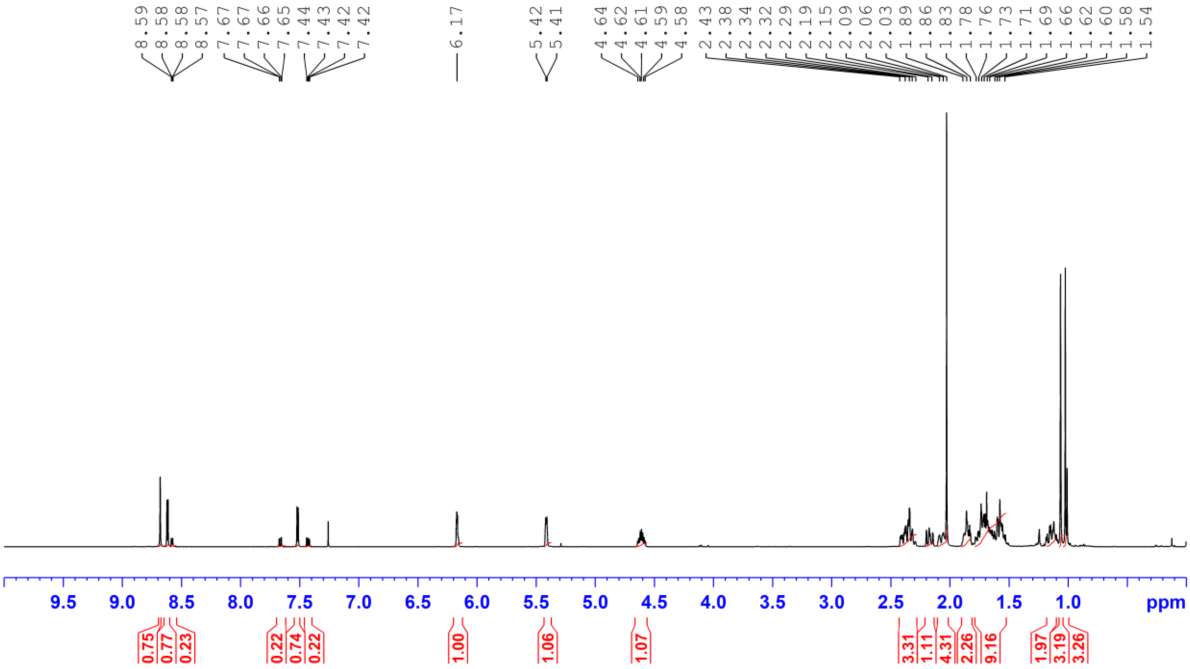

^{13}C NMR (125 MHz, CDCl$_3$)

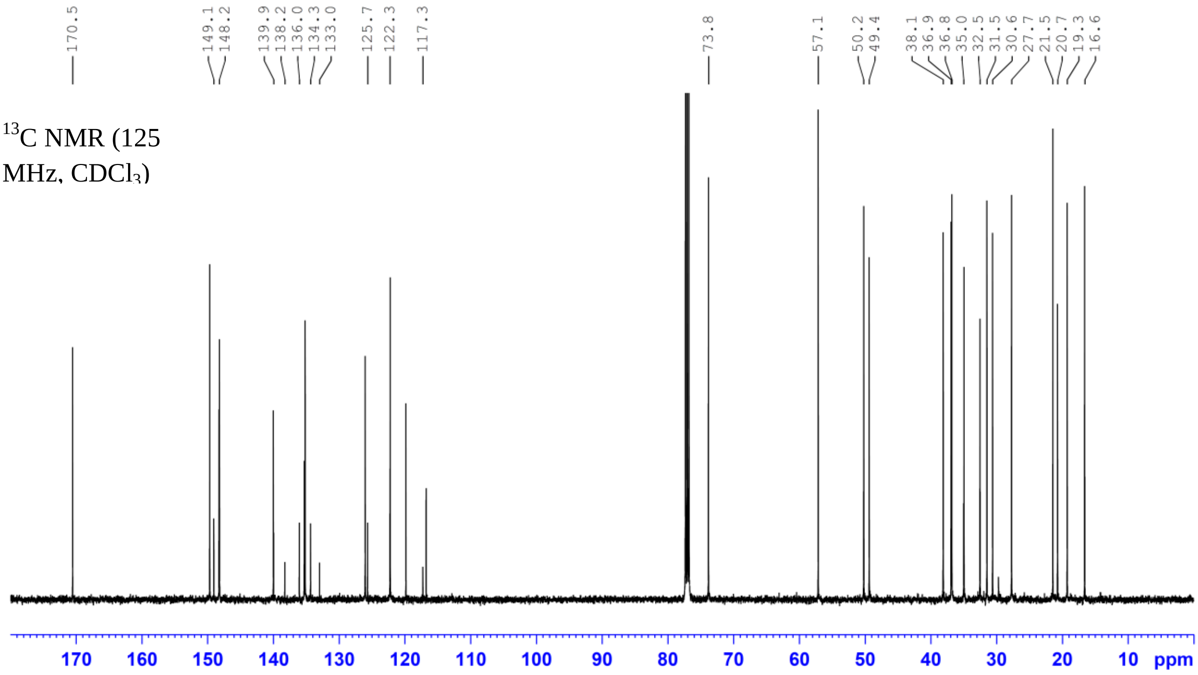

_N_-(2-Cyano-6,7-bis(2-methoxyethoxy)quinazolin-4-yl)-N-(3-ethynylphenyl)acetamide (53)

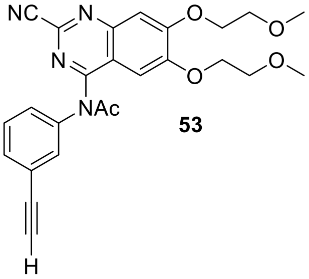

53

^{1}H NMR (400 MHz, $CDCl_3$)

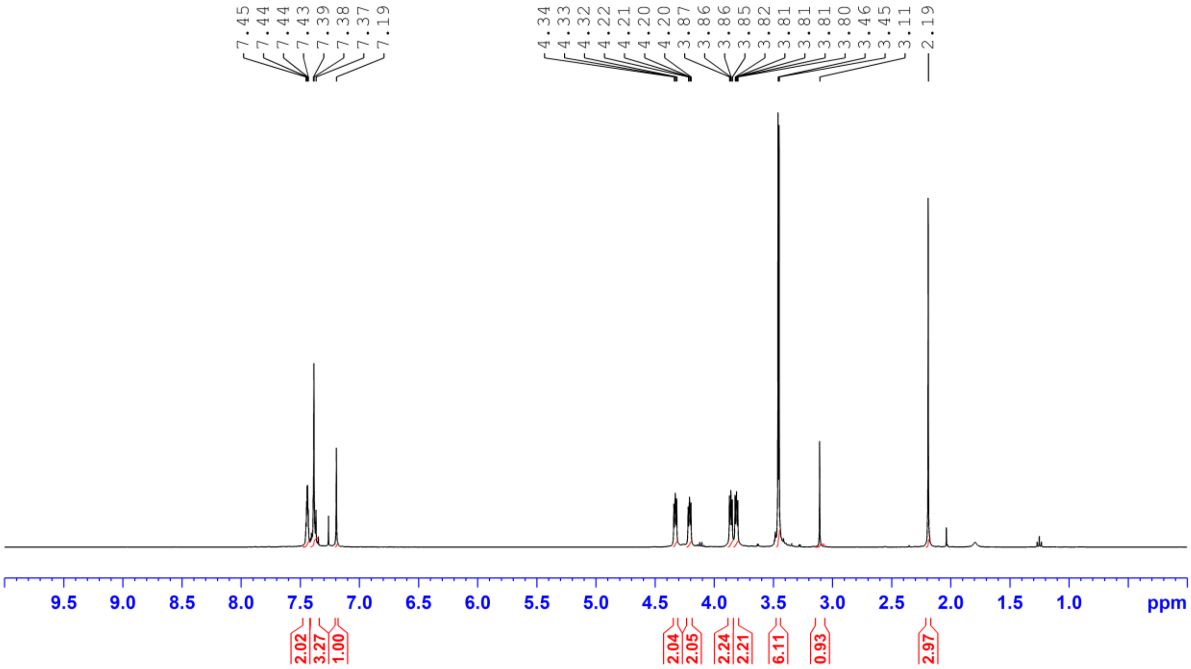

^{13}C NMR (100 MHz, $CDCl_3$)

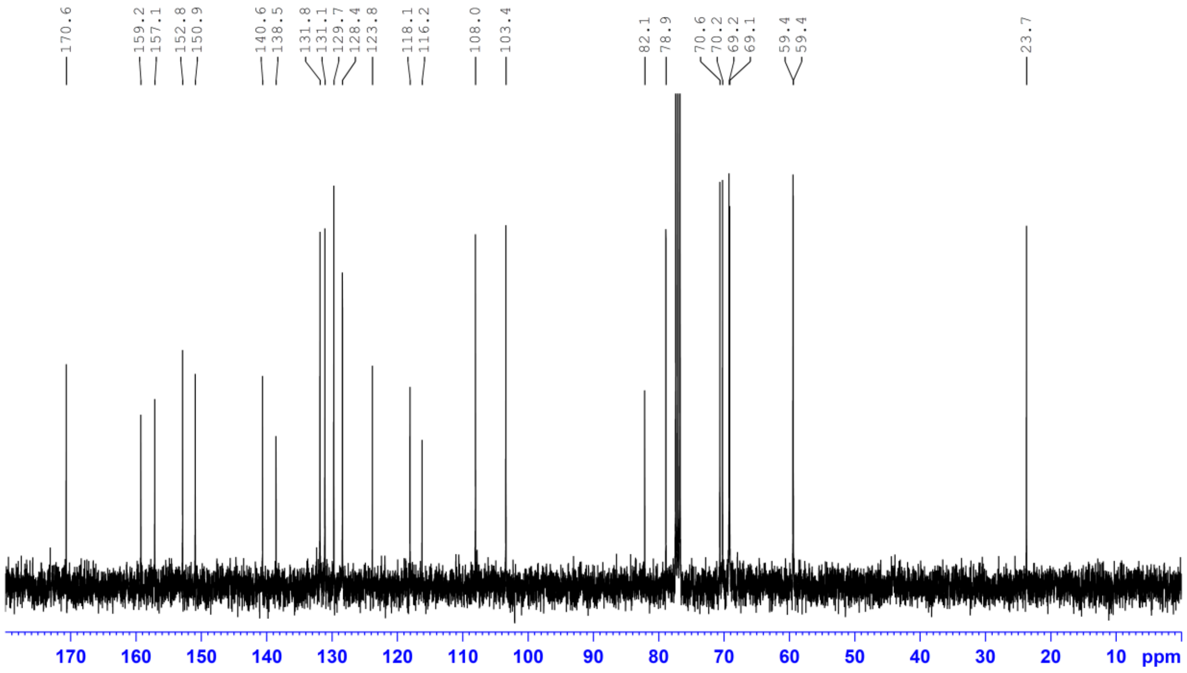

2-Methyl-1,2,3,4,10,14*b*-hexahydrobenzo[*c*]pyrazino[1,2-*a*]pyrido[3,2-*f*]azepine-9-carbonitrile (54)

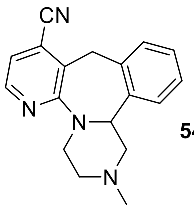

54

^{1}H NMR (500 MHz, $CDCl_3$)

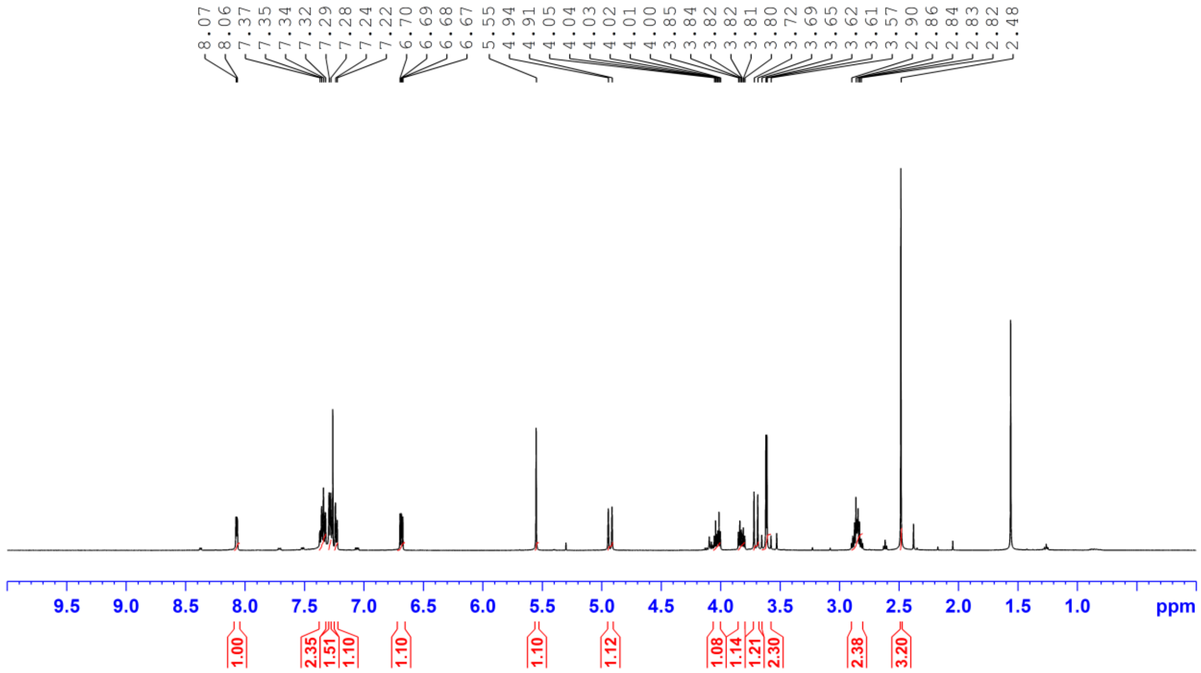

^{13}C NMR (125 MHz, $CDCl_3$)

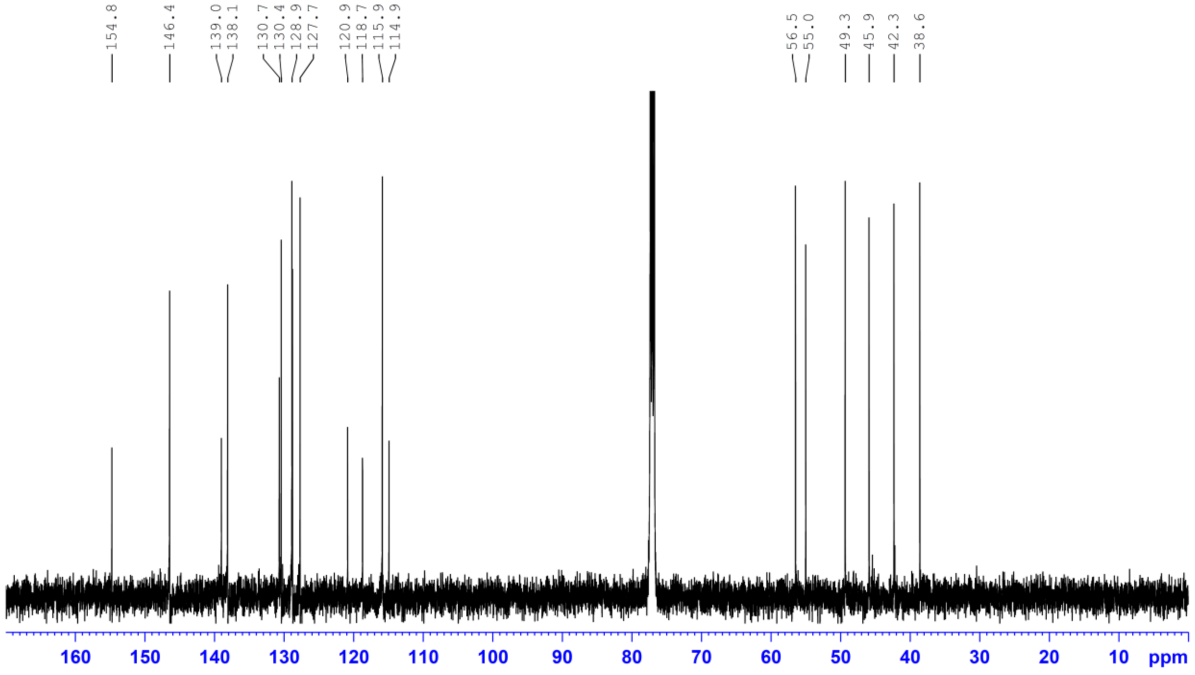

6-Methoxy-4-((*R*)-methoxy((1*S*,2*S*,4*S*,5*R*)-5-vinylquinuclidin-2-yl)methyl)quinoline-2-carbonitrile (55)

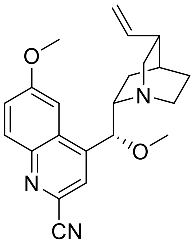

55

^{1}H NMR (400 MHz, $CDCl_3$)

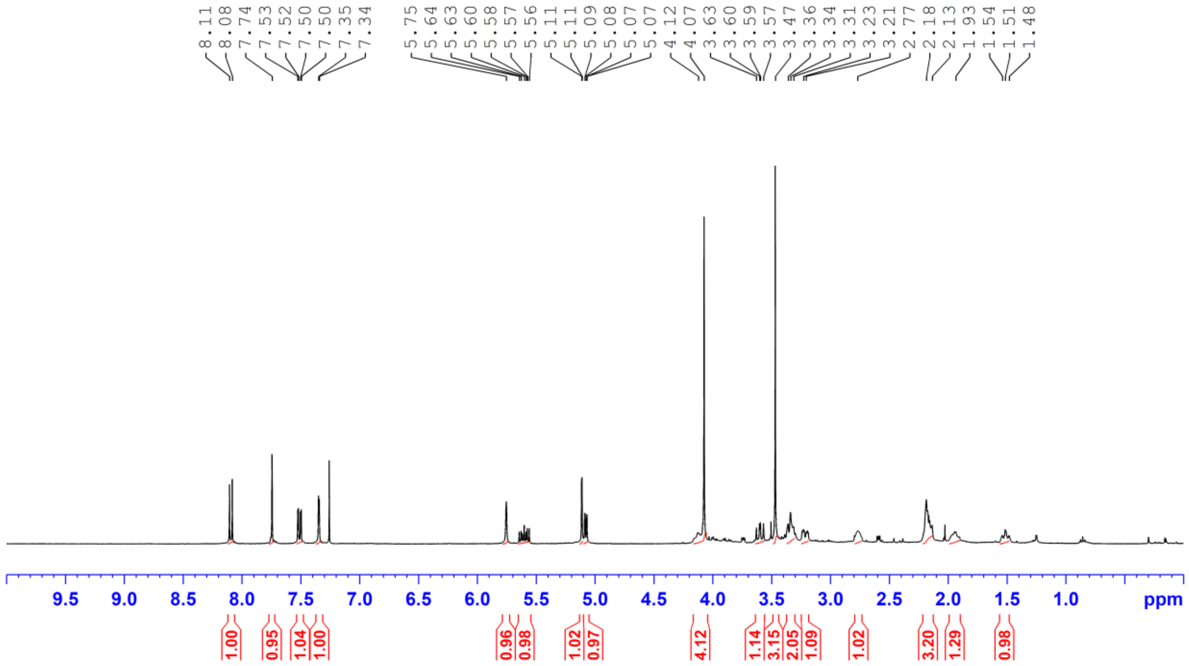

^{13}C NMR (100 MHz, $CDCl_3$)

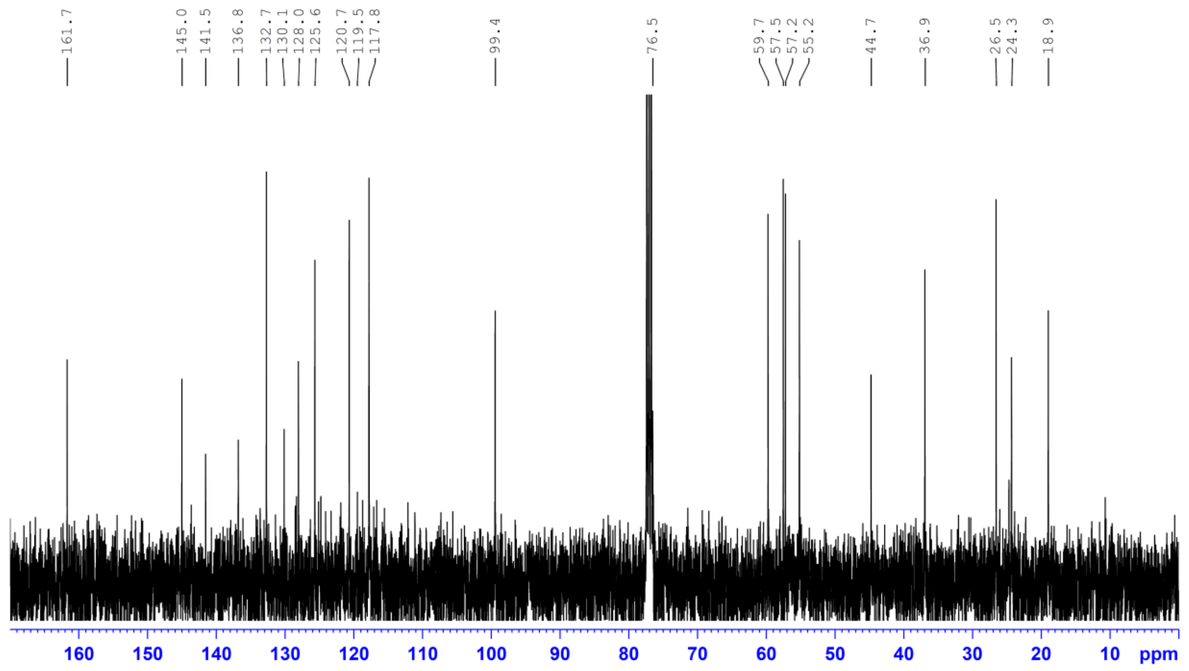

Computational results

The site of cyanide addition to most of the activated heterocycle corresponds to the most electrophilic position, indicated by the DFT condensed Fukui function, $f_{+}(r) = \rho_{N+1}(r) - \rho_{N}(r)$. The Fukui indices indicate reactivity; larger values here show which atoms more easily accept electron density and so are more susceptible to nucleophilic attack.

Figure S2 shows the condensed Fukui function, $f_{+}(r)$ for 25 heterocycles. The computed most electrophilic positions are shown in green; experimental sites of attack are shown by arrows. Where a single regioisomer is obtained, in every case the site of attack matches the most electrophilic position (largest Fukui index). Where two or more regioisomers are obtained, the selectivity is typically reflected by the different sizes of the reactivity indices, particularly well for pyridines and quinolines/isoquinolines.

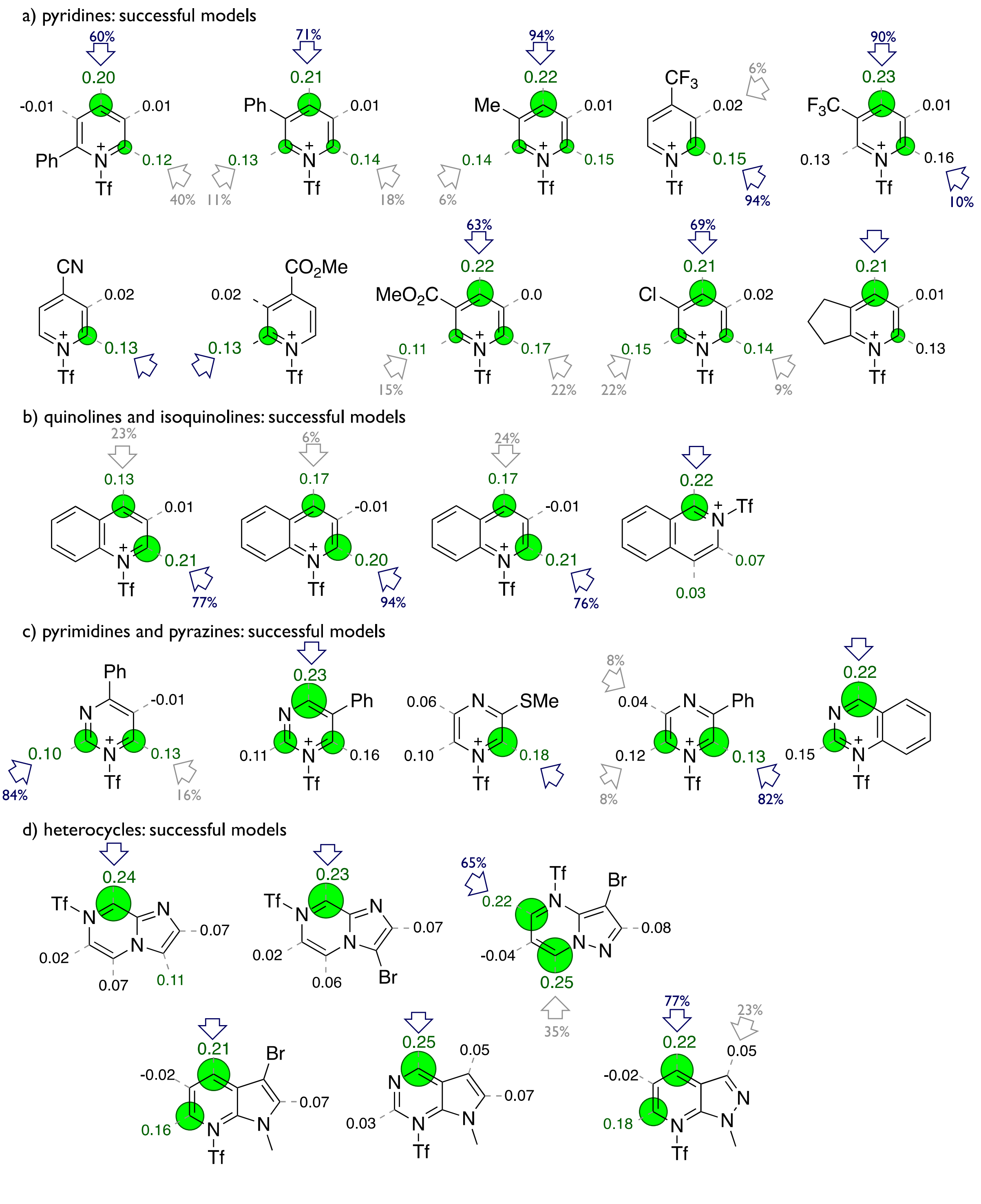

Figure S2. M06-2X/def2-SVP Fukui indices for 25 heterocycles studied experimentally.

In four cases, the computed most electrophilic position did not match the experimental selectivity (**Figure S3**). In each case, the observed product arises from attack of the second most reactive position. Steric effects are neglected by the Fukui indices, which is a possible reason for the over-estimation of reactivity ortho to the CF_3-group of trifluoromethylpyrazine. For pyridazines, reactivity at 4-position is consistently overestimated.

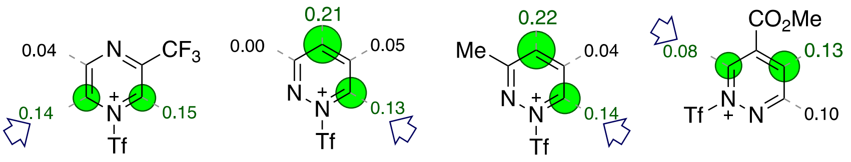

Figure S3. M06-2X/def2-SVP Fukui indices for 4 heterocycles studied experimentally.

We also computed ^{13}C NMR chemical shifts. For activated pyridines we observed that the most deshielded ^{13}C nucleus matches the position of nucleophilic addition observed in the major (or single) regioisomer formed (**Figure S4**). This is consistent with the results obtained using Fukui indices. However, the chemical shifts of other heterocycles do not match well with the observed sites of addition, presumably due to magnetic effects unrelated to chemical reactivity. We therefore focus on the use of Fukui indices which can be applied to predict regioselectivity across several heterocycle class, shown above in **Figure S2**.

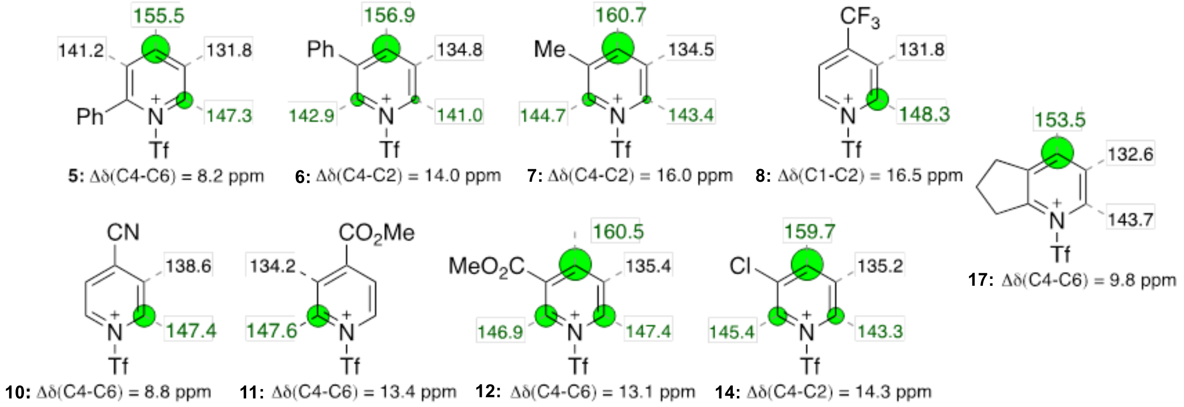

Figure S4. B3LYP/6-311+G(d,p)//M06-2X/def2-SVP GIAO ^{13}C chemical shifts (ppm) for pyridiniums.

Computational methods: Structures of heterocyclic trifluoromethanesulfonyl-pyridinium species were optimized as the free cation at the M06-2X/def2-SVP[44] level of theory with Gaussian 09.[45] Discretized Fukui electrophilic functions were computed using M06-2X/def2-SVP atomic natural populations with NBO,[46] defined as the difference between the neutral and cationic pyridinium species: $f_{+}(r) = \rho_{N+1}(r) - \rho_{N}(r)$. B3LYP/6-311+G(d,p)[47] isotropic chemical shielding tensors were calculated and ^{13}C chemical shift values obtained by reference to tetramethylsilane, computed at the same level of theory.

Cartesian Coordinates

05 Energy: -855943.2256184

C	0.36974	1.32005	-0.00192
C	0.38995	2.62301	0.50475
C	-0.74081	3.42826	0.46834
C	-1.92265	2.93113	-0.08918
C	-1.93940	1.63335	-0.54609
N	-0.82455	0.85471	-0.48408
H	-0.69871	4.44815	0.85498
H	1.33940	2.99718	0.88831
H	-2.82771	3.53361	-0.15872
H	-2.83074	1.16608	-0.96673
S	-1.14466	-0.91264	-0.87844
O	-2.41697	-0.88802	-1.53519
O	0.04029	-1.51365	-1.39455
C	-1.42498	-1.46540	0.89460
F	-2.41971	-0.75683	1.37322
F	-1.71790	-2.73210	0.85816
F	-0.33407	-1.24549	1.58482
C	1.62466	0.53535	-0.02741
C	2.29547	0.29999	1.18067
C	2.21271	0.16935	-1.24544
C	3.53108	-0.33966	1.16987
H	1.84092	0.59912	2.12774
C	3.45569	-0.45367	-1.24689
H	1.70622	0.37802	-2.18888
C	4.11034	-0.71447	-0.04253
H	4.04500	-0.54057	2.11024
H	3.91700	-0.73441	-2.19398
H	5.08282	-1.20842	-0.04975

06 Energy: -855947.2406876

C	0.31423	0.03237	-0.38061
C	1.46753	0.77942	-0.15027
C	1.30701	2.17308	-0.03521
C	0.05415	2.77125	-0.15802
C	-1.05336	1.98003	-0.40311
N	-0.88293	0.64405	-0.49748
H	2.18808	2.79704	0.12846
H	-0.06755	3.85172	-0.08502
H	-2.06715	2.36118	-0.54148
S	-2.33928	-0.38176	-0.80864
O	-3.28064	0.49327	-1.43473
O	-1.82291	-1.61046	-1.32689
C	-2.83126	-0.63177	0.98371
F	-3.11666	0.54519	1.48538
F	-3.86192	-1.42363	1.00601
F	-1.80541	-1.16105	1.60643
H	0.31699	-1.05610	-0.47330
C	2.78353	0.11921	-0.03577
C	3.74119	0.62070	0.85746
C	3.08533	-1.00829	-0.81337
C	4.98040	-0.00070	0.97305
H	3.50956	1.47982	1.49052
C	4.32882	-1.62198	-0.69865
H	2.36448	-1.39065	-1.53975
C	5.27571	-1.12050	0.19509
H	5.71624	0.38658	1.67837
H	4.56388	-2.48966	-1.31572
H	6.24917	-1.60412	0.28534

07 Energy: -735761.1321345

C	-1.38259	-0.67417	0.36474
C	-2.70198	-0.51658	-0.03747
C	-3.09028	0.76869	-0.45438
C	-2.19144	1.83403	-0.45510

C	-0.88935	1.61685	-0.04078
N	-0.53087	0.37553	0.34425
H	-4.12096	0.93358	-0.77851
H	-2.49330	2.83349	-0.76779
H	-0.12206	2.39241	0.01247
S	1.17554	0.10540	0.89082
O	1.64831	1.40010	1.26930
O	1.12022	-1.07006	1.70256
C	1.87915	-0.34117	-0.78988
F	1.73099	0.70225	-1.56869
F	3.13142	-0.64772	-0.63159
F	1.18960	-1.35865	-1.24885
H	-0.97504	-1.62265	0.72351
C	-3.66329	-1.66755	-0.01357
H	-3.16949	-2.59923	0.28570
H	-4.47917	-1.46448	0.69456
H	-4.11017	-1.81196	-1.00690

08 Energy: -922382.6916699

C	0.19845	0.04691	1.23384
C	1.56047	0.26909	1.17218
C	2.24233	-0.07274	0.00065
C	1.56303	-0.62593	-1.08357
C	0.19768	-0.82948	-0.97473
N	-0.43431	-0.48277	0.16404
H	2.08834	-0.90143	-1.99882
H	-0.41547	-1.27489	-1.76241
S	-2.22949	-0.80331	0.29315
O	-2.49141	-1.78493	-0.72093
O	-2.46819	-0.91214	1.69573
C	-2.79468	0.88407	-0.31389
F	-2.21641	1.09601	-1.47177
F	-4.08716	0.85173	-0.42252
F	-2.40049	1.76893	0.56747
H	-0.41456	0.26389	2.11226
H	2.08124	0.69705	2.03082
C	3.74739	0.16757	-0.06194
F	3.99317	1.45334	0.12728
F	4.34147	-0.52568	0.89439
F	4.23176	-0.20128	-1.23421

09 Energy: -922383.0262956

C	0.74160	-0.14277	-0.39442
C	1.97767	0.31476	0.01763
C	2.10953	1.63079	0.47298
C	0.99320	2.46638	0.50753
C	-0.23051	1.96789	0.09369
N	-0.31706	0.69125	-0.33330
H	1.06402	3.49933	0.84822
H	-1.15273	2.55459	0.07632
S	-1.94393	0.06303	-0.88335
O	-2.70091	1.24050	-1.16854
O	-1.62763	-1.01093	-1.77003
C	-2.46719	-0.63228	0.78293
F	-2.44030	0.35803	1.64177
F	-3.66099	-1.12313	0.65379
F	-1.59498	-1.55228	1.11180
H	0.57066	-1.15081	-0.78329
H	3.08759	1.99841	0.79534
C	3.18664	-0.60799	-0.01227
F	2.85923	-1.78024	-0.53381
F	4.15030	-0.06338	-0.73428
F	3.63289	-0.79078	1.21964

10 Energy: -768923.6460764

C	-0.87391	-0.37253	-1.18683
C	-2.22893	-0.10491	-1.21677
C	-2.91563	0.03762	-0.00009

C	-2.22886	-0.09970	1.21717
C	-0.87385	-0.36745	1.18830
N	-0.24304	-0.48495	0.00096
H	-2.74591	-0.00311	2.17217
H	-0.26531	-0.50637	2.08571
S	1.54705	-0.85796	0.00168
O	1.78797	-1.43609	1.28543
O	1.78792	-1.44114	-1.27979
C	2.13077	0.92890	-0.00185
F	1.64417	1.49701	1.07447
F	3.42749	0.92717	-0.00183
F	1.64412	1.49281	-1.08035
H	-0.26544	-0.51531	-2.08368
H	-2.74602	-0.01244	-2.17215
C	-4.32506	0.32087	-0.00061
N	-5.45503	0.55248	-0.00105

11 Energy: -853950.5258258

C	0.19052	-1.01276	-0.61789
C	1.55628	-0.99749	-0.40197
C	2.17257	0.21693	-0.08566
C	1.41889	1.39088	0.00643
C	0.05754	1.32915	-0.21862
N	-0.50932	0.13788	-0.51372
H	1.91147	2.33541	0.24482
H	-0.60815	2.19496	-0.18921
S	-2.29831	0.09063	-0.82502
O	-2.64238	1.43367	-1.17180
O	-2.49893	-1.08808	-1.60789
C	-2.81904	-0.24448	0.94738
F	-2.35493	0.73377	1.68676
F	-4.11598	-0.29519	0.98042
F	-2.28073	-1.38590	1.30253
H	-0.37520	-1.90802	-0.88664
H	2.13949	-1.91479	-0.48317
C	3.66414	0.32525	0.16060
O	4.17746	1.36877	0.43779
O	4.26285	-0.83841	0.02553
C	5.68571	-0.84701	0.22573
H	6.01014	-1.87127	0.02803
H	6.15933	-0.14173	-0.46801
H	5.91418	-0.55453	1.25811

12 Energy: -853951.1927868

C	-0.77621	0.00134	0.31472
C	-1.93548	0.67024	-0.03851
C	-1.88057	2.04130	-0.30669
C	-0.66520	2.72257	-0.21285
C	0.46969	2.01962	0.14655
N	0.38055	0.69157	0.38820
H	-0.59292	3.79271	-0.40598
H	1.45651	2.47336	0.26631
S	1.88429	-0.19858	0.88465
O	2.74106	0.81133	1.42165
O	1.40816	-1.37551	1.54063
C	2.46264	-0.64317	-0.84443
F	2.64043	0.47772	-1.50101
F	3.56934	-1.31306	-0.73255
F	1.51782	-1.35969	-1.40318
H	-0.75138	-1.06517	0.55403
H	-2.80807	2.55187	-0.58068
C	-3.26386	-0.04172	-0.12895
O	-4.26675	0.53637	-0.42429
O	-3.14400	-1.32366	0.15404
C	-4.35287	-2.09930	0.10440
H	-4.78354	-2.04715	-0.90295
H	-4.06440	-3.12269	0.35547
H	-5.07369	-1.70491	0.83096

14 Energy: -999417.8748781

C	-1.19464	-0.43071	0.35790
C	-2.47935	-0.08386	-0.04049
C	-2.73418	1.23568	-0.43836
C	-1.70532	2.17536	-0.42489
C	-0.43777	1.79157	-0.01994
N	-0.22977	0.51026	0.34519
H	-1.88303	3.20950	-0.72050
H	0.41843	2.46747	0.03830
S	1.44277	0.02831	0.89629
O	2.04795	1.25379	1.31246
O	1.23679	-1.15159	1.67504
C	2.10109	-0.44520	-0.79874
F	1.99894	0.61220	-1.56659
F	3.33627	-0.81633	-0.65503
F	1.35021	-1.41844	-1.25206
H	-0.92224	-1.43250	0.70035
H	-3.74344	1.51355	-0.74935
Cl	-3.71125	-1.26202	-0.02797

17 Energy: -784292.0850812

C	1.28876	-0.21081	-0.17407
C	2.51319	0.32379	0.24628
C	2.65053	1.69628	0.41429
C	1.55438	2.53051	0.15713
C	0.35768	1.97458	-0.23578
N	0.24370	0.62445	-0.38180
H	1.62417	3.61285	0.26205
H	-0.53393	2.56261	-0.45796
S	-1.39386	0.02549	-0.87633
O	-2.07368	1.18468	-1.36666
O	-1.19729	-1.19776	-1.58722
C	-2.03524	-0.34164	0.84588
F	-1.93922	0.76116	1.54821
F	-3.27097	-0.72775	0.73105
F	-1.28569	-1.28369	1.37126
H	3.60194	2.12369	0.73849
C	1.35424	-1.71236	-0.28729
H	0.84850	-2.15097	0.58971
H	0.85165	-2.10306	-1.17809
C	3.50608	-0.78565	0.46867
C	2.87351	-1.97749	-0.27252
H	4.51021	-0.52934	0.10691
H	3.58891	-0.97505	1.55168
H	3.11451	-2.94128	0.18920
H	3.24177	-2.00454	-1.30740

20 Energy: -807425.5324927

C	1.20169	-0.08112	-0.22284
C	2.43443	0.52858	0.17012
C	2.47915	1.91817	0.41264
C	1.34649	2.69715	0.27929
C	0.15955	2.08040	-0.09794
N	0.09525	0.76140	-0.33506
H	1.35349	3.77109	0.45847
H	-0.76974	2.63925	-0.22662
S	-1.54644	0.16647	-0.82567
O	-2.32050	1.35738	-1.00861
O	-1.36411	-0.85684	-1.80618
C	-2.06934	-0.57119	0.82304
F	-1.75670	0.29792	1.75434
F	-3.35548	-0.75589	0.75989
F	-1.43436	-1.69991	1.01521
H	3.42840	2.37035	0.70958
C	3.59704	-0.27435	0.31023
C	1.16037	-1.47135	-0.46207
C	2.31448	-2.21247	-0.31100

C	3.53929	-1.62440	0.07441
H	0.24956	-1.97152	-0.77476
H	4.52923	0.20771	0.60874
H	2.27229	-3.28591	-0.50093
H	4.42988	-2.24409	0.17984

21 Energy: -832068.4723368

C	0.90019	0.22040	-0.22738
C	1.99501	1.06356	0.13837
C	1.76374	2.43369	0.38322
C	0.49460	2.96971	0.27569
C	-0.55023	2.13173	-0.09164
N	-0.35285	0.82413	-0.32942
H	0.29061	4.02335	0.45959
H	-1.57143	2.49680	-0.21595
S	-1.84325	-0.07180	-0.83606
O	-2.80742	0.95009	-1.11266
O	-1.45073	-1.09915	-1.74883
C	-2.27621	-0.80543	0.83407
F	-2.42066	0.19358	1.67042
F	-3.38489	-1.47086	0.69034
F	-1.29778	-1.58900	1.22060
H	2.60828	3.06806	0.66175
C	3.29835	0.51330	0.25283
C	1.14209	-1.15316	-0.44958
C	2.42487	-1.63720	-0.32367
C	3.53656	-0.82274	0.02500
H	0.34702	-1.83613	-0.72885
H	4.11573	1.18152	0.53094
H	2.59290	-2.70161	-0.50305
C	4.90490	-1.42738	0.12990
H	4.90392	-2.26129	0.84615
H	5.21928	-1.83135	-0.84354
H	5.64755	-0.68945	0.45464

22 Energy: -832069.2137266

C	1.02839	0.35688	-0.17686
C	2.06609	1.27673	0.17626
C	1.77183	2.64425	0.32362
C	0.48474	3.11751	0.13375
C	-0.50541	2.21202	-0.21226
N	-0.24538	0.89916	-0.35927
H	0.23013	4.17059	0.24064
H	-1.53683	2.52067	-0.39229
S	-1.68152	-0.08729	-0.84498
O	-2.68404	0.87365	-1.19537
O	-1.22754	-1.14403	-1.69354
C	-2.12417	-0.74813	0.85297
F	-2.28630	0.28620	1.64198
F	-3.22672	-1.42789	0.72759
F	-1.14540	-1.50573	1.28632
H	2.57831	3.33060	0.59183
C	3.38462	0.78275	0.37417
C	1.32862	-1.01341	-0.30881
C	2.62249	-1.46805	-0.10899
C	3.65397	-0.55041	0.23423
H	0.56528	-1.73546	-0.57997
H	4.17084	1.49090	0.64030
H	4.66782	-0.92524	0.38530
C	2.95450	-2.92196	-0.25217
H	3.36673	-3.31121	0.69066
H	2.07721	-3.51890	-0.52582
H	3.72698	-3.05882	-1.02334

23 Energy: -807428.5410618

C	0.64724	-0.71461	-0.48136
C	2.00068	-0.50404	-0.19501
C	2.43737	0.83255	0.08349

C	1.47405	1.88046	0.04548
C	0.17005	1.61138	-0.24297
N	-0.20795	0.31046	-0.48985
H	1.77366	2.91098	0.23930
H	-0.61686	2.36325	-0.31151
S	-1.93482	-0.02168	-0.85457
O	-2.45974	1.20821	-1.35976
O	-1.93865	-1.29573	-1.50636
C	-2.50097	-0.25187	0.91672
F	-2.26305	0.86499	1.56201
F	-3.77080	-0.53077	0.89952
F	-1.80096	-1.23630	1.43059
C	2.92825	-1.58337	-0.18119
H	0.23728	-1.70182	-0.71442
C	3.80114	1.05507	0.37523
C	4.67873	-0.00737	0.38471
C	4.24519	-1.33048	0.10602
H	2.58166	-2.59513	-0.39785
H	4.96833	-2.14638	0.12127
H	5.73208	0.16814	0.60950
H	4.14660	2.06725	0.58893

25 Energy: -721143.3704835

C	-2.47371	-1.21165	0.64068
C	-3.40436	-0.51222	-0.15568
C	-2.98807	0.63451	-0.80769
C	-1.65979	1.03137	-0.62900
N	-0.88574	0.28937	0.15972
H	-4.42724	-0.88142	-0.24961
H	-2.73247	-2.12698	1.17674
H	-3.65234	1.22281	-1.44199
H	-1.19806	1.91291	-1.08328
S	0.87303	0.78683	0.48299
O	1.00986	1.97258	-0.30762
O	1.04898	0.65402	1.88900
C	1.67263	-0.66497	-0.43275
F	0.91715	-0.92099	-1.47643
F	2.85548	-0.26448	-0.79530
F	1.72778	-1.68394	0.37068
N	-1.23037	-0.79895	0.78819

26 Energy: -745790.6014318

C	2.45679	0.64983	0.13607
C	3.15235	-0.44716	-0.44218
C	2.47944	-1.62042	-0.70815
C	1.11829	-1.67357	-0.38361
N	0.57598	-0.59442	0.16639
H	4.21465	-0.34326	-0.67286
H	2.96846	-2.48603	-1.15633
H	0.46074	-2.53266	-0.54238
S	-1.20258	-0.59621	0.66454
O	-1.65611	-1.88027	0.22157
O	-1.22905	-0.07938	1.99113
C	-1.76618	0.72366	-0.56789
F	-1.07999	0.54038	-1.67285
F	-3.03659	0.52504	-0.76627
F	-1.53256	1.89641	-0.05882
N	1.17050	0.53781	0.42903
C	3.12463	1.95009	0.43559
H	3.93342	1.79720	1.16480
H	3.57125	2.36636	-0.47855
H	2.39895	2.66003	0.84560

27 Energy: -863977.0923522

C	-1.38452	-0.69533	0.57529
C	-2.15350	0.32580	-0.02394
C	-1.51407	1.46675	-0.47582
C	-0.13012	1.53767	-0.30322

N	0.47901	0.51147	0.28829
H	-2.08019	2.27235	-0.94946
H	0.50314	2.37421	-0.61199
S	2.30684	0.55856	0.58935
O	2.68522	1.81031	0.00634
O	2.47902	0.14195	1.93958
C	2.75516	-0.84324	-0.60281
F	1.92970	-0.76328	-1.62142
F	3.98236	-0.63059	-0.97601
F	2.62417	-1.97431	0.02216
N	-0.07957	-0.58335	0.72299
H	-1.83861	-1.61841	0.94096
C	-3.65412	0.21990	-0.20171
O	-4.27832	1.09161	-0.72883
O	-4.11394	-0.91035	0.28498
C	-5.53219	-1.12381	0.17096
H	-5.72531	-2.09650	0.62888
H	-6.06716	-0.32519	0.69906
H	-5.81907	-1.12116	-0.88767

28 Energy: -866000.8835071

C	1.71362	0.11785	-0.21799
C	0.99155	1.34450	-0.24713
C	-0.36395	1.30787	-0.41725
N	-0.98084	0.10082	-0.55494
H	1.48224	2.30905	-0.14326
H	-1.00226	2.19347	-0.46392
S	-2.74693	0.02698	-0.79615
O	-3.10891	1.32836	-1.26612
O	-2.98501	-1.22526	-1.44340
C	-3.21486	-0.11935	1.01051
F	-2.74515	0.94202	1.62431
F	-4.50996	-0.18361	1.09354
F	-2.64822	-1.20578	1.48151
C	3.15004	0.04200	-0.02526
C	3.92998	1.19997	0.18174
C	3.77725	-1.22190	-0.04245
C	5.29916	1.09198	0.36674
H	3.47767	2.19124	0.20539
C	5.14829	-1.32067	0.14225
H	3.17240	-2.11326	-0.20370
C	5.90882	-0.16692	0.34673
H	5.89829	1.98806	0.52856
H	5.63003	-2.29842	0.12670
H	6.98733	-0.24670	0.49211
N	1.03914	-1.04958	-0.38096
C	-0.24424	-1.04699	-0.54439
H	-0.79565	-1.98245	-0.69106

29 Energy: -865992.1534636

C	-1.26165	2.12784	-0.07685
C	-1.46749	0.73268	-0.16094
C	-0.31875	-0.02049	-0.37665
N	0.87034	0.60771	-0.49811
H	-0.31162	-1.11123	-0.45518
S	2.36153	-0.38255	-0.80793
O	1.86470	-1.62955	-1.29963
O	3.26369	0.51267	-1.45939
C	2.86298	-0.58248	0.99103
F	1.84360	-1.11356	1.62296
F	3.90579	-1.35488	1.03235
F	3.12403	0.61236	1.45980
H	-2.11369	2.79797	0.07446
C	-2.79602	0.10991	-0.03463
C	-3.11452	-1.03550	-0.77965
C	-3.74784	0.66056	0.83637
C	-4.37009	-1.62041	-0.65370
H	-2.39754	-1.45364	-1.49012

C	-4.99958	0.06691	0.96238
H	-3.50386	1.53442	1.44407
C	-5.31146	-1.07141	0.21820
H	-4.61903	-2.50220	-1.24469
H	-5.73231	0.48975	1.65023
H	-6.29483	-1.53279	0.31760
C	0.97786	1.95499	-0.41321
H	1.97430	2.38973	-0.54268
N	-0.06955	2.70843	-0.19686

31 Energy: -995581.4242599

C	-2.16525	-0.01534	-0.06188
C	-0.85637	-0.40742	-0.43191
N	0.10826	0.50874	-0.36612
H	-0.59262	-1.40840	-0.78534
S	1.80649	0.02365	-0.87828
O	1.61346	-1.14971	-1.67006
O	2.42015	1.25247	-1.26940
C	2.41605	-0.46351	0.83377
F	1.64875	-1.43509	1.26058
F	3.65213	-0.84090	0.71592
F	2.30130	0.58949	1.60380
C	-0.10501	1.78363	0.02138
H	0.74098	2.47235	0.01481
S	-3.42660	-1.19550	-0.15193
C	-4.84910	-0.21072	0.36932
H	-5.00626	0.62184	-0.32528
H	-5.69924	-0.90173	0.33319
H	-4.70107	0.16012	1.38946
C	-1.40904	2.11522	0.37817
N	-2.40171	1.23660	0.33691
H	-1.64209	3.13327	0.70095

32 Energy: -865988.5894232

C	0.23417	2.67319	0.25303
C	-0.95788	2.03200	-0.08146
N	-0.86692	0.72654	-0.38919
H	-1.93891	2.50800	-0.12976
S	-2.39614	-0.16772	-0.86006
O	-3.30196	0.86169	-1.26052
O	-1.93691	-1.27745	-1.63464
C	-2.84088	-0.75817	0.86872
F	-2.99232	0.30331	1.61975
F	-3.93966	-1.44387	0.77707
F	-1.84652	-1.49374	1.30143
C	0.28882	0.05288	-0.39532
H	0.24505	-0.99490	-0.69490
C	1.47115	0.73596	-0.04044
N	1.39320	2.03826	0.27245
H	0.22640	3.73665	0.50609
C	2.77857	0.06689	-0.01034
C	3.92050	0.82644	0.29247
C	2.91075	-1.30717	-0.27493
C	5.17018	0.21961	0.32649
H	3.81494	1.89126	0.49689
C	4.16260	-1.90743	-0.23786
H	2.04742	-1.93149	-0.50950
C	5.29356	-1.14509	0.06223
H	6.05366	0.81366	0.56153
H	4.25834	-2.97397	-0.44180
H	6.27554	-1.61931	0.09133

33 Energy: -932419.4034431

C	1.08993	2.35358	0.56111
C	-0.17752	1.93284	0.15112
N	-0.30000	0.67972	-0.30996
H	-1.07266	2.56062	0.16881
S	-1.96982	0.09681	-0.88442

O	-2.68513	1.30911	-1.12080
O	-1.66361	-0.94512	-1.80988
C	-2.49010	-0.64219	0.77113
F	-2.42473	0.32182	1.65495
F	-3.69596	-1.09608	0.63845
F	-1.63464	-1.59068	1.05314
C	0.74210	-0.16157	-0.40281
H	0.57627	-1.16071	-0.81600
C	1.97744	0.31531	0.02854
N	2.13778	1.54311	0.49983
H	1.23897	3.36718	0.94199
C	3.21709	-0.57922	-0.01365
F	2.88688	-1.75371	-0.53996
F	4.15062	-0.01813	-0.75003
F	3.66742	-0.76325	1.21005

34 Energy: -817459.3367384

C	0.70939	-0.89171	-0.25727
C	2.06527	-0.57215	-0.04563
C	2.40530	0.81143	-0.06850
C	1.34230	1.74291	-0.29695
N	-0.15827	0.08518	-0.46117
H	1.53306	2.81920	-0.32266
S	-1.90362	-0.31512	-0.81526
O	-2.34489	0.64865	-1.78026
O	-1.88222	-1.74061	-0.95916
C	-2.58954	0.09921	0.88345
F	-2.28639	1.33506	1.17658
F	-3.87798	-0.07793	0.83279
F	-2.02271	-0.72838	1.73102
C	3.06035	-1.55474	0.17034
H	0.31674	-1.91317	-0.27710
C	3.74192	1.20167	0.12731
C	4.70089	0.22787	0.33679
C	4.36274	-1.14658	0.35804
H	2.79135	-2.61214	0.18328
H	5.14570	-1.88751	0.52364
H	5.74112	0.52021	0.48903
H	4.00885	2.25943	0.11177
N	0.10542	1.38987	-0.48986

35 Energy: -817472.7336264

C	-1.21143	-0.09604	0.21737
C	-2.41304	0.57100	-0.16722
C	-2.35740	1.96316	-0.39152
C	-0.15439	2.04507	0.09531
N	-0.08617	0.72236	0.33447
H	0.77250	2.61356	0.22051
S	1.54845	0.11391	0.84401
O	2.30506	1.29673	1.11582
O	1.33197	-0.96426	1.75553
C	2.08867	-0.53734	-0.83137
F	1.98699	0.45344	-1.68300
F	3.31809	-0.94134	-0.71205
F	1.29036	-1.52048	-1.17297
H	-3.26393	2.50256	-0.68834
C	-3.61580	-0.16256	-0.32041
C	-1.22551	-1.48537	0.43568
C	-2.41883	-2.16586	0.27391
C	-3.61702	-1.51829	-0.10081
H	-0.33351	-2.02870	0.73237
H	-4.52307	0.36842	-0.61375
H	-2.42776	-3.24360	0.44489
H	-4.53347	-2.09761	-0.21436
N	-1.25277	2.67706	-0.26442

39 Energy: -803638.1856199

C	0.87200	-0.78680	-0.49572

C	2.21075	-0.63531	-0.17855
C	1.82321	1.72839	0.23541
C	0.51991	1.54059	-0.07927
N	0.06564	0.28015	-0.43254
H	2.22735	2.70129	0.51286
H	-0.21084	2.34929	-0.09076
S	-1.66091	0.07631	-0.86646
O	-2.09754	1.37760	-1.26524
O	-1.70499	-1.12915	-1.63446
C	-2.29617	-0.28364	0.85919
F	-2.06056	0.77482	1.59698
F	-3.56876	-0.53284	0.77392
F	-1.63364	-1.31888	1.31863
H	0.45976	-1.75035	-0.80785
N	2.68103	0.64924	0.19553
C	3.99202	0.51150	0.44102
C	4.28025	-0.85125	0.21177
H	4.62707	1.33952	0.74762
H	5.25315	-1.33004	0.31560
N	3.19522	-1.52467	-0.16089

40 Energy: -2418398.5235795

C	0.50762	1.27290	-0.28816
C	-0.85608	1.33299	-0.07758
C	-0.99379	-1.07703	-0.40792
C	0.34428	-1.09411	-0.60689
N	1.07518	0.08274	-0.53538
H	-1.61965	-1.96817	-0.45591
H	0.89700	-2.00289	-0.84531
S	2.83933	0.02054	-0.82316
O	3.04656	-1.15786	-1.60542
O	3.20342	1.36203	-1.16023
C	3.35273	-0.31779	0.94635
F	2.81019	-1.45837	1.30243
F	4.65021	-0.37319	0.98906
F	2.88918	0.66101	1.68757
H	1.13377	2.16906	-0.27193
N	-1.60543	0.13063	-0.13805
C	-2.88117	0.47710	0.09491
C	-2.87082	1.88202	0.29298
H	-3.74234	2.49954	0.50844
N	-1.64471	2.37210	0.18408
Br	-4.27532	-0.71655	0.12149

41 Energy: -1014905.4262055

C	0.28100	-0.42574	0.62930
C	-1.03042	-0.09089	0.31940
C	-0.27897	2.10989	-0.38733
C	0.98722	1.74520	-0.07578
N	1.24182	0.47646	0.41910
H	-0.53179	3.09739	-0.77221
H	1.84077	2.41616	-0.17459
S	2.94191	0.03677	0.83526
O	3.59881	1.28753	1.04880
O	2.81684	-1.05181	1.75321
C	3.41474	-0.63511	-0.85124
F	3.26114	0.33779	-1.71811
F	4.64875	-1.03400	-0.79268
F	2.59824	-1.62482	-1.12223
H	0.54301	-1.40178	1.04863
N	-1.29749	1.19877	-0.20156
C	-2.62291	1.25741	-0.40987
C	-3.10964	-0.00015	-0.00874
N	-2.14349	-0.80022	0.42786
H	-3.13604	2.13056	-0.80733
C	-4.55803	-0.43892	-0.05201
F	-4.69788	-1.49110	-0.83577
F	-5.29430	0.55926	-0.53191

F	-4.98433	-0.74398	1.15903

42 Energy: -2418377.8134390

C	1.28386	0.24174	-0.04194
C	2.97802	1.94782	0.28924
C	2.01545	2.93153	0.17531
H	4.04272	2.12046	0.46598
H	2.27164	3.98531	0.25878
S	-1.34594	0.93662	-0.79374
O	-1.87485	2.25302	-0.99113
O	-1.21297	-0.07145	-1.79365
C	-2.12211	0.24109	0.80488
F	-2.87877	1.18245	1.28713
F	-2.80798	-0.81023	0.46973
F	-1.16082	-0.05754	1.64547
N	0.35208	1.25604	-0.19353
C	0.70266	2.54352	-0.08299
H	-0.09957	3.27089	-0.23531
C	2.70235	-1.43509	0.19390
H	3.13683	-2.43170	0.26893
N	2.61176	0.66329	0.18178
N	3.45148	-0.36452	0.31395
C	1.32307	-1.13534	-0.02258
Br	0.00878	-2.43174	-0.18319

43 Energy: -828271.4207100

C	1.33322	-0.12783	-0.12895
C	2.71446	1.85096	0.32313
C	1.58669	2.64464	0.08269
H	3.67339	2.30181	0.58651
H	1.62555	3.73086	0.14643
S	-1.39619	0.15587	-0.85691
O	-2.04316	1.33468	-1.34315
O	-1.23301	-1.06121	-1.58669
C	-2.05112	-0.18647	0.86694
F	-2.14828	0.96379	1.48097
F	-3.20485	-0.77363	0.75269
F	-1.18399	-0.96060	1.48611
C	3.46469	-0.63594	0.38406
H	4.52010	-0.63641	0.65004
N	1.52993	-1.45977	-0.13433
N	0.24974	0.68945	-0.36254
C	0.39476	2.04460	-0.25566
H	-0.50163	2.62660	-0.47486
C	2.58298	0.47679	0.21642
N	2.82460	-1.74478	0.17384
C	0.69198	-2.63098	-0.41472
H	-0.23923	-2.59002	0.15397
H	0.47933	-2.70318	-1.48578
H	1.28913	-3.48625	-0.08722

44 Energy: -2432991.5466800

C	0.24514	-0.06369	-0.30991
C	1.54202	2.00345	0.00651
C	0.34871	2.72790	-0.09273
H	2.50261	2.49909	0.16657
H	0.32810	3.81373	-0.01630
S	-2.55159	0.09080	-0.78194
O	-3.28872	1.23028	-1.23312
O	-2.40177	-1.12939	-1.51078
C	-3.07810	-0.24224	0.98651
F	-3.12378	0.91177	1.60062
F	-4.24261	-0.81981	0.95921
F	-2.17645	-1.02028	1.54948
C	2.50432	-0.37801	-0.03312
N	0.49552	-1.39258	-0.34298
N	-0.90085	0.69349	-0.42014
C	-0.83121	2.05810	-0.30959

H	-1.77954	2.58244	-0.42598
C	1.48911	0.62622	-0.10246
C	-0.34831	-2.58424	-0.50923
H	-1.22034	-2.54271	0.14553
H	-0.66964	-2.69158	-1.54969
H	0.26324	-3.44339	-0.21819
C	1.87361	-1.57070	-0.18052
H	2.28720	-2.57612	-0.18127
Br	4.31111	-0.06039	0.23334

45 Energy: -828297.0967409

C	2.33233	-0.32753	-0.05339
C	0.45686	1.11129	-0.37240
H	-0.10677	2.04233	-0.47357
S	-1.99013	0.03562	-0.84762
O	-2.21196	1.35764	-1.34744
O	-2.32047	-1.19675	-1.49280
C	-2.55788	-0.03856	0.93376
F	-2.04072	0.99629	1.55513
F	-3.85728	-0.00100	0.94979
F	-2.10359	-1.15551	1.45279
C	2.90463	1.90155	0.09631
N	3.64446	-0.24777	0.18979
N	1.62130	-1.44904	-0.21455
C	0.35590	-1.29062	-0.44717
H	-0.30236	-2.14939	-0.60652
C	1.80334	1.01204	-0.12361
C	4.55893	-1.37564	0.33173
H	3.98572	-2.29785	0.19059
H	5.34659	-1.30763	-0.42852
H	5.00457	-1.36482	1.33399
C	3.99214	1.09696	0.28139
H	5.02738	1.36985	0.47824
N	-0.23844	-0.05057	-0.52156
H	2.89172	2.98709	0.11466

References

[1] Y. Liu, Y. Bai, J. Zhang, Y. Li, J. Jiao, X. Qi, *Eur. J. Org. Chem.* **2007**, 6084–6088.

[2] Y. Shen, J. Chen, M. Liu, J. Ding, W. Gao, X. Huang, H. Wu, *Chem. Commun.* **2014**, *50*, 4292–4295.

[3] P. S. Fier, J. F. Hartwig, *J. Am. Chem. Soc.* **2014**, *136*, 10139–10147.

[4] Y. Huang, D. Guan, L. Wang, *Chinese J. Chem*. **2014**, *32*, 1294–1298.

[5] T. Sakamoto, S. Kaneda, S. Nishimura, H. Yamanaka, *Chem. Pharm. Bull.* **1985**, *33*, 565–571.

[6] T. N. Steinhauer, U. Girreser, C. Meier, M. Cushman, B. Clement, *Chem. Eur. J.* **2016**, *22*, 8301–8308.

[7] A. R. Katritzky, E. F. Scriven, S. Majumder, H. Tu, A. V. Vakulenko, N. G. Akhmedov, R. Murugan, *Synthesis* **2005**, 993–997.

[8] Z. Changyou, Z. Guoliang (Beigene LTD), WO2014206344 (A1), **2014**.

[9] K. Maeda, Y. Furukawa, S. Takii, (Nissan Chemical Industries), EP1671941 A1, **2006**.

[10] J. C. Fennewald, B. H. Lipshutz, K. Wu, M. Song, G. Y. Li, H. C. Shen, A. Krasovskiy, R. D. Gaston, R. C. Gadwood, *Green Chem.* **2014**, *16*, 1097–1100.

[11] U. Sheridan, J. F. Gallagher, J. McGinley, *Tetrahedron* **2016**, *72*, 8470–8478.

[12] W. K. Fife, *Heterocycles* **1984**, *22*, 93–96.

[13] O. Ovdiichuk, O. Hordiyenko, V. Medviediev, O. Shishkin, A. Arrault, *Synthesis* **2015**, *47*, 2285–2293.

[14] T. Cailly, F. Fabis, S. Rault, *Tetrahedron* **2006**, *62*, 5862–5867.

[15] H.-Z. Zhang, S. Kasibhatla, J. Kuemmerle, W. Kemnitzer, K. Ollis-Mason, L. Qiu, C. Crogan-Grundy, B. Tseng, J. Drewe, S. X. Cai, *J. Med. Chem.* **2005**, *48*, 5215–5223.

[16] K. Kumpan, A. Nathubhai, C. Zhang, P. J. Wood, M. D. Lloyd, A. S. Thompson, T. Haikarainen, L. Lehtiö, M. D. Threadgill, *Bioorg. Med. Chem*. **2015**, *23*, 3013–3032.

[17] J. J. Song, N. K. Yee, Z. Tan, J. Xu, and S. R. Kapadia, C. H. Senanayake, *Org. Lett.* **2004**, *6*, 4905–4907.

[18] P.-H. Ko, T.-Y. Chen, J. Zhu, K.-F. Cheng, S.-M. Peng, C.-M. Che, *J. Chem. Soc. Dalt. Trans.* **1995**, *79*, 2215–2219.

[19] D. N. Mai, R. D. Baxter, *Org. Lett.* **2016**, *18*, 3738–3741.

[20] K. Nienkemper, V. V. Kotov, G. Kehr, G. Erker, R. Fröhlich, *Eur. J. Inorg. Chem*. **2006**, 366–379.

[21] A. Xie, M. Cao, Y. Liu, L. Feng, X. Hu, W. Dong, *Eur. J. Org. Chem.* **2014**, 436–441.

[22] S. R. Banini, M. R. Turner, M. M. Cummings, B. C. G. Söderberg, *Tetrahedron* **2011**, *67*, 3603–3611.

[23] S. Guo, G. Wan, S. Sun, Y. Jiang, J.-T. Yu, J. Cheng, B. Chen, J. Cheng, *Chem. Commun.* **2015**, *51*, 5085–5088.

[24] K. Yamaguchi, N. Xu, X. Jin, K. Suzuki, N. Mizuno, J. Wang, L. Liu, Y. Fu, *Chem. Commun.* **2015**, *51*, 10034–10037.

[25] K. Sasaki, A. Tsurumori, T. Hirota, E. de Ruiter, R. Goldstein, C. Gude, J. W. F. Wasley, *J. Chem. Soc. Perkin Trans. 1* **1998**, *21*, 3851–3856.

[26] V. G. Ramsey, W. E. Baldwin, R. S. Tipson, *J. Am. Chem. Soc.* **1947**, *69*, 67–70.

[27] F. D. Popp, L. E. Katz, C. W. Klinowski, J. M. Wefer, *J. Org. Chem.* **1968**, *33*, 4447–4450.

[28] G. Heinisch, W. Dostal, *Heterocycles* **1986**, 24, 793–797.

[29] H. Yamanaka, S. Nishimura, S. Kaneda, T. Sakamoto, *Synthesis* **1984**, 681–683.

[30] H. Yamanaka, T. Sakamoto, S. Nishimura, M. Sagi, *Chem. Pharm. Bull.* **1987**, *35*, 3119–3126.

[31] A. Metzger, L. Melzig, C. Despotopoulou, P. Knochel, *Org. Lett.* **2009**, *11*, 4228–4231.

[32] G. Karmas, P. E. Spoerri, *J. Am. Chem. Soc.* **1956**, *78*, 2141–2144.

[33] P. Petiot, A. Gagnon, *Eur. J. Org. Chem.* **2013**, 5282–5289.

[34] K. Komeyama, Y. Nagao, M. Abe, K. Takaki, *Bull. Chem. Soc. Jpn.* **2014**, *87*, 301–313.

[35] A. Nakamura, M. Ono, H. Segawa, T. Takematsu, *Agric. Biol. Chem.* **1984**, *48*, 1009–1016.

[36] A. Hirsch, D. G. Orphanos, *Can. J. Chem.* **1966**, *44*, 1551–1554.

[37] D. Bhattacharjee, F. D. Popp, *J. Heterocycl. Chem.* **1980**, *17*, 433–437.

[38] E. Higashino, T. Takemoto, M. Hayashi, *Chem. Pharm. Bull.* **1985**, *33*, 1351–1359.

[39] Z.-K. Wan, S. Wacharasindhu, C. G. Levins, M. Lin, K.Tabei, T. S. Mansour, *J. Org. Chem.* **2007**, *72*, 10194–10210.

[40] L.-Y. Liao, X.-R. Kong, X.-F. Duan, *J. Org. Chem.* **2014**, *79*, 777–782.

[41] S. Duric, C. C. Tzschucke, *Org. Lett.* **2011**, *13*, 2310–2313.

[42] M. Zhang, R. Gao, X. Hao, W.-H. Sun, *J. Organomet. Chem.* **2008**, *693*, 3867–3877.

[43] S. Akerboom, J. J. M. H. van den Elshout, I. Mutikainen, M. A. Siegler, W. T. Fu, E. Bouwman, *Eur. J. Inorg. Chem*. **2013**, 6137–6146.

[44] Y. Zhao, D. G. Truhlar, *Theor. Chem. Acc.* **2008**, *120*, 215–241.

[45] M. J. Frisch et al. *Gaussian 09, Revision D.01 Gaussian Inc*., Wallingford CT.

[46] E. D. Glendening, F. Weinhold, *J. Comput. Chem.* **1998**, *19*, 593–609.

[47] a) A. D. Becke, *J. Chem. Phys.* **1993**, *98*, 5648; (b) C. Lee, W. Yang, R. G. Parr, *Phys. Rev. B*, **1988**, *37*, 785; (c) S. H. Vosko, L. Wilk, M. Nusair, *Can. J. Phys.* **1980**, *58*, 1200; (d) P. J. Stephens, F. J. Devlin, C. F. Chabalowski, M. J. Frisch, *J. Phys. Chem*. **1994**, *98*, 11623; (e) S. Grimme, J. Antony, S. Ehrlich, S. Krieg, *J. Phys. Chem.* **2010**, *132*, 154104.
